# Environmental metabolomics characterization of modern stromatolites and annotation of ibhayipeptolides

**DOI:** 10.1371/journal.pone.0303273

**Published:** 2024-05-23

**Authors:** George F. Neuhaus, Allegra T. Aron, Eric W. Isemonger, Daniel Petras, Samantha C. Waterworth, Luthando S. Madonsela, Emily C. Gentry, Xavier Siwe Noundou, Jarmo-Charles J Kalinski, Alexandros Polyzois, Julius C. Habiyaremye, Margaret A. Redick, Jason C. Kwan, Rosemary A. Dorrington, Pieter C. Dorrestein, Kerry L. McPhail

**Affiliations:** 1 Department of Pharmaceutical Sciences, College of Pharmacy, Oregon State University, Corvallis, Oregon, United States of America; 2 Skaggs School of Pharmacy and Pharmaceutical Sciences, University of California San Diego, La Jolla, San Diego, CA, United States of America; 3 Collaborative Mass Spectrometry Innovation Center, Skaggs School of Pharmacy and Pharmaceutical Sciences, University of California San Diego, La Jolla, CA, United States of America; 4 Department of Biochemistry and Microbiology, Rhodes University, Makhanda, South Africa; 5 Division of Pharmaceutical Sciences, University of Wisconsin, Madison, WI, United States of America; Cairo University, EGYPT

## Abstract

Lithified layers of complex microbial mats known as microbialites are ubiquitous in the fossil record, and modern forms are increasingly identified globally. A key challenge to developing an understanding of microbialite formation and environmental role is how to investigate complex and diverse communities *in situ*. We selected living, layered microbialites (stromatolites) in a peritidal environment near Schoenmakerskop, Eastern Cape, South Africa to conduct a spatial survey mapping the composition and small molecule production of the microbial communities from environmental samples. Substrate core samples were collected from nine sampling stations ranging from the upper point of the freshwater inflow to the lower marine interface where tidal overtopping takes place. Substrate cores provided material for parallel analyses of microbial community diversity by 16S rRNA gene amplicon sequencing and metabolomics using LC–MS^2^. Species and metabolite diversities were correlated, and prominent specialized metabolites were targeted for preliminary characterization. A new series of cyclic hexadepsipeptides, named ibhayipeptolides, was most abundant in substrate cores of submerged microbialites. These results demonstrate the detection and identification of metabolites from mass-limited environmental samples and contribute knowledge about microbialite chemistry and biology, which facilitates future targeted studies of specialized metabolite function and biosynthesis.

## Introduction

The role of metabolism on microbial community structure at local spatial scales is a central question in microbial chemical ecology [1] that is most often approached by (meta)genomic analyses of microbial metabolic capacity [2,3]. The potential for chemical communication networks to structure microbial communities is well recognized [4], although *in situ* detection of metabolites at low, highly variable abundances in complex environmental samples remains difficult. In addition to limitations in instrument technology, a lack of characterized metabolite structures in centralized databases makes reliable annotation of the hundreds to thousands of mass spectrometric (MS) features detected by untargeted liquid chromatography mass spectrometry (LC–MS) methods challenging. The coupling of untargeted metabolomics to microbial community data and metagenomics for analysis of complex microbial communities was demonstrated recently by Tuttle et al. who deployed adsorption resins for direct sampling of metabolites in marine sediments [5]. These integrated analyses have been enabled by technological advances in high resolution mass spectrometry and associated data analysis tools [6]. In particular, computational mass spectrometry tools enable predictions of metabolite structure or structure class based on tandem mass spectrometry (MS^2^) fragmentation patterns, which require only nanograms of chemical extract from environmental samples. GNPS [7] is an open-access platform for comparative networking and searching of publicly shared MS^2^ spectra and available spectral databases. The SIRIUS5 suite [8] incorporates a collection of software tools to perform molecular formula, structure, and compound class predictions from MS^2^ spectra. Its core module SIRIUS analyzes isotope patterns and computes fragmentation trees to achieve molecular formula prediction, while the incorporated CSI:FingerID [9] uses these fragmentation trees to generate molecular fingerprints that facilitate structure predictions. These molecular fingerprints are also used by the module CANOPUS [10–12] to predict structural classes of metabolites through a deep neural network. Such integrated approaches enable the structural annotation of microbial metabolites directly from chemically complex and scarce environmental samples [4] even when no spectral match to characterized metabolite structures in centralized databases is found.

Microbialites are organo-sedimentary deposits formed by the metabolic activity of microbial communities that induce the precipitation of minerals and trapped sediments to form structures that can be amorphous, clotted (thrombolites) or layered (stromatolites) [13]. They are ubiquitous in the fossil record, dating back to the Archaean and Precambrian eras [14–17]. Extant microbialites occur in temperate (marine and freshwater) as well as extreme environments that include hypersaline lagoons [18]. They have been documented primarily from sites in Western Australia [19], the Bahamas [20], South/Central America [21], North America [22,23], and South Africa [24,25], with highly diverse microbial community structures and metabolisms. Supratidal microbialites on rocky coasts have been described since 2003 [26], and these distinct siliciclastic environments are characterized as representative of ancient microbialite beds along Precambrian coastlines [27]. The presence of modern microbialites provides an opportunity to investigate biomineralized microbial communities as a source of specialized metabolites, which are small organic molecules produced by organisms that increase survivability and fecundity but are not essential for survival.

In studies of microbialite communities, molecular approaches have provided phylogenetic profiles, based on 16S/18S SSU rRNA genes that identified taxonomically diverse consortia, representing different functional guilds, for example, photosynthesis, sulfate reduction, sulfide oxidation, heterotrophy, carbohydrate metabolism, and carbonate accretion [28–33]. Metagenomic studies of actively-growing microbialites have corroborated the presence of genes for the biochemical pathways expected for these functional guilds, and comparative metagenomic and metatranscriptomic analyses have been used to study the expression of genes associated with calcium carbonate accretion [34,35]. While metagenomic studies of microbialites may identify patterns of biosynthetic potential across different habitats, the context and levels of expression of metabolites and their potential ecological roles remain speculative. There are very few studies on the detection, characterization or biogenesis of small molecule metabolites produced by microbialites. Lipid profiles have been compared between microbialites and non-lithified microbial mats from the hypersaline Hamelin Pool in Shark Bay, Australia [36]. Putative assignment of cyanobacterial cyanopeptolin S and 21-bromo-oscillatoxin A by mass spectrometric analysis has been reported [37]. Production of natural microbial sunscreens, scytonemin and mycosporine-like amino acids, has also been studied in Shark Bay microbialites [38].

The first detailed report of coastal microbialites in South Africa documented the formation of tufa microbialites in upper intertidal rock pools fed by non-saline groundwater near the Kei River in the north of the Eastern Cape province [39]. Subsequently, similar occurrences of microbialites were recorded along 200 km (124 miles) of coastline in the Eastern Cape [24]. These formations are now known to be ubiquitous along the coastline of southern Africa, from Brandsebaai, South Africa, in the west to Tofo, Mozambique in the east [25]. The Schoenmakerskop tufa microbialite formation (S1 Fig) has been the subject of a number of hydrochemical [40], microbial [24,41], and associated metazoan studies [42–44]. These studies provided evidence of new and known cyanobacterial and other taxa, some of which are related to known producers of specialized metabolites with varied biological functions. Thus, we selected the Schoenmakerskop microbialite system to demonstrate the utility of untargeted metabolomics for analyzing environmental microbialite samples and to conduct an initial spatial survey of the study site that would inform future metagenomic deep sequence analyses.

At Schoenmakerskop, microbialites form a shallow barrage pool that is fed by diffuse freshwater input on the landward side and experiences marine tidal over-topping (flooding with seawater at high tide) at the seaward edge, leading to significant cycling in temperature and salinity [40]. Our primary goal was to assess the bacterial and small molecule composition of actively accreting microbialites by simultaneously comparing the diversity of prokaryotic species and chemistry in samples collected across the pool at established flag stations, spaced 3–5 m apart (S2 Fig). We sought to structurally classify and identify prominent and or new specialized metabolites that may be the same or different from those reported from cyanobacteria, heterotrophic bacteria, or other microbes in non-lithified microbial mats. Chemical extraction of substrate cores (S3 Fig) for LC–MS^2^ profiling and computational analyses to compare and structurally classify MS features, were paired with DNA extraction of substrate cores for phylogenetic analyses of the microbial community using 16S rDNA sequencing. Metabolomic analyses of the variation between samples collected across the pool led to the identification of a new class of depsipeptides, named ibhayipeptolides for ‘iBhayi’, which is the regional name by which Algoa Bay is known. Spearman rank correlations of MS features with bacterial 16S rRNA amplicon sequences based on abundances indicated an association of the ibhayipeptolides with cyanobacterial and bacteroidete taxa.

## Results and discussion

**Fig 1:**
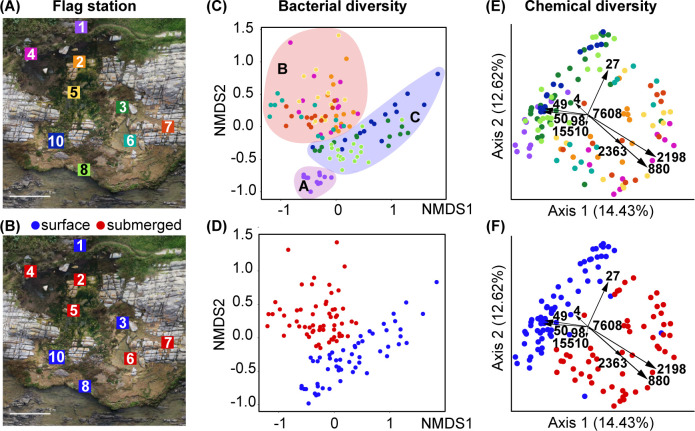
Schoenmakerskop microbialite collection site and component analyses. **(A)** Geographic information systems (GIS) image of the Schoenmakerskop barrage pool with superimposed numbered squares indicating the position of flagged collection stations (**1**–**8** and **10**) across the pool. At each of the nine flag stations, 12 to 18 substrate sample cores were collected (S4–S12 Figs). **(B)** GIS image of the Schoenmakerskop barrage pool displaying flag stations colored according to whether submerged or exposed at the surface. **(C)** NMDS plot of microbial 16S rDNA OTUs for all substrate cores colored by flag station, highlighting three visually apparent groupings, A: Flag station **1** (purple, freshwater inflow); B: Flag stations **2** and **4**–**7** (red, submerged); C: Flag stations **3**, **8**, and **10** (blue, surface). (**D)** NMDS plot (ANOSIM, R 0.47, p-value 0.0001, Bray-Curtis) indicating a significant difference between surface (blue) and submerged (red) flag stations; **(E)** Bray–Curtis PCoA biplot of the LC–MS data for all substrate cores, represented by filled circles colored by source flag station. All flag sites were significantly different from one another (PERMANOVA, pseudo-F 5.68, p 0.001, Bray-Curtis distance) **(F)** Bray–Curtis PCoA biplot of the LC–MS data for all substrate cores colored by exposure to surface water or submerged. A greater degree of separation is observed between submerged and flowing flag sites (PERMANOVA, pseudo-F 14.64, p-value 0.001, Bray-Curtis distance). Vectors indicate driver MS features associated with Bray–Curtis distances between samples and are described in Table 1.

**Fig 2:**
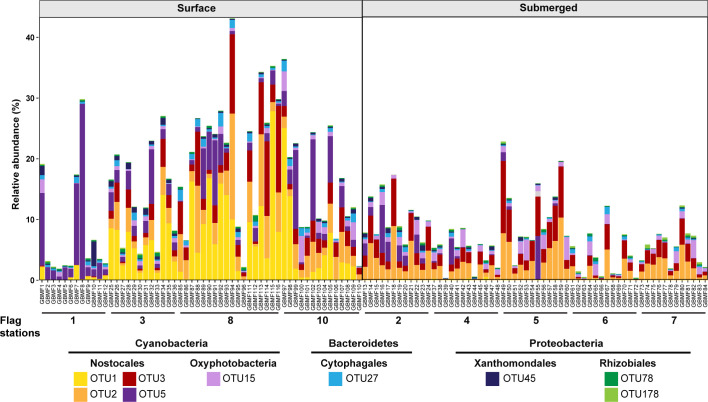
Analysis of 16S rRNA gene sequence amplicon data revealed the presence of nine conserved bacterial species across all microbialite core samples. Cores were grouped as either surface exposed or submerged, based on their source flag station, and these two groupings displayed distinct bacterial communities. In particular, cyanobacterial OTU1 characterized flag stations exposed to flowing water, and was largely absent from submerged flag stations.

Substrate cores obtained from collections made at two low tides on consecutive days were categorized according to their source location, as either submerged or surface exposed. Samples assigned to the submerged category were either completely submerged in standing water or at the water interface, while samples assigned to the surface exposed category were exposed to air or flowing water. In general, cores collected from the same flag station all fit into the same category of either surface exposed or submerged (flag stations **1**, **2**, **4**, **5**, **7**, **8**, and **10**; Figs 1A, 1B and S4–S12). Two exceptions were flag stations **3** (S6 Fig) and **6** (S9 Fig), where substrate cores collected ranged from surface exposed to submerged in standing water. Based on bacterial community compositions discussed in detail below (Fig 2), and for clarity in presentation of this work, flag station **3** is considered to be surface exposed and flag station **6** as submerged (Fig 1A and 1B).

A total of 1,567,727 reads, clustered into 12,498 OTUs, were recovered from all core samples collected across the stromatolite barrage pool (Fig 1A). The OTUs were classified within 613 different families, 361 orders, 150 classes and 51 bacterial phyla. At least 1,111 (8.9%) OTUs could not be classified beyond the bacterial kingdom. Approximately 30% of all OTUs were classified within Phylum Proteobacteria, and 16% classified within Phylum Bacteroidetes. The number of OTUs per phylum did not, however, correlate with the abundance of OTUs per phylum. The most abundant OTUs were classified within the Cyanobacteria phylum (33% relative abundance across all sites), with OTUs classified within the Proteobacteria and Bacteroidetes phyla accounting for 23% and 18% of total relative OTU abundance across all sites. Unclassified families in the Alphaproteobacteria, Gammaproteobacteria, and Bacteroidia, as well as the Omnitrophicaeota and Saprospiraceae families, accounted for 15% of all OTUs, with each consisting of approximately 3% of all OTUs. All other families accounted for 2% or less of all OTUs.

LC–MS^2^ raw data for all substrate cores was processed with MZMine2. Subsequent blank subtraction and removal of MS features found solely in the washout phase of the LC gradient resulted in a total of 15,752 MS features and 5,997 pairs of different ion species (e.g., [M+H]^+^, [M+Na]^+^) of the same molecule being identified, based on the correlation of chromatographic peak shapes for MS features [45]. Feature based molecular networking (FBMN) using GNPS resulted in 15,752 nodes with 25,894 edges (21,165 MS^2^ cosine scores greater than 0.7 and 4,729 MS^1^ annotations). Of the total number of nodes, 6,005 shared an edge with another node, leaving 9,747 singletons. Only 487 nodes (3.1%) were annotated with spectral similarities and precursor *m/z* matches to metabolites in GNPS spectral libraries. MS features were assigned to 1,287 different structural classes using CANOPUS.

### Bacterial diversity in microbialite cores across flag stations

**Fig 3:**
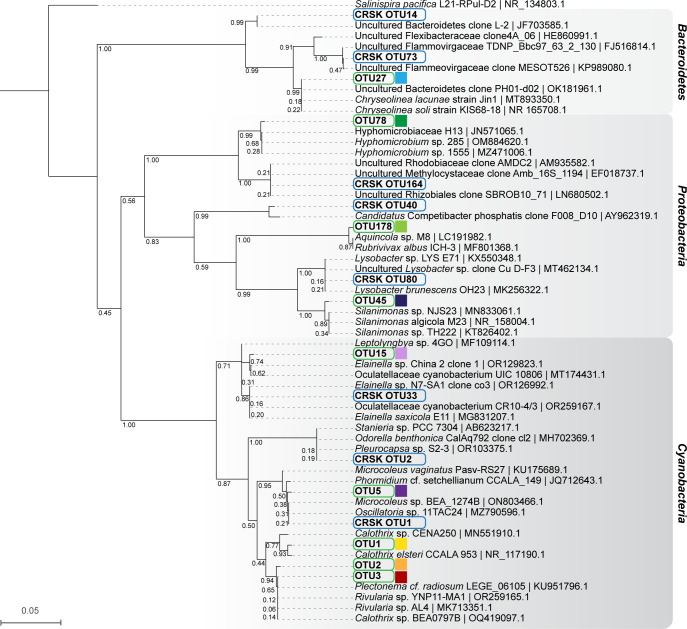
Phylogeny of operational taxonomic units (OTUs) conserved across all flag sites (highlighted with a green outline) relative to conserved OTUs from a previous study of bacterial communities in stromatolite formations from Schoenmakerskop and Cape Recife (highlighted with a blue outline). Colored blocks next to the conserved OTUs correlate with colors used in Fig 2 for easy reference. Additional reference sequences from the NCBI nr database have been included for approximate taxonomic classification. Phylogeny was inferred using the Maximum-likelihood method with 1000 bootstrap replicates. A spirochete was chosen as an outgroup. Bootstrap support is indicated on nodes and a scale has been provided for branch length, where the unit is the number of nucleotide substitutions per site.

Initially, we mapped the taxonomic diversity of the microbial communities to assess variability across the sampling stations, focusing on bacteria, which have been shown in a previous shotgun metagenomic study to comprise > 98% of the total microbial community [41]. We analyzed 16S rRNA gene amplicon libraries, acquired in parallel with LC–MS^2^ data, for samples at each flag station (Fig 1C and 1D). An assessment of beta diversity in the bacterial community by NMDS and ANOSIM (both Bray–Curtis distance) revealed that all flag stations (Fig 1C) were significantly different from one another (p < 0.05, S1 Table). However, the level of dissimilarity, as indicated by the R statistic, suggested separate grouping of A) flag station **1** samples from the freshwater inflow as unique and unlike all other sites, B) samples from flag stations **3**, **8** and **10**, which were all described as exposed to flowing water and shared low R values, and C) samples from generally submerged flag stations **4**, **5**, **6**, and **7**, with similarly low R values, and flag station **2** samples, which shared some similarity with those from **4** and **5** (see S1 Table). A canonical-correlation analysis (CCA, S13 Fig) of the bacterial OTUs showed that OTUs classified within the Nostocales class of Cyanobacteria were prominent members of the surface group of taxa, responsible for the differences observed between surface (blue) and submerged (red) communities (PERMANOVA, pseudo-F = 15.98, p = 0.001, Bray-Curtis distance). Phylogenetic classification of the 16S rRNA gene OTUs showed that almost all the samples were dominated by Cyanobacteria, followed by taxa classified within the Bacteroidetes and Proteobacteria. Vericobacteria and Chloroflexi OTUs were also abundant (S14 Fig). The bacterial communities were dominated by 20 OTUs, which accounted for between 50 to 80% of all reads across the sampling stations (S15 Fig); nine of these OTUs were conserved in all samples (Fig 2). Closer inspection revealed that the relative abundances of two conserved cyanobacterial OTUs classified as Nostocales (OTU1 and OTU5) were significantly more abundant in samples from the flag stations exposed to flowing water (Fig 1B: flags **1**, **3**, **8** and **10**; Fig 2). OTU1 shared the greatest nucleotide sequence identity with *Calothrix* species, and OTU5 was most closely related to mat-forming *Microcoleus* and *Phormidium* species (Fig 3). OTU5 is the only OTU that corresponds to a conserved OTU identified in a previous study of Schoenmakerskop and Cape Recife [41], suggesting that this species may be important for peritidal microbialite growth. Furthermore, *Microcoleus* species are reported to withstand high salinity [46], an attribute that may help these bacteria survive the periodic increases in salinity due to tidal over-topping of the stromatolite barrage pool. OTU2 and OTU3 are conserved across all stations and are dominant in samples from the submerged sites (flags **2**, **4**–**7**; Figs 1B and 2). OTU2 and OTU3 are closely related, and share greatest sequence identity with *Plectonema*, *Rivularia* and *Calothrix* species (Fig 3), which are all genera found in abundance in other microbialite formations that occur primarily in standing water bodies [47–51]. Additional data, such as metagenomic-assembled genomes, and associated transcriptomic data would be required to determine the effect that water flow rate has on the composition and behaviors of the mosaic of stromatolite-forming bacterial communities.

### Metabolite diversity of microbialite cores across flag stations

Our goal was to broadly characterize and compare the small molecule composition of substrate cores across the nine flag stations of the barrage pool. A molecular formula and prediction of compound structural class were obtained, using SIRIUS4 [8] and CANOPUS [11], respectively, for each MS feature detected in the pre-processed LC–MS^2^ data for all core samples. The *in silico* annotation tool CANOPUS utilizes deep neural network prediction based on fragmentation spectra to assign each detected MS feature to a superclass, class, and subclass. A preliminary assessment of differences between flagged stations across the barrage pool based on the CANOPUS output indicated that flag station **1** (landward freshwater inflow) and flag station **7** (mid-pool) displayed marked differences at the superclass and class level in metabolites present at each sampling station (S16 Fig).

Using Principal Coordinate Analysis (PCoA, Bray–Curtis distances) to assess the separation of MS features by individual flag site revealed that all flag stations (Fig 1E) were significantly different from one another (PERMANOVA, pseudo-F = 5.68, p = 0.001, Bray–Curtis distance). However, a greater degree of separation was observed between stations that were submerged in water versus those that were surface exposed, over which water was flowing (Fig 1F). Statistical significance of the separation between submerged and exposed stations was confirmed by PERMANOVA analysis, which revealed a pseudo-F score of 14.64 (p-value = 0.001, Bray–Curtis distance), in agreement with the bacterial community data.

**Fig 4:**
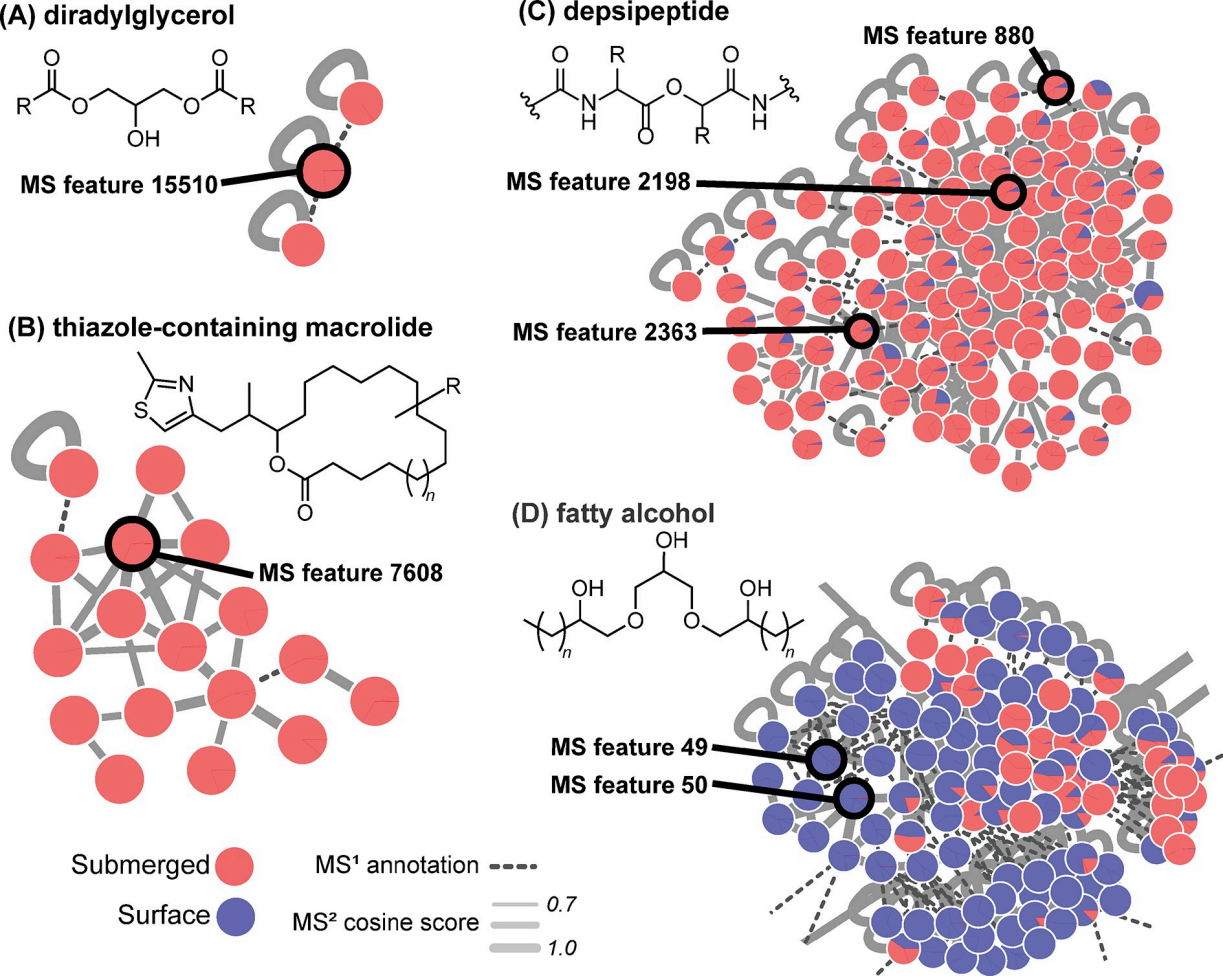
GNPS subnetworks containing MS features from Table 1 (PCoA biplot vectors). Feature-based and ion identity molecular networking in GNPS were used to create a global molecular network illustrating the chemical diversity in Schoenmakerskop microbialite substrate core samples. Nodes represent individual MS features and are connected by edges representing MS^2^ spectra with cosine similarity scores greater than 0.7 (solid light gray lines) or MS^1^ annotations of different ion species of the same molecule based on retention time and Pearson correlations of greater than 85% for chromatographic peak shape (dashed dark gray lines). Node color indicates contribution to an MS feature from either a surface exposed (blue) or a submerged (red) substrate core. Molecular family subnetworks that contain MS features responsible for separation of surface and submerged samples in the PCoA (Fig 1F) are displayed, and the potential drivers listed in Table 1 are circled and annotated in black with their MS feature ID. These MS features are predicted by CANOPUS to be, **(A)** diradylglycerols, **(B)** thiazole-containing macrolides, **(C)** depsipeptides, and **(D)** fatty alcohols, with representative chemical motifs as shown.

To understand the factors distinguishing submerged and surface exposed flag stations, we inspected the loading vectors in Fig 1F (Table 1). Discrete MS features can be visualized in the molecular network as nodes colored by submerged versus surface exposed metadata (Figs 4 and S17). Molecular families are observed as subnetworks of nodes connected by edges that represent MS^2^ spectra with cosine similarity scores greater than 0.7 (solid light gray lines) or MS^1^ annotations of different ion species of the same molecule (dashed dark gray lines). Ion species relationships are based on retention time matches and Pearson correlations of greater than 85% for chromatographic peak shape. Only subnetworks containing MS features responsible for separation of samples between surface and submerged stations are shown (Fig 4). Analysis of the MS^2^ spectra for ten top metabolite vectors with SIRIUS5 and CANOPUS (Table 1) predicted they belong to seven different chemical classes. Diradylglycerols, thiazole-containing macrolides, and depsipeptides were found primarily in the submerged cores, while fatty alcohols were present primarily in the surface exposed cores (Figs 4 and S17). Notably, the CANOPUS output for MS feature 7608 predicted epothilone (Table 1), although the large *m/z* (734.4649) and SIRIUS molecular formula prediction suggest that MS features in this subnetwork are larger thiazole-containing macrolides (Figs 4B and S17B). Further structure elucidation is precluded by low abundance and thus poor quality MS^2^ spectra (S18 Fig).

**Table 1:** Prominent MS features prioritized as PCoA vectors responsible for Bray-Curtis dissimilarity between submerged and surface (exposed) samples.

Ion cluster ID	MS Feature *m/z*, adduct	Subclass proposed by CANOPUS	Proposed molecular formula (SIRIUS5)
4	565.4028, [M+H]^+^	Tetraterpenoid	C_40_H_52_O_2_
27	535.2704, [M+H]^+^	Carboxylic acid Derivative	C_32_H_38_O_7_
49	539.4642, [M+Na]^+^	Fatty alcohol	C_31_H_64_O_5_
50	517.4825, [M+H]^+^		
98	183.1495, [M+H]^+^	Fatty amide	C_10_H_18_N_2_O
880	799.4619, [M+Na]^+^	Depsipeptide	C_44_H_64_N_4_O_8_
2198	777.4806, [M+H]^+^		
2363	785.4488, [M+Na]^+^	Depsipeptide	C_43_H_62_N_4_O_8_
7608	734.4649, [M+H]^+^	Epothilone	C_41_H_67_NO_8_S
15510	549.4484, [M+Na]^+^	Diradylglycerol	C_32_H_62_O_5_

We found that key MS features responsible for this separation, including MS feature IDs 2198, 880, and 2363, belong to the same family of depsipeptides (Fig 4C). Notably, a DEREPLICATOR [52] search matched MS feature ID 2198 with the depsipeptide serrawetin, for which the low score of 7 indicated a match in structural class only, further supporting the prediction by SIRIUS and CANOPUS (Table 1). Manual inspection of MS^2^ spectra for these MS features confirmed a peptidic molecular family and suggested they represent a new family of cyclic depsipeptides. Therefore, we targeted these prominent MS features for further structural characterization, assigning them the trivial name of ibhayipeptolides.

### Spatial distribution of depsipeptides across the barrage pool

**Fig 5:**
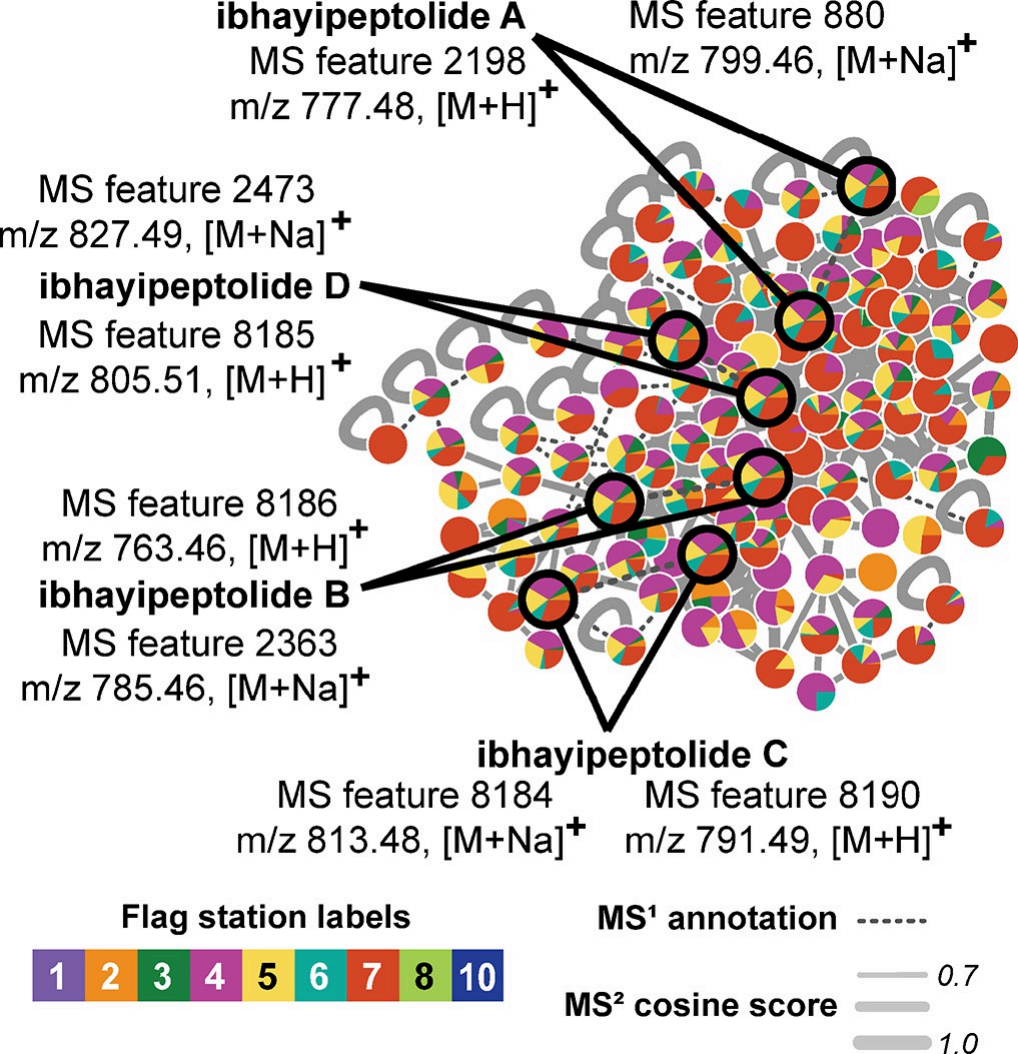
Distribution of depsipeptide MS features across barrage pool flag stations. GNPS feature-based molecular subnetwork for the ibhayipeptolide molecular family, incorporating ion identity networking of related mass adducts. MS features are represented as nodes labeled with MS^1^. Nodes are connected by edges that represent MS^2^ spectra with cosine similarity scores greater than 0.7 (solid light gray lines) or MS^1^ annotations of different ion species of the same molecule based on retention time and Pearson correlations of greater than 85% for chromatographic peak shape (dashed dark gray lines). Node contributions to the ibhayipeptolide family of depsipeptides are colored according to the source flag station of the parent core sample. MS features representing the major ibhayipeptolides (A-D) are circled in black, enlarged for clarity, and annotated with MS feature ID, *m/z* value, and assigned ion species.

Preliminary assessment of the chemical class differences between flag stations using CANOPUS indicated that stations **4**, **5**, and **7** were predicted to contain the highest abundance of depsipeptides, although this structural class was still present at flag stations **1** and **10**. CANOPUS plots of flag stations **1** and **7** are provided as representative examples in S16 Fig. GNPS feature-based molecular networking was used to confirm this CANOPUS prediction, and to visualize the number and extent of depsipeptide MS features across the flag stations (Fig 5). The molecular network revealed that MS features 2198 and 2363 are most abundant at flag stations **4** (pink), **5** (yellow), and **7** (dark orange). This trend in the presence of depsipeptides across the barrage pool may correspond with variation in the bacterial community.

### Planar structure elucidation of the ibhayipeptolides

**Fig 6:**
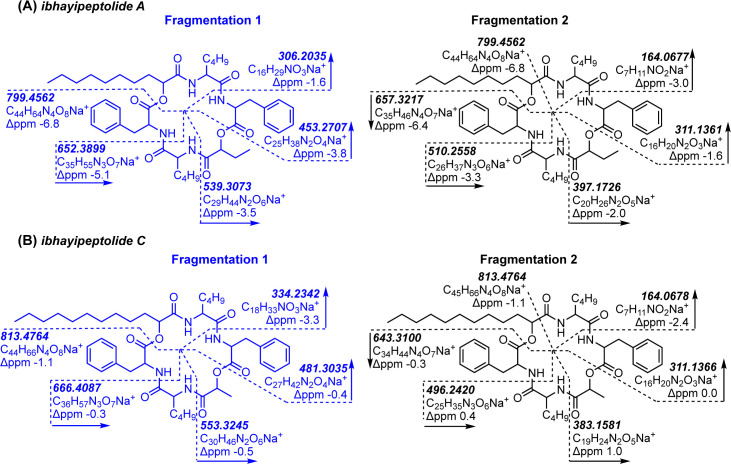
**Assignment of ibhayipeptolide planar structures from high resolution MS**^**2**^ **data. (A)** Proposed MS^2^ fragmentation pathways for ibhayipeptolide A (MS feature 2198) labeled with calculated mass and ppm error of fragment ions. **(B)** Proposed MS^2^ fragmentation pathways for ibhayipeptolide C (MS feature 8190) labeled with calculated mass and ppm error of fragment ions.

Ibhayipeptolides A (networked MS features 2198 [M+H]^+^ and 880 [M+Na]^+^) and B (MS features 8186 [M+H]^+^ and 2363 [M+Na]^+^) were targeted for additional structure elucidation and potential chromatographic isolation since they were prominent PCoA vectors representing metabolic differences between flag stations and could not be assigned as known compounds from their LC–MS^2^ data. Elucidation of their planar structures was assisted by comparison with the MS^2^ data for two additional congeners designated as ibhayipeptolides C (MS features 8190 [M+H]^+^ and 8184 [M+Na]^+^) and D (MS features 8185 [M+H]^+^ and 2473 [M+Na]^+^). We anticipated that knowledge of the ibhayipeptolide structures could be used to guide future investigations of the ecological role of these prominent microbialite metabolites. For ibhayipeptolide A (MS feature 2198), SIRIUS5 analysis of the molecular ion isotope (S19 Fig) and MS^2^ fragmentation patterns (S20 and S21 Figs) of both positive ([M+H]^+^ and [M+Na]^+^) and negative ionization ([M-H]^-^) spectra provided a molecular formula of C_44_H_64_N_4_O_8_ with 15 degrees of unsaturation. The identity and sequence of the six residues in this predicted depsipeptide (Fig 6A) were determined by manual analysis of the MS^2^ spectrum for the [M+Na]^+^ ion (MS feature 880). Two major MS fragmentation pathways were described for ibhayipeptolide A, one initiated by b-type cleavage of an ester and the other initiated by a-type ring cleavage of a α-C/C = O bond (Fig 6A). In pathway 1, ring-opening lactone cleavage of the sodiated ion is followed by sequential loss of amino and hydroxy acid residues beginning with Phe, then Lxx (representing Leu, Ile or MeVal), an α-hydroxybutyric acid (Hba) or equivalent moiety (C_4_H_6_O_2_), and a second Phe, yielding a sodiated b_2_ ion (*m/z* 306.2035, C_16_H_29_NO_3_Na^+^). In the congruent fragmentation pathway 2, an a-type sodiated ion (*m/z* 799.4562) results from ring cleavage at the α-C/C = O bond of a putative long chain α-hydroxy acid (C_10_H_18_O_2_). A neutral loss of *m/z* 142.1345 is explained by subsequent b-type cleavage and loss of hydroxydecanoic acid (Hda) from the linearized ion. Subsequent sequential loss of amino and hydroxy acid units culminates in a residual sodiated x_1_ ion (*m/z* 164.0677, C_7_H_11_NO_2_Na^+^) assigned as Lxx–CO. Further support for these fragmentation pathways was provided by the computed fragmentation tree in SIRIUS, for which each ion in a given pathway is found in a unique branch (S23 Fig). Together these analyses support the sequence of Phe–Lxx–Hba–Phe–Lxx–Hda.

**Fig 7:**
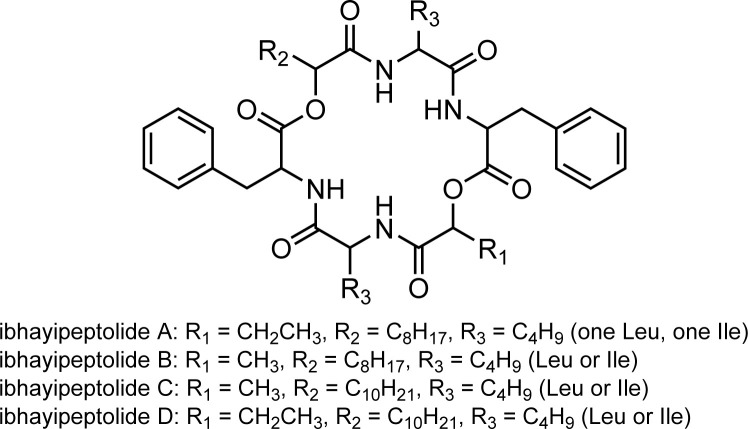
Molecular structure summary for ibhayipeptolides A-D. The ibhayipeptolide planar structures contain either α-hydroxybutyric acid (Hba, A and D) or lactic acid (B and C), and α-hydroxydecanoic acid (Hda, A and B) or α-hydroxydodecanoic acid (Hdda, C and D) residues as determined by analysis of MS^2^ fragmentations. Limited NMR data indicates the presence of both Leu and Ile residues in ibhayipeptolide A (S27 Fig), whereas these are unassigned in B-D because they are not distinguishable from MS^2^ data.

Analysis of the isotope pattern and MS^2^ fragmentation of [M+H]^+^ and [M+Na]^+^ with SIRIUS5 provided molecular formulas of C_43_H_62_N_4_O_8_ for ibhayipeptolide B (MS feature 8186), C_45_H_66_N_4_O_8_ for ibhayipeptolide C (MS feature 8190), and C_46_H_68_N_4_O_8_ for ibhayipeptolide D (MS feature 8185), respectively. Sufficient quantities of ibhayipeptolides could not be isolated for NMR analysis, yet nearly identical fragmentation patterns in the MS^2^ spectra for sodiated ions provided consistent planar structure assignments for all four congeners (S22, S31, S38 and S43 Figs). Ibhayipeptolide B (MS feature 8186) with *m/z* 785.4438 ([M+Na]^+^) differed from ibhayipeptolide A only in the loss of 72.0208 Da between the b_4_ and b_3_ ions, instead of 86.0365 Da, in pathway 1, indicating the presence of a lactic acid residue in ibhayipeptolide B in place of the Hba in ibhayipeptolide A (S31 Fig). Ibhayipeptolide C (MS feature 8184, *m/z* 813.4764 ([M+Na]^+^), also showed two major fragmentation pathways (Fig 6B). Fragmentation pathway 1 again indicates the presence of a lactic acid residue (b4—b_3_ = 72.0215 Da), as for ibhayipeptolide B. In fragmentation pathway 2 for ibhayipeptolide C, a loss of 170.1664 Da follows the initial a-type cleavage, instead of 142.1345 Da for ibhayipeptolide A, indicating substitution of a hydroxydodecanoic acid (Hdda) for the Hda moiety in ibhayipeptolides A and B. An Hba residue was evident (pathway 1, b4—b_3_ = 86.0365 Da) in the spectra for ibhayipeptolide D (MS feature 8185) with *m/z* 827.4910 ([M+Na]^+^), while an initial loss of 170.1658 Da in fragmentation pathway 2 was consistent with the presence of Hdda (S43 Fig). Together these data indicate that all four peptides share the same sequence and have α-hydroxy acids with varying sidechains (Fig 7).

The ^1^H NMR spectrum (S24 Fig) for ibhayipeptolide A (MS feature 2198) displayed two NH (δ_H_ 7.45, 7.48), ten aromatic (δ_H_ 7.1–7.3), two oxymethine (δ_H_ 5.15, 5.34), four amino acid α-H resonances (δ_H_ 3.6–4.6), and a congested aliphatic region (δ_H_ 0.6–2.4). A high quality, comprehensive NMR data set could not be obtained for ibhayipeptolide A due to a paucity of material isolated (~50 μg). Nevertheless, correlations observed in COSY (S25 Fig) and HSQC (S26 Fig) NMR experiments supported the identity of the six residues (S27 Fig, S2 Table). Spin systems for two Phe residues and an Hba, incorporating a deshielded α-H signal (δ_H_ 5.15), were present in the COSY spectrum. A Leu residue was delineated by COSY correlations between two CH_3_ doublets (δ_H_ 0.89, 0.93) and a γ-H multiplet (δ_H_ 1.58), along with a spin system including an NH (δ_H_ 7.45), an α-H (δ_H_ 4.19), and diastereotopic β-CH_2_ (δ_H_ 1.73, 1.82) signals. The presence of an additional oxymethine moiety (δ_H_ 5.34, δ_C_ 73.7) was consistent with a second hydroxy acid, as proposed from MS^2^ data (C_10_H_18_O_2_). This was further supported by two incomplete COSY spin systems suggesting a terminal methyl and α-hydroxy substituent. Finally, despite an overlapped upfield region in the ^1^H NMR spectrum, correlations supporting an Ile residue could be delineated. COSY spin systems incorporating a δ-CH_3_ triplet (δ_H_ 0.84, δ_C_ 10.9) correlated to diastereotopic γ-CH_2_ resonances (δ_H_ 1.29, 1.01, δ_C_ 26.1), and COSY-correlated NH (δ_H_ 7.48) and α-H (δ_H_ 3.67) signals were both observed. While both Leu and Ile are present, their relative location within the ring could not be determined without a high quality HMBC spectrum.

Relaxed substrate specificity of the NRPS adenylation domains [53–55] may be responsible for structural microheterogeneity (Fig 7) and co-elution of closely related metabolites. Microheterogeneity is supported by the large number of isobaric MS features in the molecular network: specifically, there are seven nodes for *m/z* 777.48 ± 0.03 with retention times between 7.7 and 8.0 min.

### Correlation of select metabolites with microbialite bacteria

**Fig 8:**
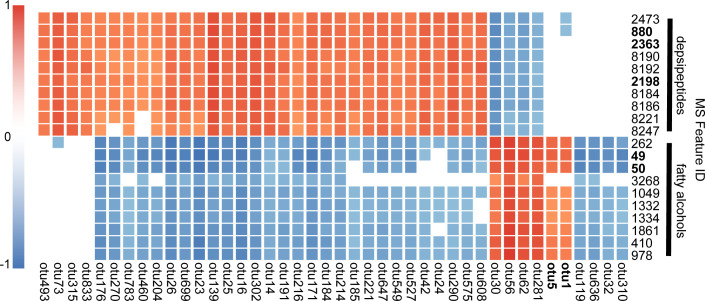
Correlation analysis heatmap for select metabolites with microbialite bacteria. Heatmap representation of top 20 most significant Spearman rank correlations and anticorrelations for both the ibhayipeptolide depsipeptides and fatty alcohol metabolites with bacteria present in microbialite substrate samples. Due to significance in driving microbial diversity and high correlation values, OTUs 5 and 1 (rank 23rd and 36th highest, respectively) were also included. Spearman correlations > 0, p < 0 are colored red, and anticorrelations > 0, p < 0 colored blue. MS features and OTUs that drive spatial metabolite and microbial diversity are bolded.

Given the variation in microbial community and metabolites between flag stations in the Schoenmakerskop barrage pool, we calculated (nonparametric) Spearman rank correlations between abundance of bacterial 16S rRNA OTUs and MS features (Fig 8) to identify groups of organisms associated with specific metabolites, whether positively or negatively correlated. These correlations in abundance may indicate producer or utilizer bacteria of specific metabolite families. It is also noteworthy that a fungal producer or utilizer of the targeted metabolites cannot be discounted in the absence of fungal ITS sequencing.

The ibhayipeptolide molecular family (e.g., MS features 2198, 880, and 2363) were significantly positively correlated with *Geitlerinema*_PCC-9228 (Cyanobacteria, OTU290), unknown Oxyphotobacteria_Incertae_Sedis (Cyanobacteria, OTU42), Cryomorphaceae (Bacteroidota, OTU302) and Chitinophagales (Bacteroidota, OTU25), while they were negatively correlated with *Phormidesmis*_ANT.L52.6 and Chamaesiphon_PCC-7430 (Cyanobacteria, OTUs 56 & 62 respectively), Anaerolineae A4b (Chloroflexota, OTU538), and Flavobacteriaceae (Bacteroidota, OTU92). It is noteworthy that none of these bacterial OTUs were dominant community members that could be expected to influence gross stromatolite morphology and there was no distinct stromatolite morphology associated with the highest abundance of depsipeptolides at flag stations 4–7. Interestingly, the fatty alcohol molecular family (e.g., MS features 49 and 50) also displayed significant but inverse correlations with some of the same organisms (Fig 8).

Cyanobacteria, heterotrophic bacteria, and fungi are all known to produce nonribosomal depsipeptides, comprising diverse amino and hydroxy acid residues, that exhibit a variety of biological properties. Many of these metabolites with alternating peptide and ester bonds comprise repeating depsipeptide oligomers. Cyclic hexadepsipeptides from cyanobacteria include antanapeptins [56], which like ibhayipeptolides consist of one short and one longer chain hydroxy acids, separated by a dipeptide. Unlike ibhayipeptolides, the antanapeptins and a variety of other cyanobacterial depsipeptides contain branched β-hydroxy acids [56–60]. There are many examples of branched longer chain hydroxy acids in the extensive set of known cyanobacterial cyclic depsipeptides and it is notable that the linear long chain α-hydroxy acid in the ibhayipeptolide structural motif appears unusual for a cyanobacterial metabolite [61,62]. Cyclic hexadepsipeptides containing long chain hydroxy acids from heterotrophic bacteria include the icosalides produced by *Burkholderia* species from diverse habitats [63]. A relevant group of pentadepsipeptides derived from both cyanobacteria and heterotrophic bacteria constitutes the unnarmicins [64,65] and solonamides [66], which comprise a general structural motif of one β-hydroxy acid cyclized with a tetrapeptide sequence of alternately repeating amino acids. Derived from a marine *Photobacterium* (Gammaproteobacteria), unnarmicins A and C possess β-hydroxy-octanoyl and -hexanoyl units, respectively, preceding a Leu-Phe-Leu-Phe sequence. In contrast, unnarmicin D was isolated from a marine *Trichodesmium* cyanobacterium and comprises a β-hydroxy-dodecanoyl moiety cyclized with a Gly-Tyr-Gly-Phe tetrapeptide. Solonamides A and B were also isolated from a marine *Photobacterium* and possess β-hydroxy-hexanoyl and -octanoyl units, respectively, preceding a Phe-Leu-Ala-Leu sequence. The solonamides have been investigated as biofilm inhibitors of *Staphylococcus aureus* given their inhibition of the accessory gene regulator (agr) quorum sensing system [66–69]. Cyclic depsipeptides reported from fungi mostly contain one or multiple relatively short and branched chain α-hydroxy acids, and hexadepsipeptides are the largest subgroup of this class [70,71]. A notable octadepsipeptide exception is verticilide, which is a cyclotetramer of the dipeptidol 2*R*-hydroxyheptanoic acid–N-methyl-L-Ala [72].

## Conclusion

The Schoenmakerskop microbialite system comprises mature microbialite formations with an abundance of superficial cyanobacteria as indicated by the diverse mosaic of bacterial taxa reported previously [24,41] and detected here (Figs 2, S14 and S15). We found that metabolites and microorganisms differ between core samples of microbialites submerged in the pool throughout the tidal cycle compared to surface microbialites exposed to flowing water or air between tides. The different chemical profiles observed between submerged and surface exposed core samples could be due to variation in UV exposure [73,74], oxidation, predator grazing, and or age and microbial composition of the microbialite accretions. The new ibhayipeptolide family of cyclic depsipeptides was most abundant in substrate cores of submerged microbialites, and correlated positively with cyanobacterial (OTUs 290, 42) and bacteroidete (OTUs 302, 25) taxa. This spatial survey of a mature stromatolite bed guided prioritization of substrate cores for future deep metagenomic sequencing, which is anticipated to reveal the ibhayipeptolide-producing organism, as well as biosynthetic templates for these and other MS features detected in low abundance and assigned to putative structural classes. Importantly, deep metagenomic sequencing may provide knowledge of the metabolic functional guilds of known and new organisms in the complex mosaic of microbialite communities and facilitate hypotheses for future studies on the role of chemical signaling in microbialite formation and maintenance. Given the microgram quantities of ibhayipeptolides and other more minor metabolites isolated by HPLC, investigation of their biological function awaits the ongoing chemical syntheses of representative structures.

## Materials and methods

### Inclusivity in global research

Additional information regarding the ethical, cultural, and scientific considerations specific to inclusivity in global research is included in the (S1 Checklist).

### Study site description

The Schoenmakerskop site (34°2’29” S 25°32’21” E; S1 Fig) is located adjacent to the metropole of Gqerberha (previously Port Elizabeth), close to human settlements and is exposed to moderate anthropogenic influences on ambient water chemistry [75]. Microbialite formations at this site begin from the freshwater inflow in the supratidal zone (Fig 1A). Freshwater from seeps feed through a short region of fluvial microbialites (Fig 1A, stations **1** and **4**) and then into a barrage pool (Fig 1A, stations **2**, **3**, **5** and **6**) separated from the subtidal zone by a microbialite plateau (Fig 1A, stations **7**, **8** and **10**). The formations are relatively flat, forming a shallow pool (mean depth 0.35 +/- 0.06 m) and the difference in elevation between the inflow formations and the plateau formations is ~1 m vertically, stretching over a horizontal distance of ~15 m. The flag stations range from ~3–5 m apart.

### Sample collection

Prior to field sampling, collection permits (RES2018/44; RES2021/81) were acquired from the South African Department Environmental Affairs (DEA) and the South African Department of Environment, Forestry and Fisheries (DFFE). The study site at Schoenmakerskop is public property and no protected species were sampled for this study. Microbialite substrate cores (S3 Fig) were collected from nine flagged stations (**1**–**8** and **10**, Figs 1A, 1B and S4–S12) in Schoenmakerskop barrage pool in the Eastern Cape of South Africa (S1 and S2 Figs) at low tide across two days (November 9 and 10, 2018; Permit No RES2018/44). Twelve substrate cores were collected from each of the flag stations **1**–**7**, while 18 cores were collected from flag station **8**, and 14 cores were collected from flag station **10**, for a total of 116 core samples. In each case a custom-made one-inch diameter, hollow metal corer was hammered into the substrate to a depth of about 5 inches with a mallet to provide cores at three different positions along the water flowpath (upper, middle, lower), to the left and right of each marker flag (S3 Fig). On each day, at least six cores were obtained per flag station (S4–S12 Figs). Additional cores were collected from stations **8** and **10** given their position at the edge of the seaward barrage wall. As cores were obtained, they were subsampled for separate chemical and DNA extractions. Subsamples for chemical extraction were placed in 20 mL glass vials on ice immediately in the field and frozen at -20°C within two hours of collection. Subsamples for DNA extraction were placed in Falcon tubes containing RNALater and flash-frozen in the field, then stored at –20°C within two hours of collection. Two sea water samples were also collected from an adjacent beach access (assigned as station **9**), as environmental controls for this study.

Two larger microbialite substrate samples for isolation and planar structure elucidation of metabolites were collected from flag station **7** in March 2021 (Permit No: RES2021/81), wrapped in aluminum foil and stored on ice immediately in the field until freezing at –20°C for subsequent chemical extraction.

### Sample extraction, data acquisition and processing

#### 16S amplicon sequencing

Genomic DNA (gDNA) was extracted from microbialite samples using the Zymo Quick-DNA™ Fecal/Soil Microbe Microprep Kit (Catalog No. D6012) as per the manufacturer’s instructions. DNA was extracted from approximately 50–100 mg of microbialite material, yielding 40 μL volumes containing between 2–4 μg of gDNA. Polymerase chain reaction (PCR) amplification of template DNA was performed with Miseq primers E517F (5’-CAGCAGCCGCGGTAA-3’) and E969-984R (5’-GTAAGGTTCYTCGCGT-3’), which target the V4-V5 region of the bacterial 16S rDNA gene (approximately 435 nt). Each 50 μL PCR reaction was prepared with 15–20 ng gDNA template, 10 μM of each primer, 5X PCR buffer (containing MgCl_2_), 10 μM DNTP’s, 0.5 units KAPA HIFI Hotstart DNA polymerase (cat no. 07958838001). MilliQ water was used to bring the total volume of the reaction to 50 μL. Thermal cycling parameters employed were as follows; initial denaturation at 95°C for 5 min followed by amplification with 5 cycles at 94°C for 30 s, 45°C for 20 s, 72°C for 1 min and additional amplification with 18 cycles at 94°C for 30 s, 50°C for 20 s and 72°C for 1 min, followed by a final elongation step of 72°C for 5 min. Samples were gel purified using the Bioline Isolate II gel and PCR kit (cat. no. BIO-52059). Samples were sequenced on an Illumina Miseq platform, generating ~250 nt amplicon libraries (only forward reads were determined to be of a high enough quality to use in analysis). NCBI BioProject accession number: PRJNA901469.

#### Chemical extraction and tandem mass spectrometry of microbialite core samples

Microbialite core samples were lyophilized at Rhodes University in South Africa in November/December 2018 and shipped to UC San Diego in January (received 01/2019). Of 116 vials containing core samples for the spatial survey, six vials were broken during shipping and were discarded. Approximately 1 cm^3^ (0.2 g) of each sample was placed in a scintillation vial and 5 mL 100% MeOH were added, including an empty vial as a control. The vials were left to stand for 1–2 h at room temperature before being placed at 4°C overnight. Aliquots of these methanol extracts (160 μL) were transferred to vials, and dried in vacuo for weighing. The range of extract weights was estimated by selecting forty representative samples, from all flag stations, judged by their color and apparent mass as low, medium and high mass. Aliquots (160 μL) of each MeOH extract were added to 96-well plates (Thermo 0.5 mL, U-bottom, PP). Controls included background seawater from a remote non-stromatolite site (#9), and methanol solvent and glass scintillation vial. The plates were dried *in vacuo* using a vacuum centrifuge (Centrivap, Labconco) and resuspended in 200 μL 9:1 MeOH/H_2_O per well. LC–MS^2^ analysis of the 2018 core sample collection was performed as described previously [76]. In short, 10 μL of each sample were injected into a Vanquish UHPLC system coupled to a Q-Exactive quadrupole orbitrap mass spectrometer (Thermo Fisher Scientific, Bremen, Germany). For the chromatographic separation, a reversed phase C18 porous core column (Kinetex C18, 150 x 2.1 mm, 1.8 μm particle size, 100 Å pore size, Phenomenex, Torrance, USA) was used. For gradient elution a high-pressure binary gradient system was used. The mobile phase consisted of solvent A H_2_O + 0.1% formic acid (FA) and solvent B acetonitrile (ACN) + 0.1% FA, and the flow rate was 0.5 mL/min. After injection, the samples were eluted with a linear gradient from 0–1 min at 5% B, 1–4 min 5–60% B, 4–10 min 60–99% B, followed by a 3 min washout phase at 99% B and a 2 min re-equilibration phase at 5% B. Data dependent acquisition (DDA) of MS^2^ spectra was performed in positive mode. Electrospray ionization (ESI) parameters were set to 52 AU sheath gas flow, 14 AU auxiliary gas flow, 0 AU sweep gas flow and 400°C auxiliary gas temperature. The spray voltage was set to 3.5 kV and the inlet capillary to 320°C. 50 V S-lens level was applied. MS scan range was set to *m/z* 150–1500 with a resolution at *m/z* 200 (R_*m/z* 200_) of 17,500 with one micro-scan. The maximum ion injection time was set to 100 ms with automated gain control (AGC) target of 5E5. Up to 5 MS^2^ spectra per MS^1^ survey scan were recorded in DDA mode with R_*m/z* 200_ of 17,500 with one micro-scan. The maximum ion injection time for MS^2^ scans was set to 100 ms with a AGC target of 5E5 ions. The MS^2^ precursor isolation window was set to *m/z* 1. Normalized collision energy was set to a stepwise increase from 20 to 30 to 40% with z = 1 as default charge state. MS^2^ scans were triggered at the apex of chromatographic peaks within 2 to 15 s from their first occurrence. Dynamic precursor exclusion was set to 5 s. Ions with unassigned charge states were excluded from MS^2^ acquisition as well as isotope peaks.

For MS^2^ data analysis, raw spectra were converted to.mzXML files using MSconvert (ProteoWizard). MS^1^ and MS^2^ feature extraction was performed using MZmine2.37, an IIN enabled version. The parameters used in MZmine2 are listed in S3 Table. The MS feature table.csv and.mgf files were exported and uploaded to GNPS (gnps.ucsd.edu) for feature-based molecular networking (FBMN). For spectrum library matching and spectral networking the minimum cosine score to define spectral similarity was set to 0.7. The precursor and fragment ion mass tolerances were set to 0.01 Da, minimum matched fragment ions to 6 and minimum cluster size to 1 (MS cluster off). Molecular networks were visualized with Cytoscape 3.7.2 [77] and node information was enriched with the MS^1^ peak areas from the MS feature table. The link to the GNPS job is as follows:

https://gnps.ucsd.edu/ProteoSAFe/status.jsp?task=90288a3e0cb44704bb1df00b440acba1

### Data analysis

#### 16S amplicon data analysis

Amplicon reads were curated using Mothur [78]. Reads with a quality window average below 20, length shorter than 250 bases or greater than 500 bases, any ambiguous bases, or homopolymeric runs greater than 7, were removed from the dataset. Chimeric sequences were identified using VSEARCH and were removed from the dataset. The remaining reads were classified against the SILVA database (v138.1) and all reads classified as Chloroplasts, Mitochondria, Unknown, Archaea or Eukaryota were removed. Sequences were aligned and all that did not fit into the region into which 95% of all sequences were aligned were removed. Aligned sequences were checked again for chimeric sequences which were removed upon identification. Singletons were removed to reduce computational expense. While some believe that this may bias the analysis in terms of removing spurious sequences [79], there is evidence to suggest that even when extremely rare species are the focus of research, removal of singletons aids in chimera removal, and increases accuracy of alpha and beta diversity while decreasing computational requirements [80]. Sequences were once again filtered to remove any reads classified as Chloroplasts, Mitochondria, Unknown, Archaea or Eukaryota as anecdotal experience has shown that some contaminants, primarily “unknowns”, are still present at this stage and a second sweep is required to thoroughly clean up the dataset. The remaining sequences were clustered into operational taxonomic units (OTUs) at a distance of 0.03 and counted per sample. Representative sequences for each OTU were similarly extracted and classified against the SILVA database (V138.1). OTU counts per sample were converted to relative abundance and used to generate non-metric multidimensional scaling (NMDS) plots in R using the vegan, plotly, and ggplot packages. Associated pairwise Analysis of Similarity (ANOSIM) scores were calculated for the different groups of samples (e.g., samples that were submerged or exposed to flowing water) using the vegan package in R. Canonical-correlation analysis (CCA) of bacterial OTUs was performed on normalized data using custom functions adapted from [81] and available at https://userweb.eng.gla.ac.uk/umer.ijaz/bioinformatics/ecological.html, which utilizes vegan [82] in R. Results were visualized in R using ggplot2 v3.4.4 (S13 Fig) [83].

Relative abundance plots of bacterial OTUs in the stromatolite core samples (Figs 2, S14 and S15) were generated using ggplot2 v3.4.4 in R [83]. Phylogeny of the conserved OTUs in Schoenmakerskop, relative to reference sequences and conserved OTUs from a previous study, was inferred using maximum likelihood (Fig 3). Briefly, conserved OTU sequences from this study and a previous study were aligned against the NR database using NCBI BLASTn [84,85] to find reference sequences. A spirochete sequence was chosen as an outgroup. All sequences were aligned using muscle (v 5.1) [86]. The alignment was then used to build a phylogenetic tree in MEGA 11 [87], using the Maximum Likelihood method with 1000 bootstrap replicates. The Tamura-Nei model was used, with uniform rates amongst sites where all sites were used. The initial tree was generated using the Neighbor-Joining method and the Nearest-Neighbor-Interchange was used for the heuristic method. The bootstrapped tree was visualized in iTol [88] and re-rooted at the outgroup.

#### LC–MS^2^ data preparation for analysis

The LC–MS^2^ data table was exported from MZmine2 and was further processed in the statistical software R (version 4.1.2) using modified code from a previously published analysis [89]. Blanks were subtracted from this table with a cutoff of 0.3 (i.e., MS features with a ratio of mean intensity in blanks vs mean intensity of MS features less than 30% were considered background noise and removed). After blank subtraction, the data were normalized by total ion count. The blank subtracted, normalized data were used for subsequent analysis in QIIME2.

#### LC–MS^2^ data analysis in QIIME 2

Subsequent visualization and statistical analysis of LC–MS^2^ data (Fig 1) was performed in QIIME2 (Bolyen et al. 2019). Unsupervised principal coordinate analysis (PCoA) was performed using the Bray-Curtis distance and the qiime beta and diversity pcoa plugin. PERMANOVA analysis was performed to quantify the significance of separation. All code is provided in the accompanying Zenodo repository (see Data Availability).

#### Spearman rank correlation analysis of MS features and 16S rRNA sequence data

The tables of MS feature abundances and bacterial OTU counts were used to generate a correlation analysis visualized as a heatmap (Fig 8). Prior to correlation, MS features observed in less than 10% of samples were removed. Amplicon sequencing data was transformed to proportions by dividing the counts for each sample by the total sequencing depth of the sample. Both datasets were mean centered and scaled, and combined in a single input file. Spearman rank correlations were calculated between each pair (microbe~metabolite) of MS features using R package “Hmisc”. P values were adjusted according to the Benjamini-Hochberg method to control the false discovery rate (FDR). FDR < 0.05 was considered significant. This linear statistical correlation analysis method was chosen over newer neural networking covariance methods due to the benchmarking performed by Quinn and Erb [90]. All code is provided in the accompanying Zenodo repository (see Data Availability).

#### Compound class prediction using SIRIUS and CANOPUS

Molecular formulas were assigned using the SIRIUS GUI. MzMine2 was used to generate and export an.mgf file specific for SIRIUS4 analysis of the 2018 core sample set. For individual analysis of the four major ibhayipeptolide depsipeptides, HRMS and MS^2^ (20, 40 and 60 eV) spectra of both [M+H]^+^ and [M+Na]^+^ ([M-H]^-^ was also included for MS feature 2198) were manually uploaded as csv files to SIRIUS5. SIRIUS, CSI:FingerID, and CANOPUS analyses were performed with the parameters listed in S4 and S5 Tables.

### Compound isolation and structure elucidation

#### General experimental procedures

NMR data were acquired on a Bruker Avance III 800 MHz spectrometer equipped with a 5 mm TCI cryoprobe, with the residual solvent used as an internal standard (CDCl_3_, δ_H_ 7.26, δ_C_ 77.16 ppm). High resolution (HR)TOFMS (ESI^+^) and tandem MS data were recorded on an Agilent 1260 infinity II LC coupled to a 6545 QToF MS. The mobile phase consisted of ultra-pure H_2_O (A) and ACN (B) with 0.1% formic acid. A gradient method from 15% B to 90% B in 9 min at a flow rate of 0.4 mL/min was used. The column (Phenomenex Kinetex C_18_, 2.6 μm, 100 Å, 50 mm x 2.1 mm) was re-equilibrated before each injection and the column compartment was maintained at 40 ˚C throughout each run. Semi-preparative HPLC (Phenomenex Kinetex C_18_, 5 μm, 100 Å, 250 mm x 10 mm) utilized isocratic elution conditions or a gradient system with a flow rate of 4 mL/min on a Shimadzu LC-20AD HPLC system operating at room temperature, equipped with an SPD-M20A photodiode array detector. All samples were filtered through a 0.2 μm nylon filter or centrifuged at 14,000 rpm for 5 min before LC–MS and HPLC analysis. General reagents were from VWR International.

#### Extraction and chromatographic purification of depsipeptides

Two larger samples collected in March 2021 from flag station 7 for characterization of pure depsipeptides were deep frozen before lyophilization for 48 h and each processed separately according to the following general protocol for separate LC–MS profiling in case they varied in chemistry. The dry biomass (1.91 kg total for both samples) was crushed by mortar and pestle into pieces smaller than 0.5 cm. The resulting powdered biomass was extracted twice in DCM-MeOH (2:1) by soaking for 48 h at room temperature in the dark and without agitation. Approximately 2.8 L of solvent was used per kg of dry biomass. After each extraction, the supernatant was filtered through filter paper and concentrated under reduced pressure to afford two organic extracts (4.13 g total).

Each organic extract was initially fractionated into 10 fractions (A-G) by normal phase chromatography on a Teledyne ISCO CombiFlash R_f_ 200 system using a RediSep column (silica 40 g Gold). The mobile phase gradient used was 100% hexanes to 100% EtOAc over 23 min, holding at 100% EtOAc for 3.0 min, then ramping to 75% EtOAc-MeOH over 7 min and holding for 5 min. Due to the largest amount of ibhayipeptolide A present, fraction B (7–15 min) was split into 5 subfractions (B1-B5) using RP_18_ chromatography on the same Combiflash system. A RediSep column (30 g HP C_18_) was used with a gradient of MeOH-H_2_O (50% for 2 min before ramping to 100% MeOH over 5 min and holding for 9 min). Ibhayipeptolides A and B (MS features 2198 and 8186) were in highest abundance in subfraction B3 (4–8 min). Ibhayipeptolides A (t_R_ 9.1 min) and B (t_R_ 8.0 min) were isolated by semi-preparative HPLC (Kinetex C_18_ 250x10 mm) from subfraction B3 utilizing a shallow gradient (90% ACN to 100% ACN over 17 min). Ibhayipeptolides A (80% ACN, Kinetex C_18_ 100x4.6 mm, 8.6–9.5 min) and B (80% ACN, Kinetex C18 250x10 mm, 17.1–17.7 min) were purified further using isocratic HPLC conditions. Less than 100 μg of each depsipeptide were isolated.

#### Analytical data for ibhayipeptolides with assigned planar structures

*Ibhayipeptolide A (MS feature 2198)*: white amorphous solid; HRESI^+^MS: *m/z* 777.4806 [M+H]^+^ (calcd for C_44_H_65_N_4_O_8_^+^, 777.4797; Δ ppm = 1.2), *m/z* 799.4562 [M+Na]^+^ (calcd for C_44_H_64_N_4_O_8_Na^+^, 799.4616; Δ ppm = 6.8). *m/z* 775.4674 [M-H]^-^ (calcd for C_44_H_63_N_4_O_8_^-^, 775.4651; Δ ppm = 3.0). SIRIUS score: [M+H]^+^ 54.2%, [M+Na]^+^ 59.4%, [M-H]^-^ 40.2%.

*Ibhayipeptolide B (MS feature 8186)*: white amorphous solid; HRESI^+^MS: *m/z* 763.4666 [M+H]^+^ (calcd for C_43_H_63_N_4_O_8_^+^, 763.4640; Δ ppm = 3.4), *m/z* 785.4488 [M+Na]^+^ (calcd for C_43_H_62_N_4_O_8_Na^+^, 785.4460; Δ ppm = 3.6). SIRIUS score: [M+H]^+^ 87.4%, [M+Na]^+^ 60.3%.

*Ibhayipeptolide C (MS feature 8190)*: white amorphous solid; HRESI^+^MS: *m/z* 791.5013 [M+H]^+^ (calcd for C_45_H_67_N_4_O_8_^+^, 791.4953; Δ ppm = 7.6), *m/z* 813.4831 [M+Na]^+^ (calcd for C_45_H_66_N_4_O_8_Na^+^, 813.4773; Δ ppm = 7.1). SIRIUS score: [M+H]^+^ 18.5%, [M+Na]^+^ 49.1%.

*Ibhayipeptolide D (MS feature 8185)*: white amorphous solid; HRESI^+^MS: *m/z* 805.5135 [M+H]^+^ (calcd for C_46_H_69_N_4_O_8_^+^, 805.5110; Δ ppm = 3.1, *m/z* 827.4955 [M+Na]^+^ (calcd for C_46_H_68_N_4_O_8_Na^+^, 827.4929; Δ ppm = 3.1). SIRIUS score: [M+H]+ 92.3%, [M+Na]+ 85.1%.

## Supporting information

S1 Checklist Inclusivity in global research. (DOCX)

S1 Fig Map of the Nelson Mandela Bay area of South Africa. Schoenmakerskop is marked with a red dot. Landsat imagery courtesy of NASA Goddard Space Flight Center and U.S. Geological Survey.
(EPS)

S2 Fig GIS image of the Schoenmakerskop barrage pool. Numbered squares indicate the position of flagged collection stations (**1**–**8** and **10**) across the pool.
(TIF)

S3 Fig Hollow metal corer in field use and representative stromatolite substrate core. (TIF)

S4 Fig Flag station 1 substrate core map. (TIF)

S5 Fig Flag station 2 substrate core map. (TIF)

S6 Fig Flag station 3 substrate core map. (TIF)

S7 Fig Flag station 4 substrate core map. (TIF)

S8 Fig Flag station 5 substrate core map. (TIF)

S9 Fig Flag station 6 substrate core map. (TIF)

S10 Fig Flag station 7 substrate core map. (TIF)

S11 Fig Flag station 8 substrate core map. (TIF)

S12 Fig Flag station 10 substrate core map. (TIF)

S13 Fig CCA plot of bacterial 16S rRNA OTUs for all substrate cores colored by exposed or submerged flag stations. PERMANOVA, pseudo-F = 15.98, p = 0.001. Vectors indicate 16S rRNA OTUs associated with Bray–Curtis difference between substrate samples.
(TIF)

S14 Fig Phylogenetic classification and relative abundance of 16S rRNA gene OTUs in samples across the study site. All samples are dominated by Cyanobacteria, Bacteroidetes, and Proteobacteria.
(TIF)

S15 Fig Phylogenetic classification of the most dominant bacterial 16S rRNA gene OTUs. 20 OTUs accounted for between 50–80% of all the reads across samples, shown here classified to the rank of Order.
(TIF)

S16 Fig Sunburst plots of CANOPUS results from flag stations 1 and 7. Percentages based on MS feature chromatographic peak area. Expansions show class and subclass distributions for phenylpropanoids/polyketides and organic acids and derivatives.
(TIF)

S17 Fig Global molecular network using feature based and ion identity networking in GNPS for all 2018 Schoenmakerskop stromatolite core samples. Subnetworks containing driver MS features are labeled with the structural class predicted by CANOPUS (A-D).
(TIF)

S18 Fig MS^2^ spectrum of MS feature 7608. Annotated as epothilone by CANOPUS.
(TIF)

S19 Fig HRMS of ibhayipeptolide A. *m/z* 777.4806, MS feature 2198, [M+H]^+;^ *m/z* 799.4620, MS feature 880, [M+Na]^+^.
(TIF)

S20 Fig MS^2^ spectrum for [M+H]^+^ of ibhayipeptolide A. MS feature 2198.
(TIF)

S21 Fig MS^2^ spectra for [M+Na]^+^ of ibhayipeptolide A. MS feature 880.
(TIF)

S22 Fig Proposed fragmentation pathways for ibhayipeptolide A. Top) Predicted fragmentation with theoretical mass and ppm error of fragment ions. Bottom) MS^2^ spectra ([M+Na]+, 60eV) labeled with HR mass (fragmentation 1: blue dotted lines, fragmentation 2: black solid lines).
(TIF)

S23 Fig SIRIUS5 fragmentation tree for ibhayipeptolide A. (TIF)

S24 Fig 1H NMR spectrum of ibhayipeptolide A. CDCl_3_, 800 MHz, TCI, 3 mm tube.
(TIF)

S25 Fig COSY NMR spectrum of ibhayipeptolide A. CDCl3, 800 MHz, TCI, 3 mm tube.
(TIF)

S26 Fig HSQC NMR spectrum of ibhayipeptolide A. CDCl_3_, 800 MHz, TCI, 3 mm tube.
(TIF)

S27 Fig Key COSY NMR correlations supporting residues present in ibhayipeptolide A. (TIF)

S28 Fig HRMS of ibhayipeptolide B. *m/z* 763.4666, MS feature 8186, [M+H]^+^; *m/z* 785.4488, MS feature 2363, [M+Na]^+^.
(TIF)

S29 Fig MS^2^ spectra for [M+H]^+^ of ibhayipeptolide B (MS feature 8186). (TIF)

S30 Fig MS^2^ spectra for [M+Na]^+^ of ibhayipeptolide B (MS feature 2363). (TIF)

S31 Fig Proposed fragmentation pathways for ibhayipeptolide B. Predicted fragmentation with theoretical mass and ppm error of fragment ions (Top). MS^2^ spectra ([M+Na]+, 60eV) labelled with HR mass (Bottom; fragmentation 1: blue dotted lines, fragmentation 2: black solid lines).
(TIF)

S32 Fig SIRIUS5 fragmentation tree for ibhayipeptolide B. (TIF)

S33 Fig 1H NMR spectrum of ibhayipeptolide B. CDCl_3_, 800 MHz, TCI, 3 mm tube.
(TIF)

S34 Fig Multiplicity edited HSQC NMR spectrum for ibhayipeptolide B. CDCl_3_, 800 MHz, TCI, 3 mm tube.
(TIF)

S35 Fig HRMS of ibhayipeptolide C. *m/z* 791.5013, MS feature 8190, [M+H]^+^; *m/z* 813.4831, MS feature 8184, [M+Na]^+^.
(TIF)

S36 Fig MS^2^ spectra for [M+H]^+^ of ibhayipeptolide C (MS feature 8190). (TIF)

S37 Fig MS^2^ spectra for [M+Na]^+^ of ibhayipeptolide C (MS feature 8184). (TIF)

S38 Fig Proposed fragmentation pathways for ibhayipeptolide C. Top) Predicted fragmentation with theoretical mass and ppm error of fragment ions. Bottom) MS^2^ spectra ([M+Na]+, 60eV) labelled with HR mass (fragmentation 1: blue dotted lines, fragmentation 2: black solid lines).
(TIF)

S39 Fig SIRIUS5 fragmentation tree for ibhayipeptolide C. (TIF)

S40 Fig HRMS of ibhayipeptolide D. *m/z* 805.5135, MS feature 8185, [M+H]^+^; *m/z* 827.4955, MS feature 2473, [M+Na]^+^.
(TIF)

S41 Fig MS^2^ spectra for [M+H]^+^ for ibhayipeptolide D (MS feature 8185). (TIF)

S42 Fig MS^2^ spectra for [M+Na]^+^ for ibhayipeptolide D (MS feature 2473). (TIF)

S43 Fig MS^2^ fragmentation pathway for [M+Na]+ for ibhayipeptolide D. (TIF)

S44 Fig SIRIUS5 fragmentation tree for ibhayipeptolide D. (TIF)

S1 Table R-score matrix of beta diversity in 16S rDNA OTUs across flag stations. Flag station **1** is entirely unique (purple outline). Flag stations **3**, **8**, and **10** show R-scores all lower than 0.45, indicating similarity (blue outline). Flag stations **2** and **4**–**7** also show similarity (dark red outline), while flag station **2** is more similar to flag stations **4** and **5**, than **6** and **7** (light red outlines).
(XLSX)

S2 Table Proposed NMR chemical shift assignments for ibhayipeptolide A. CDCl_3_, 800 MHz, TCI probe, 3 mm tube.
(DOCX)

S3 Table Pre-processing settings for MS feature detection using MZmine2. (DOCX)

S4 Table SIRIUS5 and CSI:FingerID parameters for QToF. (DOCX)

S5 Table SIRIUS4 parameters for Thermo QExactive Orbitrap. (DOCX)
