# Supplementary material for: Environmental metabolomics characterization of modern stromatolites and annotation of ibhayipeptolides
Source: PLoS One. 2024 May 23;19(5):e0303273. doi: 10.1371/journal.pone.0303273 (PMC11115249; doi:10.1371/journal.pone.0303273)
Supplement: S1 Checklist — (DOCX) [file pone.0303273.s001.docx]

**S1 Checklist. Inclusivity in Global Research.**

**Ethical considerations, permits and authorship**

*This section is applicable to all research types.*

Provide details as to who granted permissions and/or consent for the study to take place in the Methods section of your manuscript. This should include the names of **all** ethics boards, governmental organizations, community leaders or other bodies that provided approval for the study. If individuals provided approval refer to these people by their role or title but do not list their name(s).

This information is reported in the Materials and Methods section under the subheading “Sample Collection”.

If there were any deviations from the study protocol after approval was obtained please provide details of these changes in the Methods section of your manuscript.
Did this study involve local collaborators that are residents of the country where the research was conducted or members of the community studied? If you do not have any authors from said communities, please provide an explanation for this below.

Researchers from Rhodes University are residents of South Africa and participated in the collection of samples and data, data analysis and manuscript writing and editing. All key contributors to the research presented here are included as co-authors. All authors meet the PLOS’ criteria for authorship.

Reported on page number: not applicable

Everyone listed as an author should meet PLOS’ criteria for authorship and all individuals who meet these criteria should be included in the author byline, rather than the acknowledgements. For further information please see the journal’s Authorship Policy.

**Non-human subjects research using specimens/ animals collected as part of the study, or those housed in archival collections. Examples include archaeology, paleontology, botany and zoology.**

Did the permission you obtained from a local authority to perform the study include an agreement on access to outputs and benefit sharing? This may include procedures to enable fair distribution of the benefits and resources arising from the research performed. Please include any details of Prior Informed Consent and Benefit Sharing Agreements obtained. These may be required by field-specific regulations, for example the Convention on Biological Diversity (CBD) and the associated Nagoya Protocol.

A comprehensive collaborative agreement was signed between Oregon State University, Rhodes University, University of California at San Diego, and University of Wisconsin Madison, for this collaboration funded by the Gordon and Betty Moore Foundation. Rhodes University holds the necessary integrated research permits (RES2018/44 and RES2021/81) from the South African Department of Environmental Affairs and South African Department of Forestry, Fisheries and Environment to collect environmental samples (stromatolites) for research purposes in accordance with the Nagoya Protocol on Access and Benefit-Sharing of the Convention on Biological Diversity, of which South Africa is a member. (https://www.cbd.int/countries/profile?country=za).

If the material used in your study was imported, please A) provide the year it was imported and B) indicate whether permits were obtained to import/export the materials used, C) provide details of any permits obtained. If this information is not available, please indicate this.

1. Stromatolite material was processed at Rhodes University, where DNA extraction and sequencing took place; small core samples and chemical extracts were transferred from South Africa to the United States in 2018 and 2021.
2. This material was transferred under research permits RES2018/44 and RES2021/81.

If you used archival specimens, please state how the material used in your study was acquired by the institute it is held in and provide details of any permits obtained for the original excavations/ sample collection. If this information is not available, please indicate this.

No archival specimens were used in this study.

How was the potential cultural significance of the materials collected in your study to local communities considered in your research design? Were Indigenous peoples and/or local researchers and institutions involved with archaeological excavations / collection of specimens? If so, please provide a description of their involvement.

Local researchers and institutions led the collection of all stromatolite samples collected for this study. They were involved in all planning, organization and field activities.

If your manuscript includes photographs of human remains please indicate whether authors obtained permission from descendants or affiliated cultural communities to do so.

Not applicable.
