# Supplementary figures and images for: Environmental metabolomics characterization of modern stromatolites and annotation of ibhayipeptolides
Source: PLoS One. 2024 May 23;19(5):e0303273. doi: 10.1371/journal.pone.0303273 (PMC11115249; doi:10.1371/journal.pone.0303273)

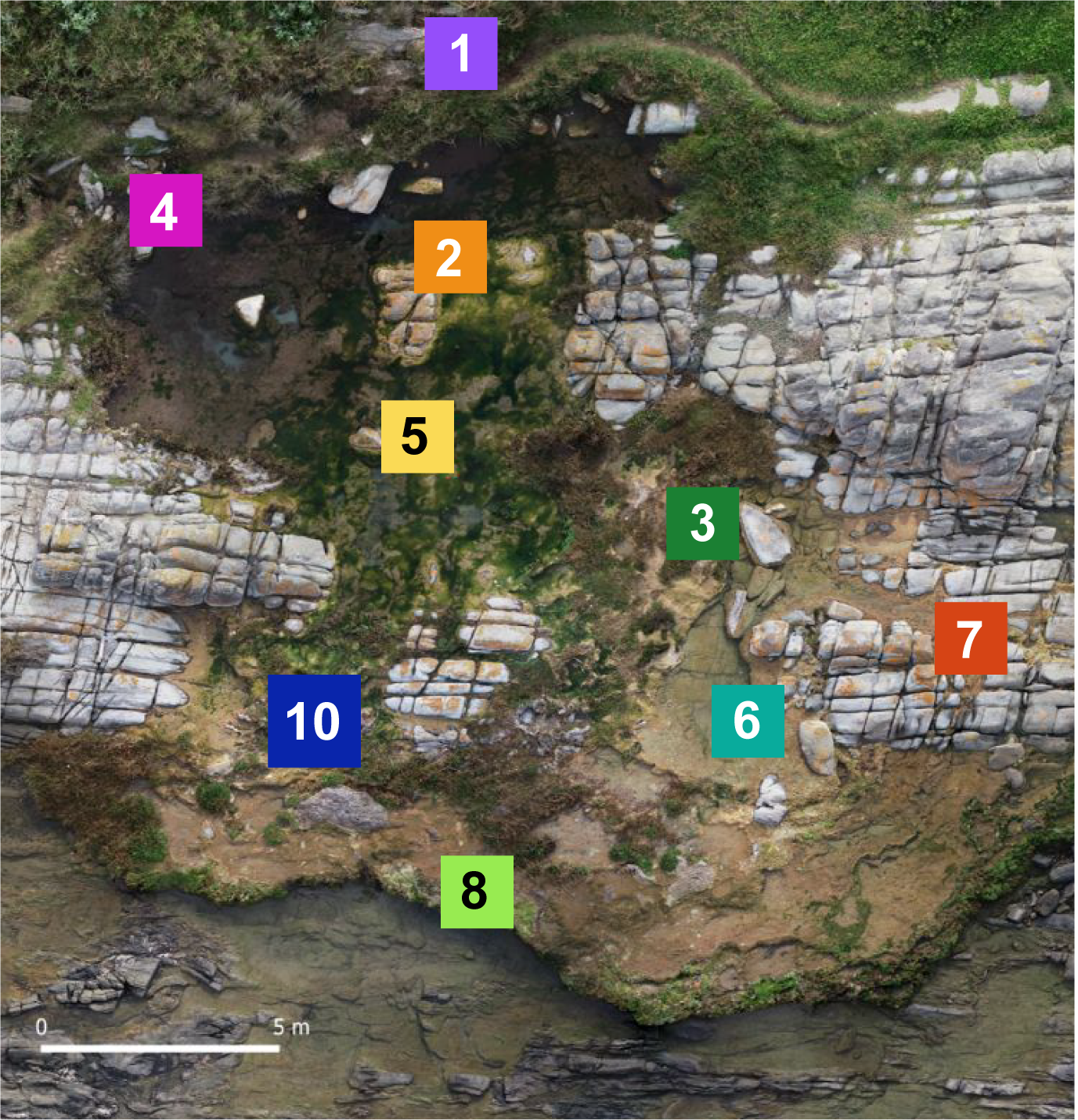

Supplement: S2 Fig — Numbered squares indicate the position of flagged collection stations (1–8 and 10) across the pool. (TIF) [file pone.0303273.s003.tif]

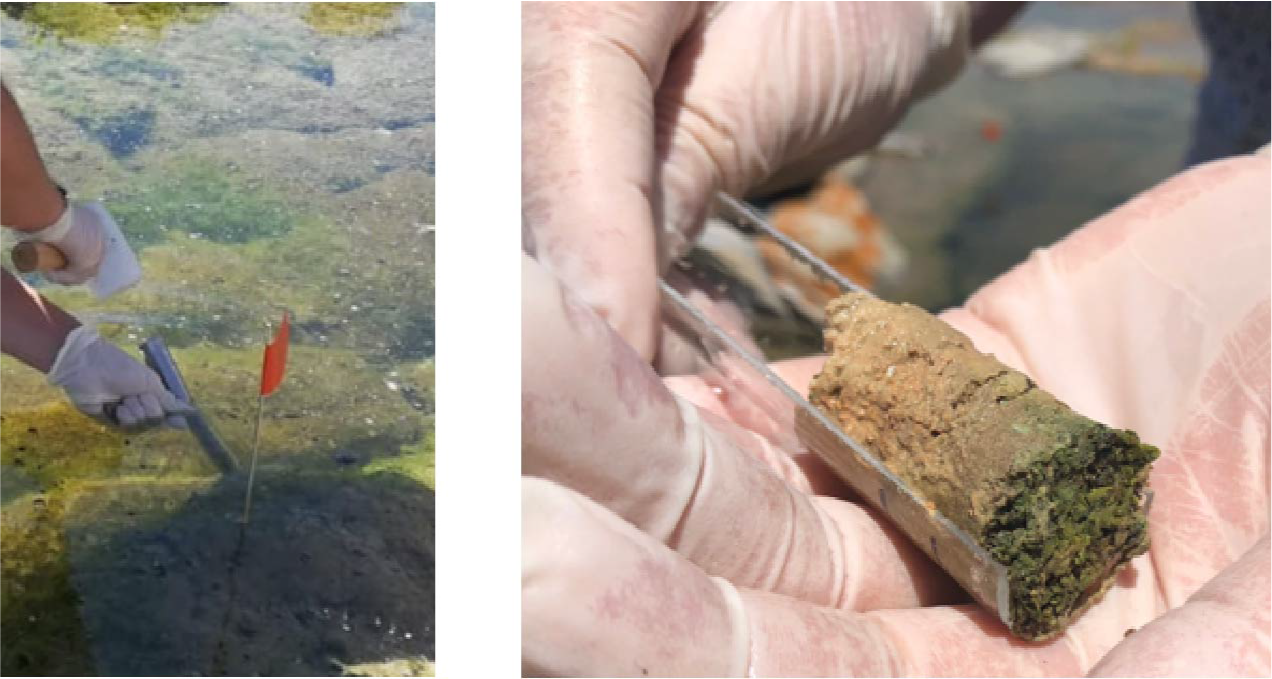

Supplement: S3 Fig — (TIF) [file pone.0303273.s004.tif]

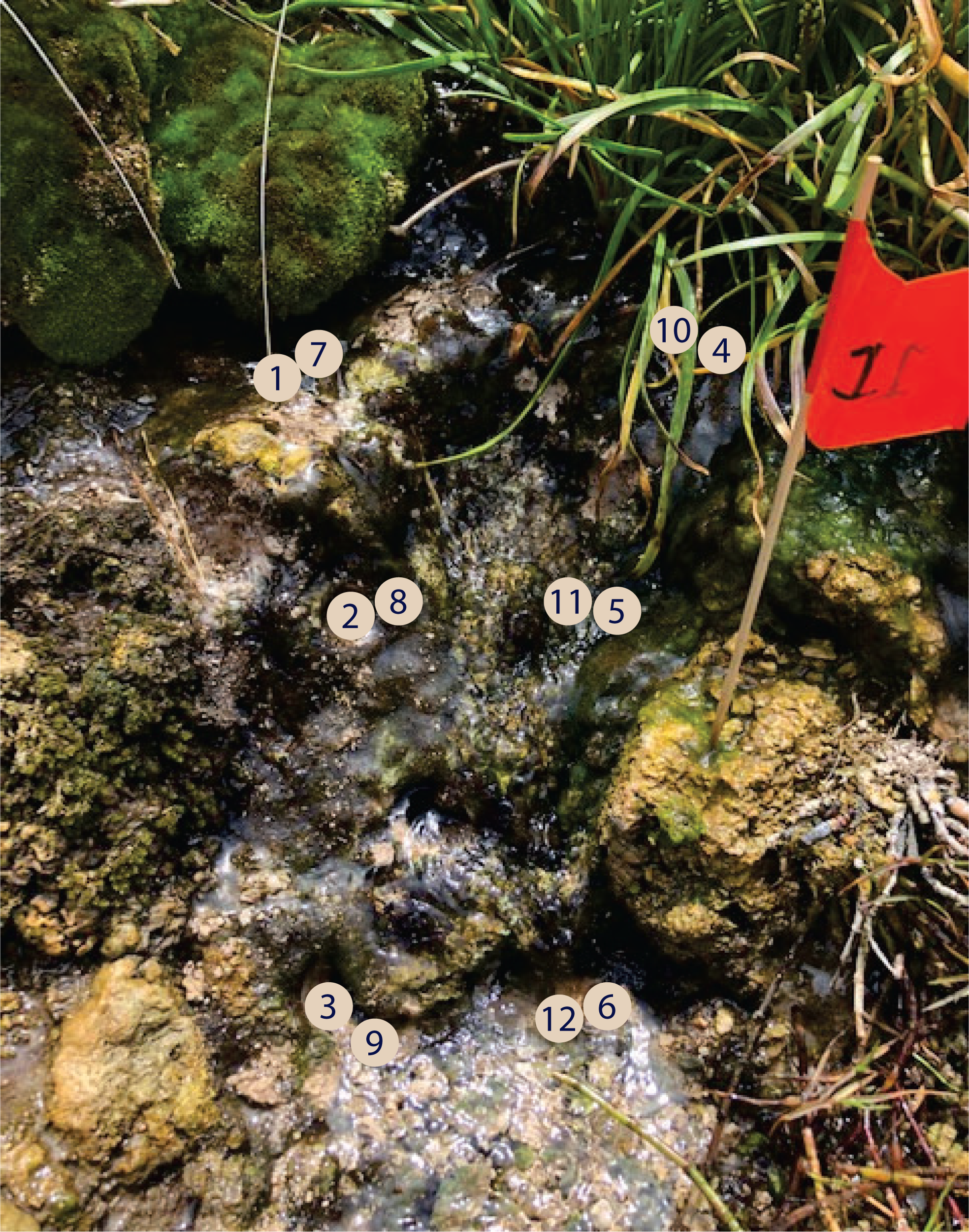

Supplement: S4 Fig — (TIF) [file pone.0303273.s005.tif]

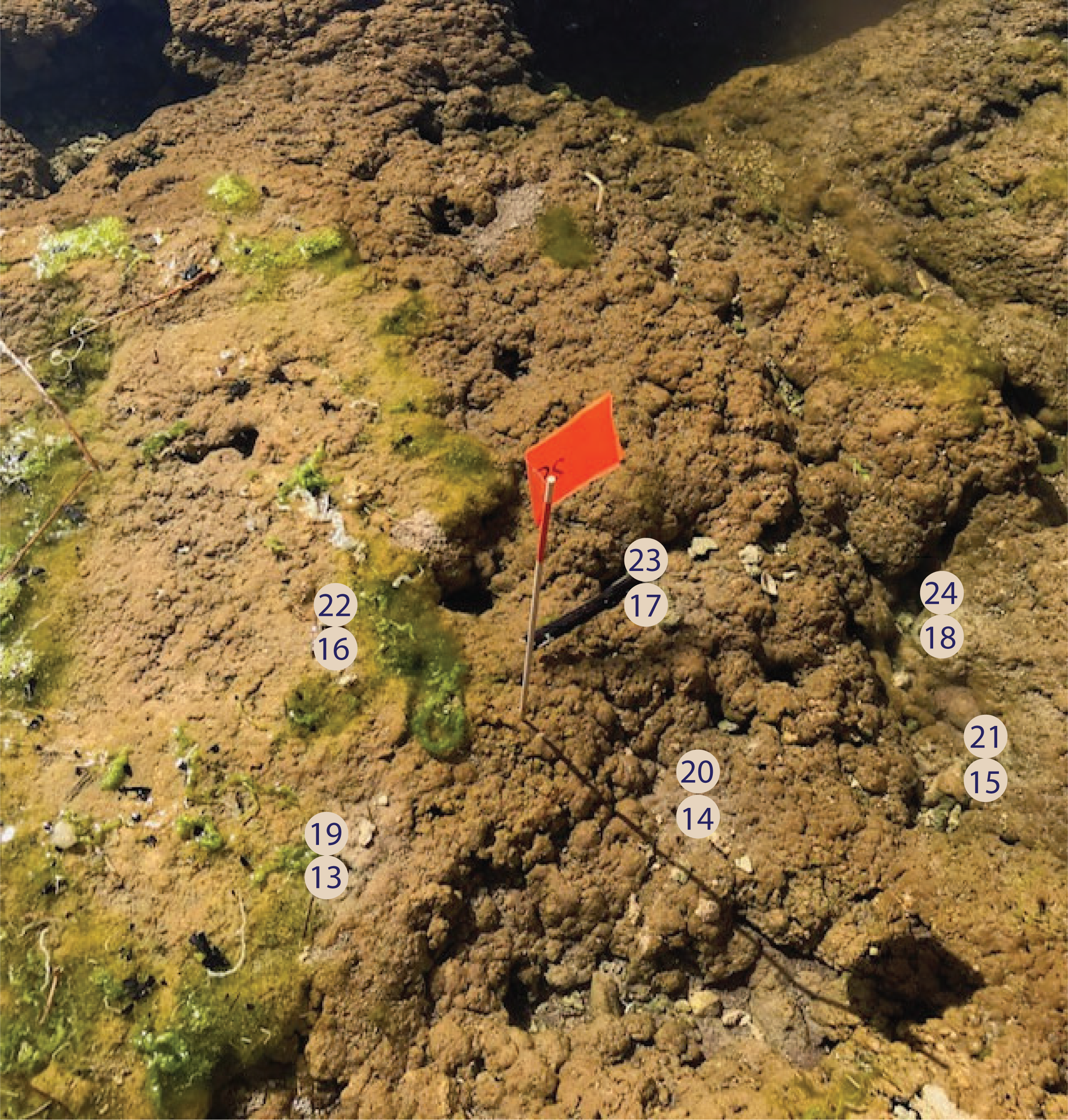

Supplement: S5 Fig — (TIF) [file pone.0303273.s006.tif]

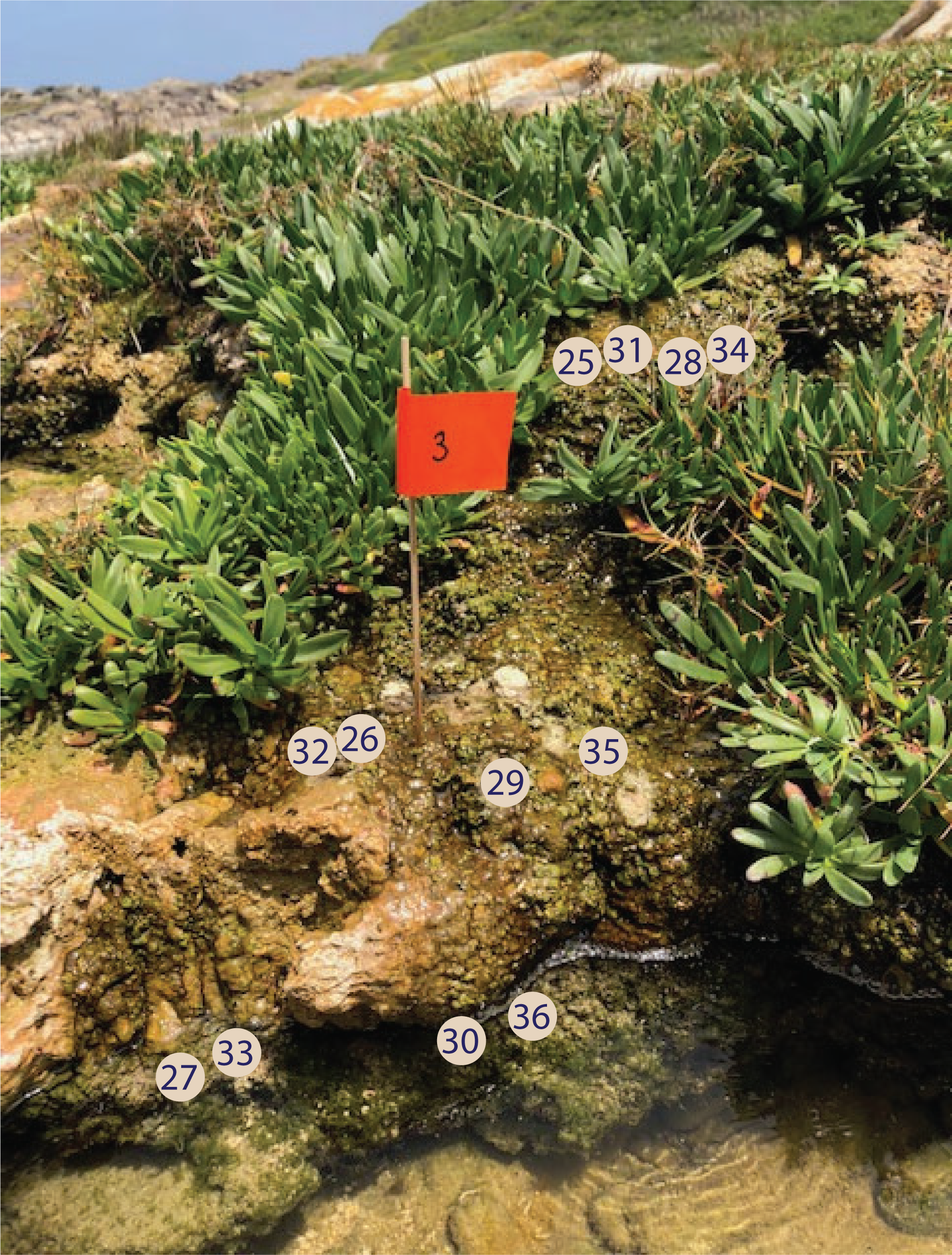

Supplement: S6 Fig — (TIF) [file pone.0303273.s007.tif]

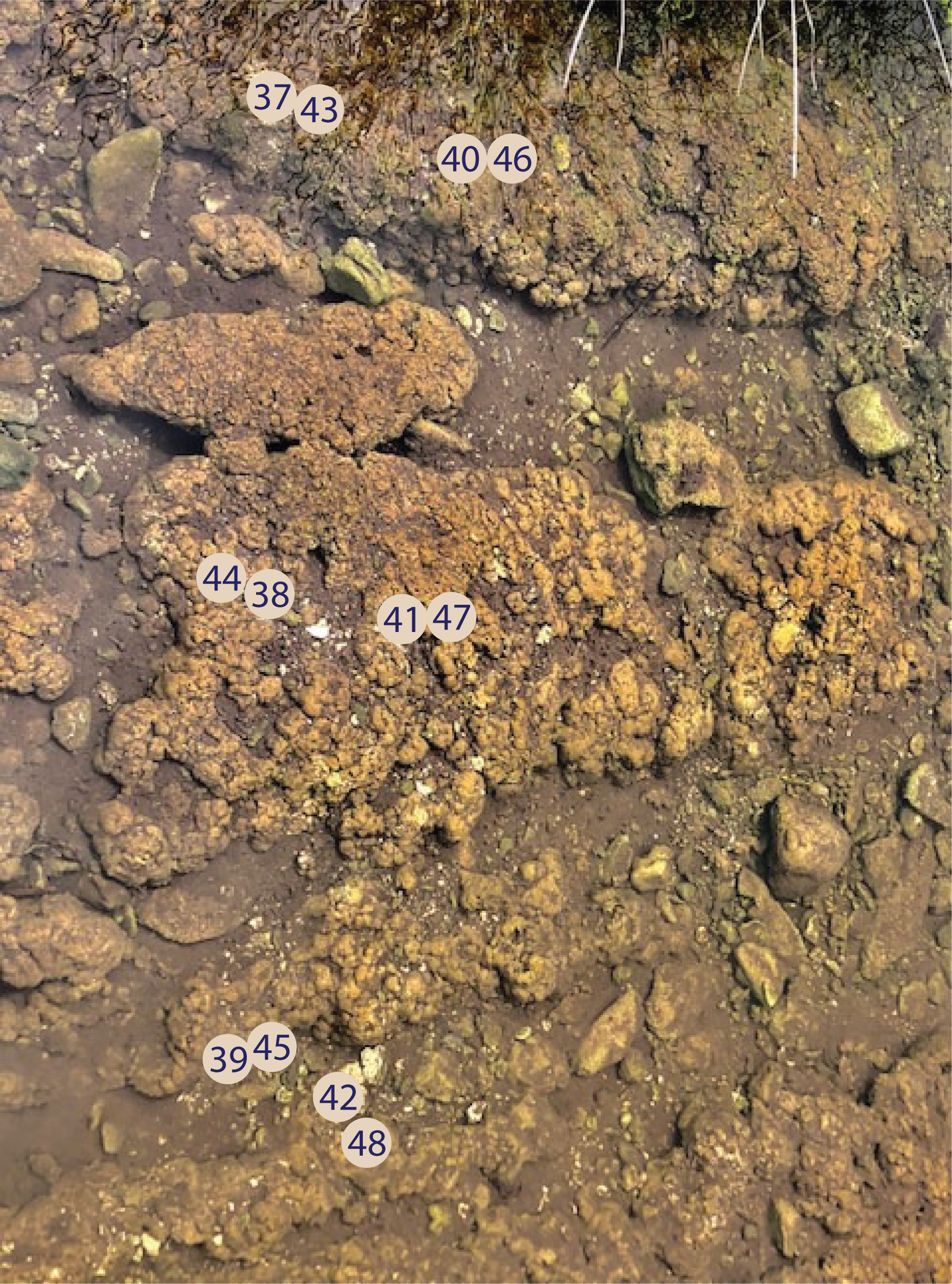

Supplement: S7 Fig — (TIF) [file pone.0303273.s008.tif]

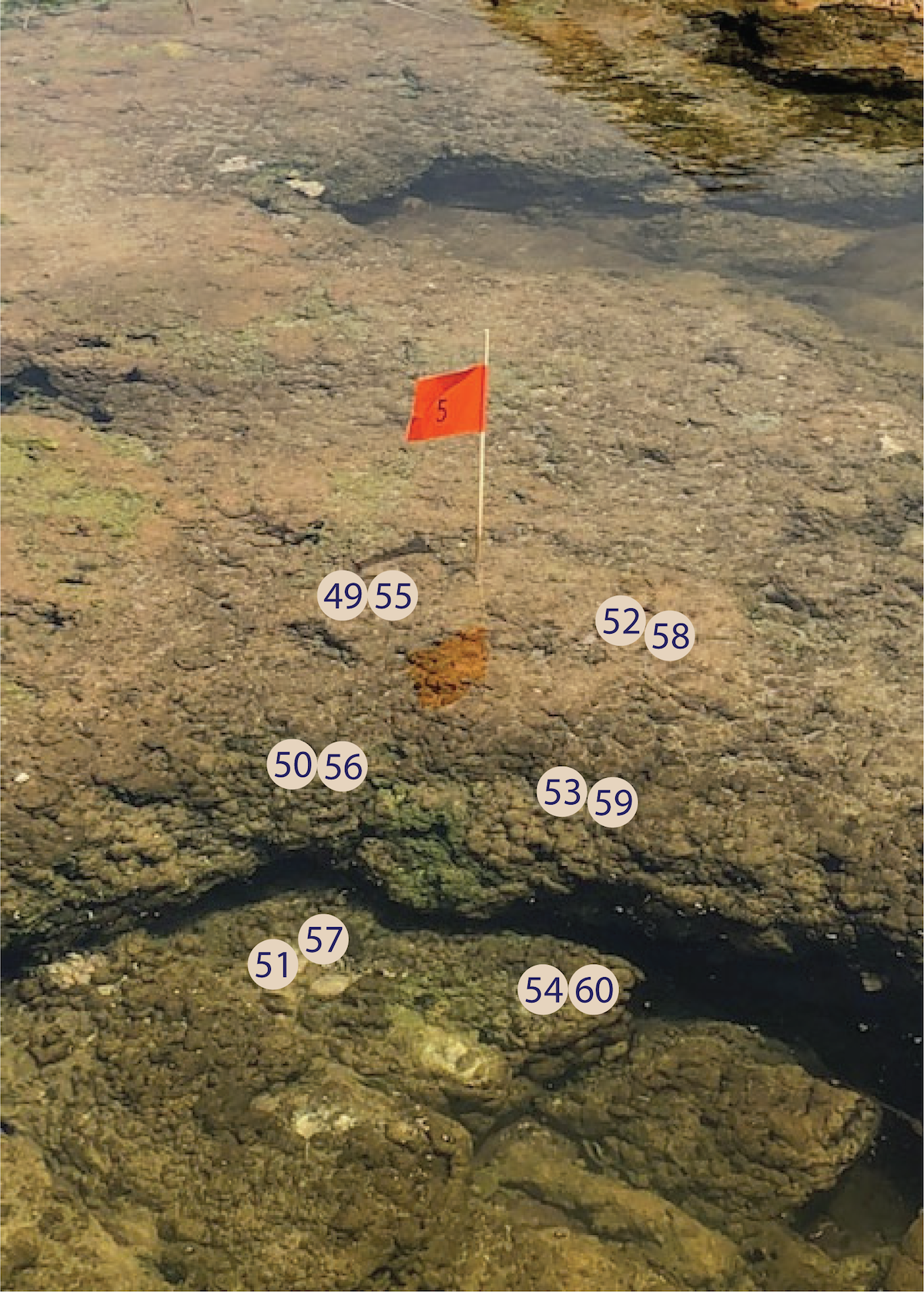

Supplement: S8 Fig — (TIF) [file pone.0303273.s009.tif]

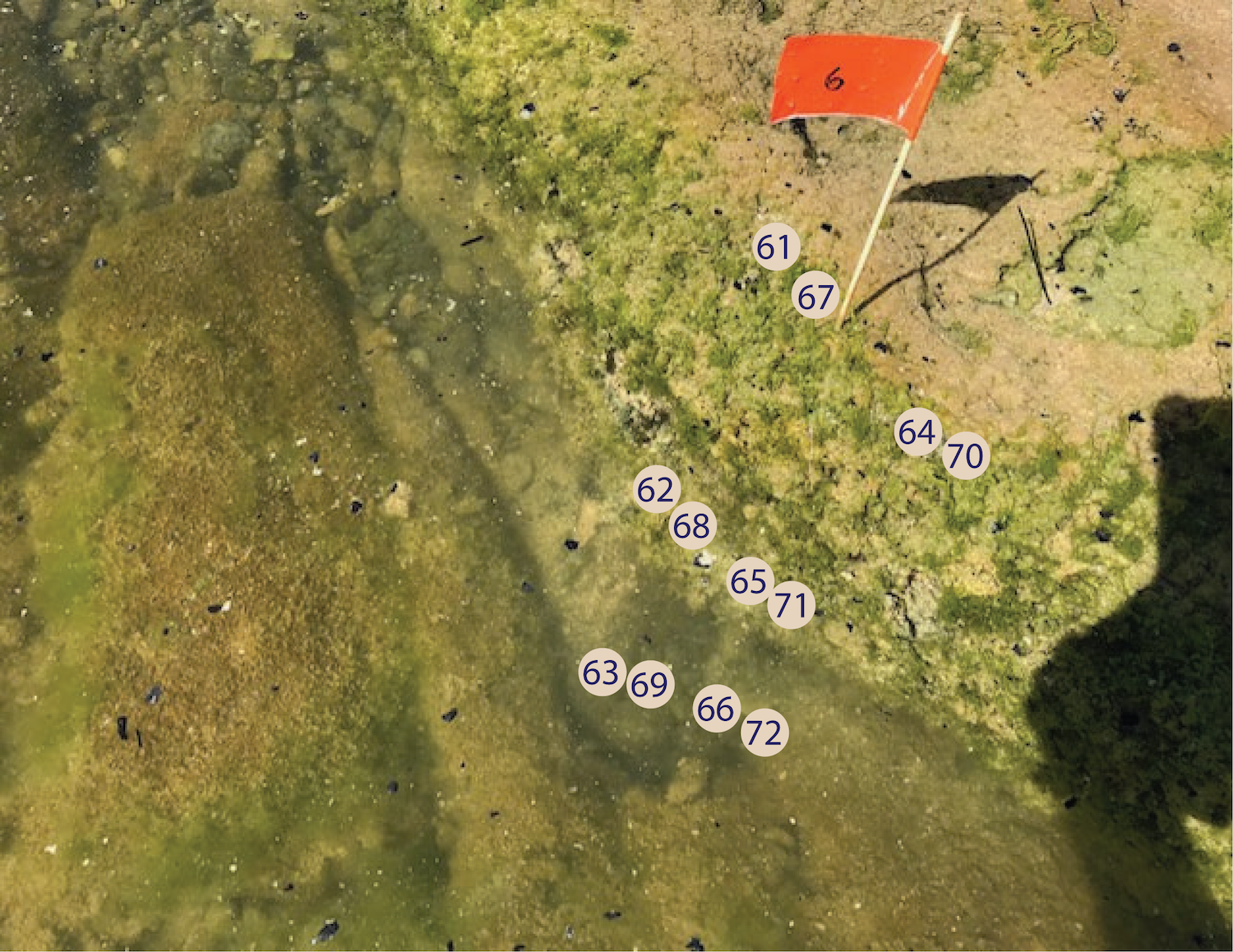

Supplement: S9 Fig — (TIF) [file pone.0303273.s010.tif]

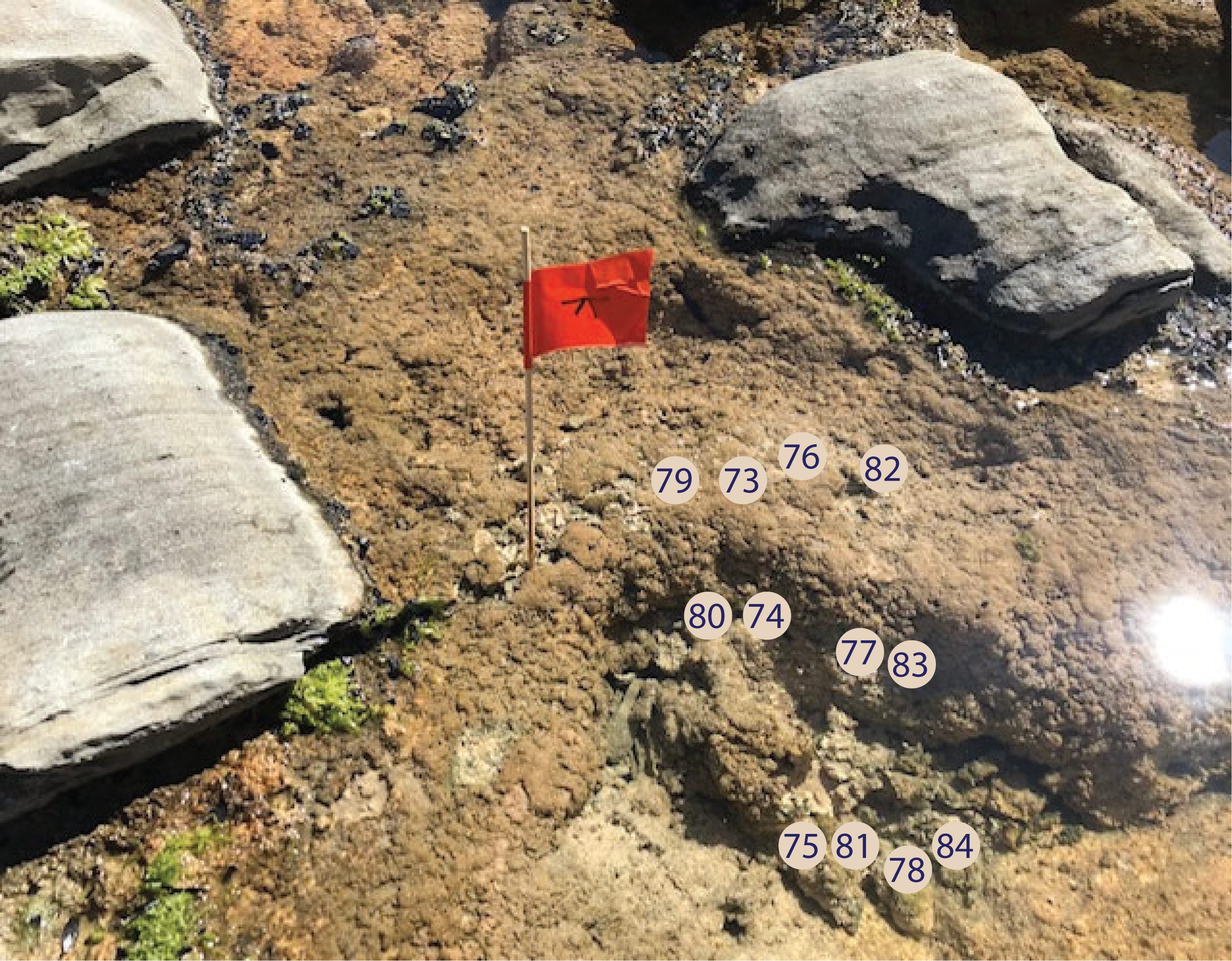

Supplement: S10 Fig — (TIF) [file pone.0303273.s011.tif]

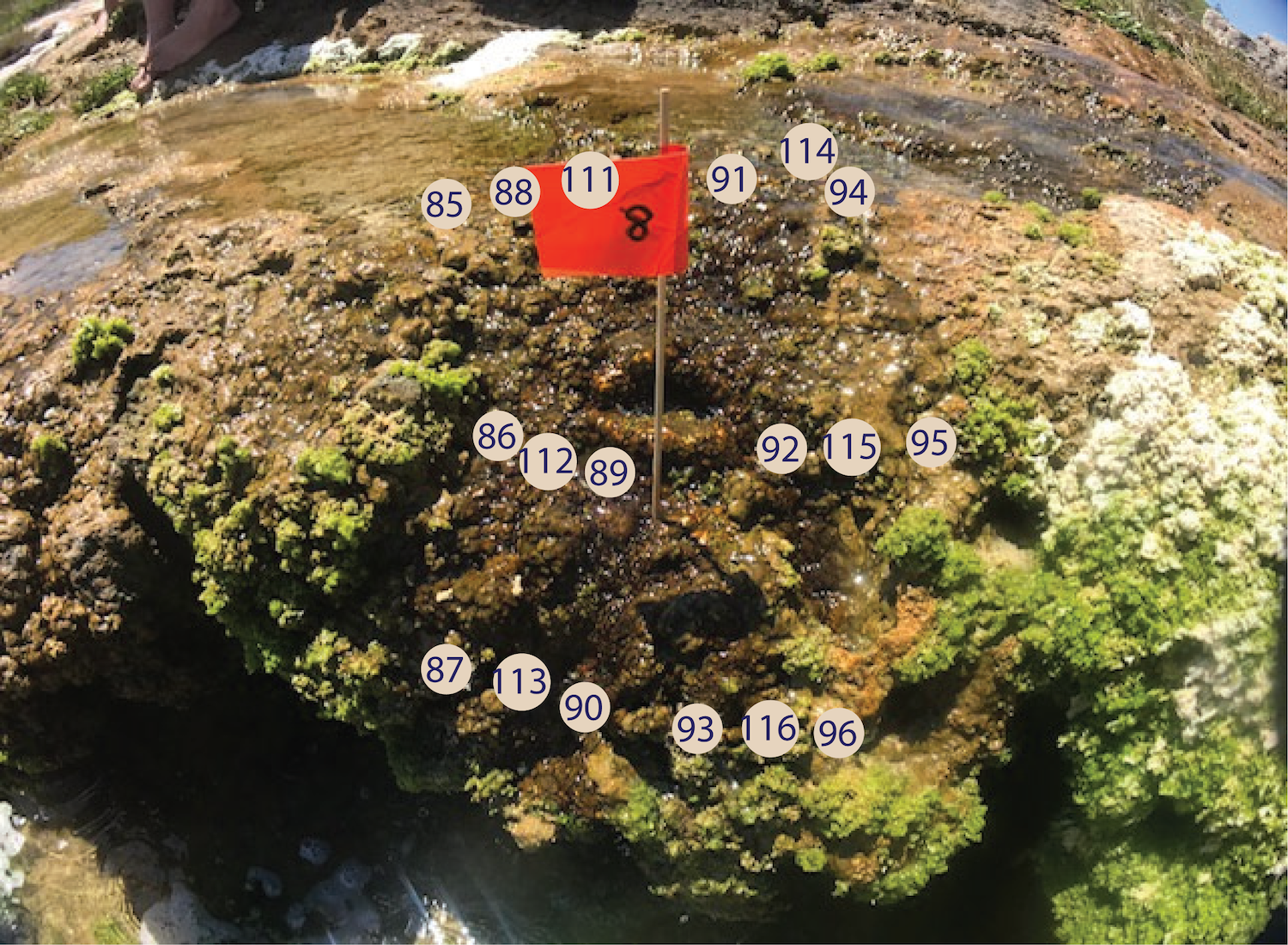

Supplement: S11 Fig — (TIF) [file pone.0303273.s012.tif]

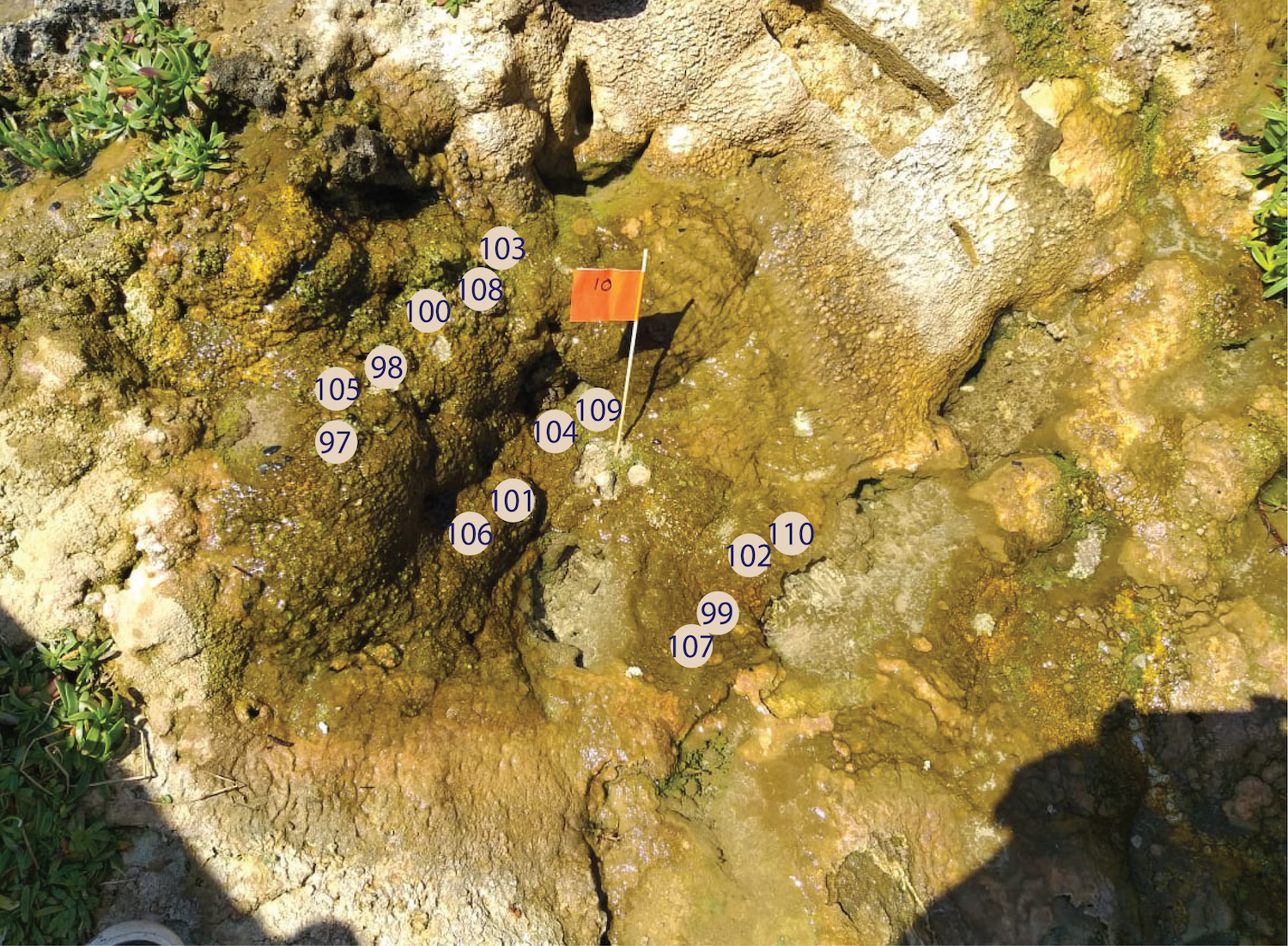

Supplement: S12 Fig — (TIF) [file pone.0303273.s013.tif]

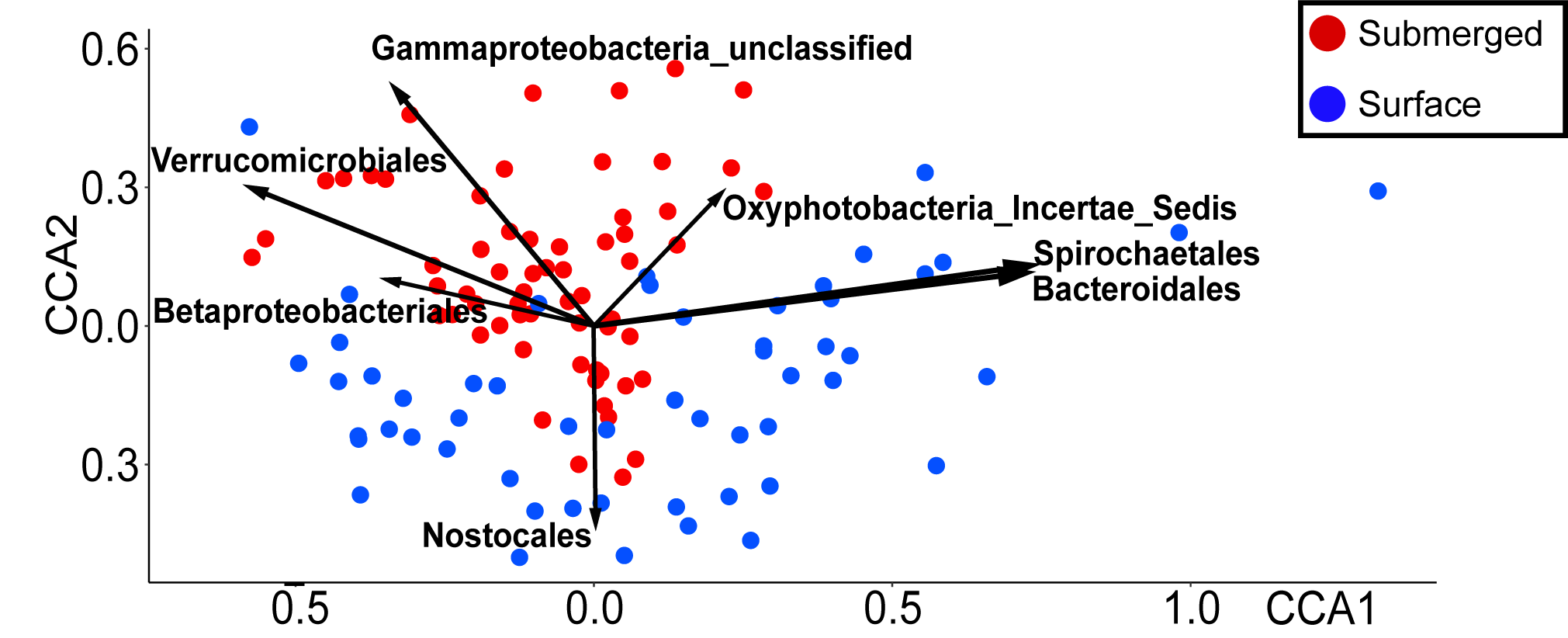

Supplement: S13 Fig — PERMANOVA, pseudo-F = 15.98, p = 0.001. Vectors indicate 16S rRNA OTUs associated with Bray–Curtis difference between substrate samples. (TIF) [file pone.0303273.s014.tif]

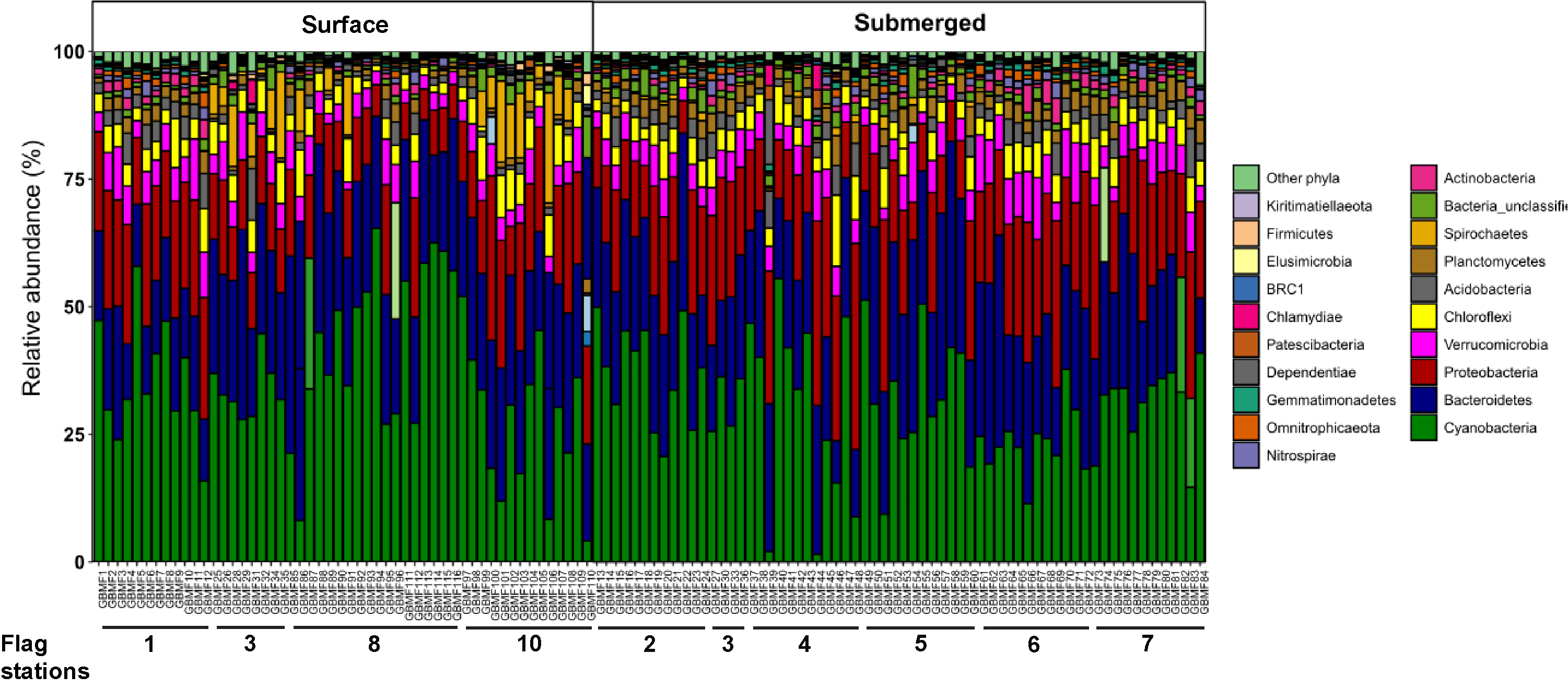

Supplement: S14 Fig — All samples are dominated by Cyanobacteria, Bacteroidetes, and Proteobacteria. (TIF) [file pone.0303273.s015.tif]

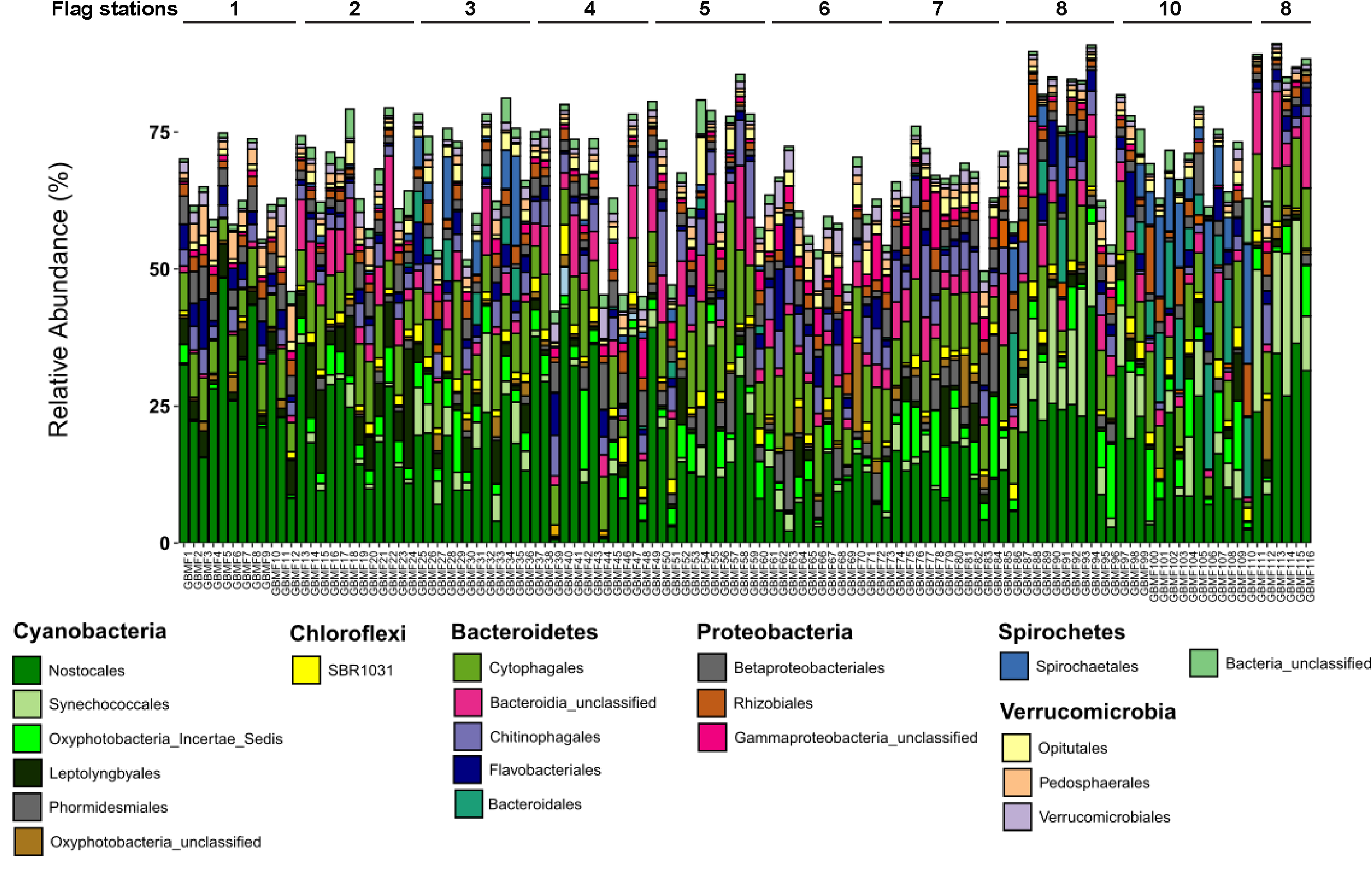

Supplement: S15 Fig — 20 OTUs accounted for between 50–80% of all the reads across samples, shown here classified to the rank of Order. (TIF) [file pone.0303273.s016.tif]

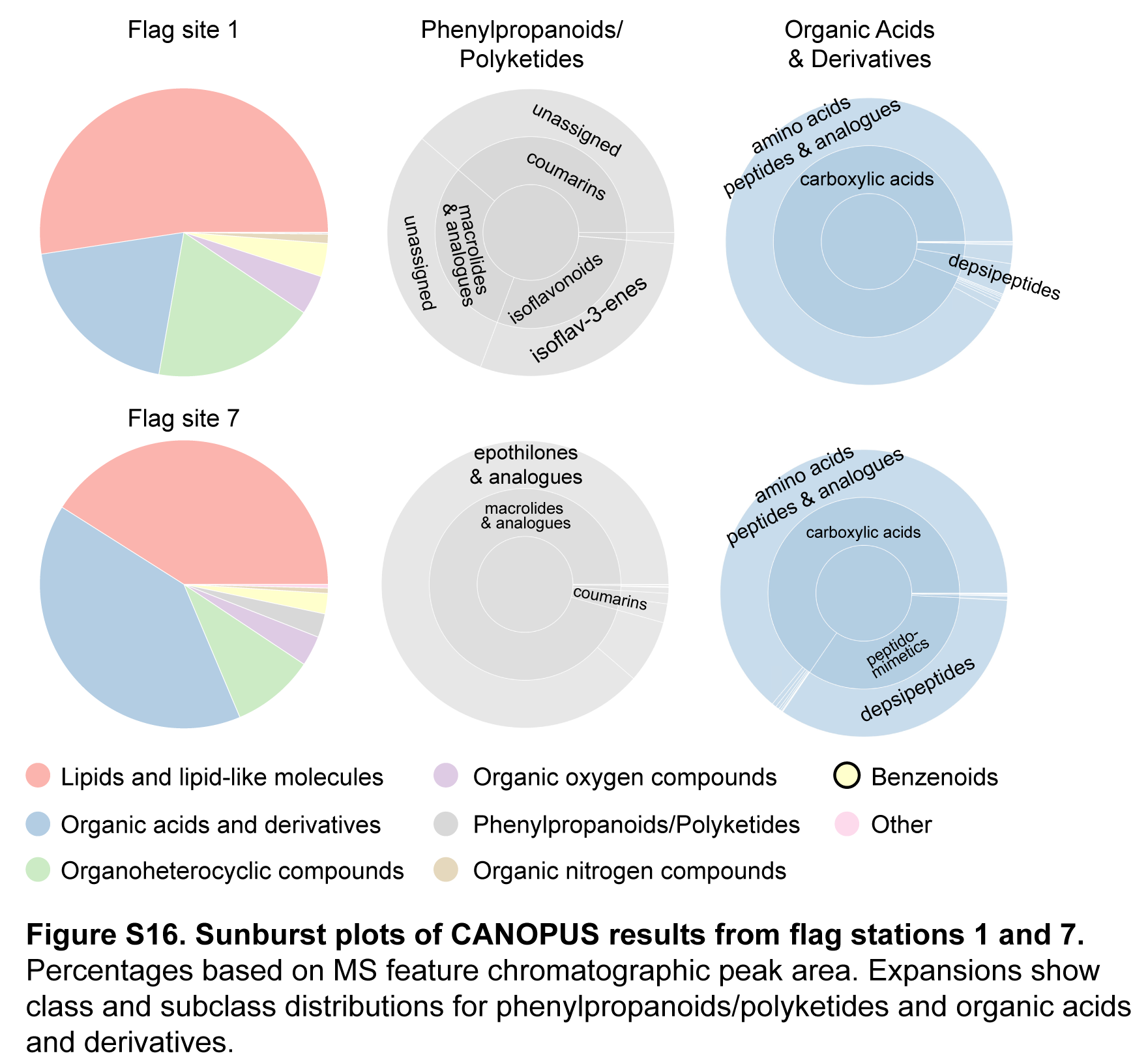

Supplement: S16 Fig — Percentages based on MS feature chromatographic peak area. Expansions show class and subclass distributions for phenylpropanoids/polyketides and organic acids and derivatives. (TIF) [file pone.0303273.s017.tif]

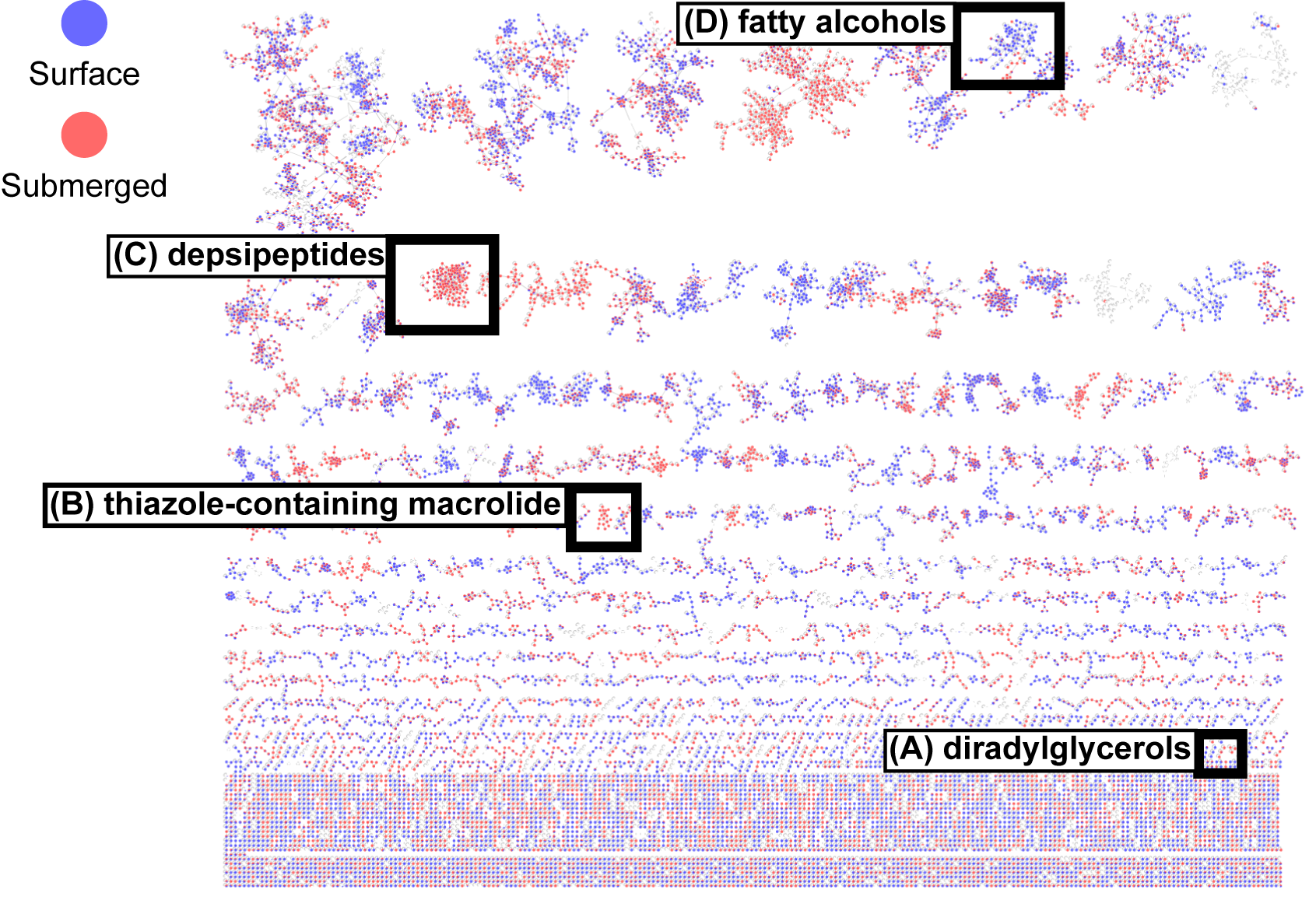

Supplement: S17 Fig — Subnetworks containing driver MS features are labeled with the structural class predicted by CANOPUS (A-D). (TIF) [file pone.0303273.s018.tif]

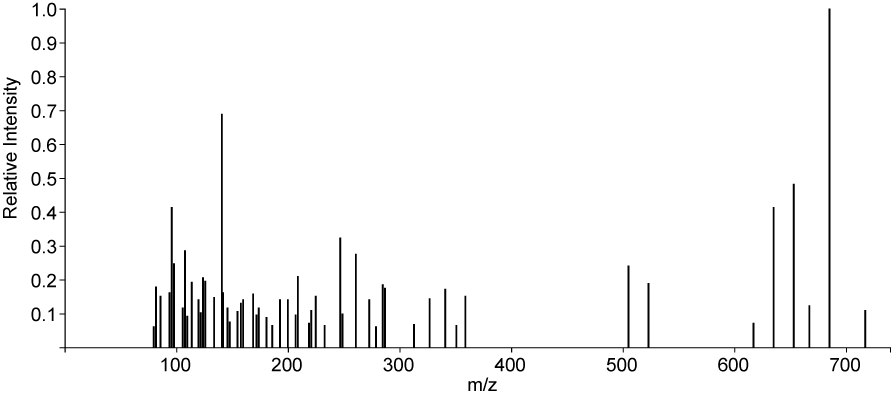

Supplement: S18 Fig — Annotated as epothilone by CANOPUS. (TIF) [file pone.0303273.s019.tif]

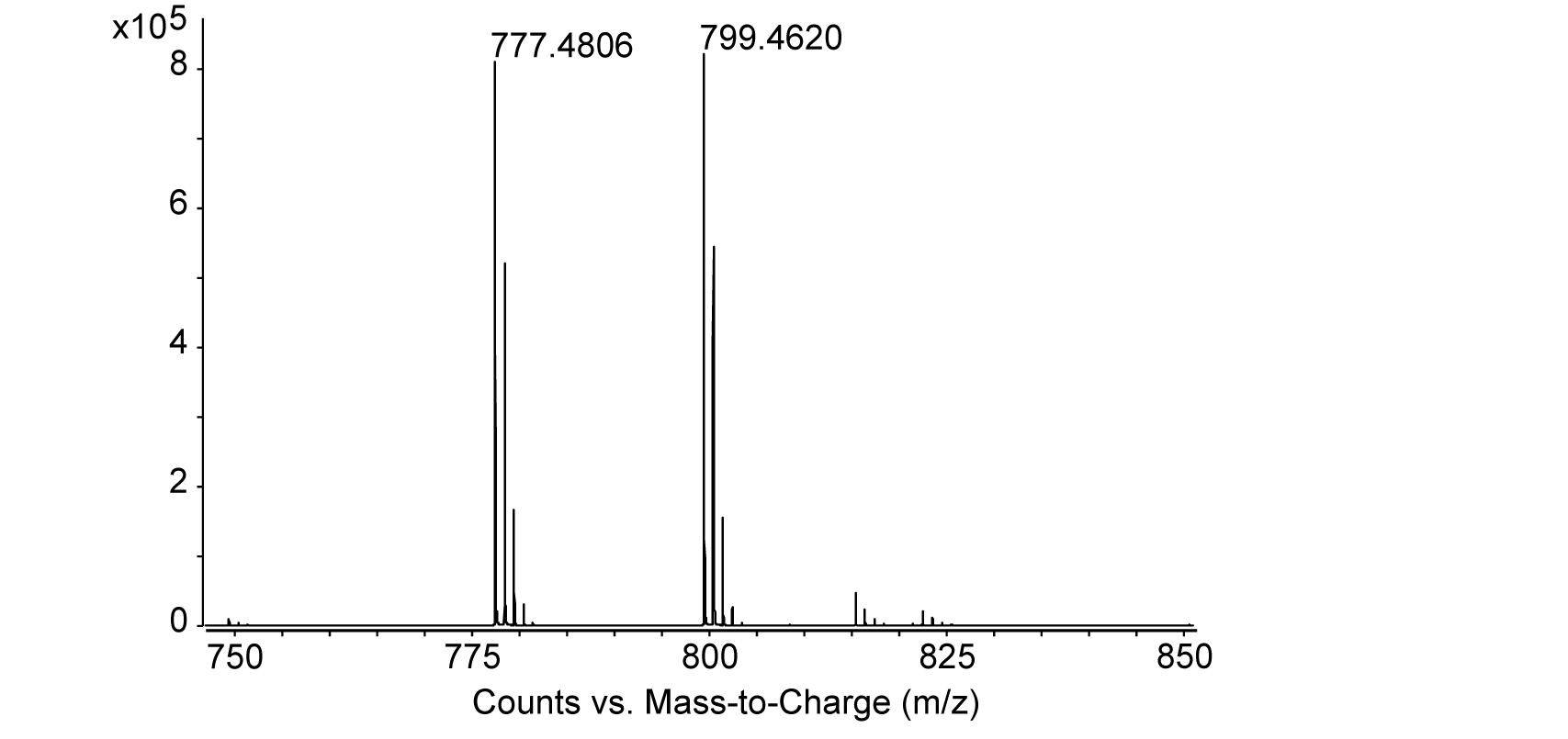

Supplement: S19 Fig — m/z 777.4806, MS feature 2198, [M+H]+; m/z 799.4620, MS feature 880, [M+Na]+. (TIF) [file pone.0303273.s020.tif]

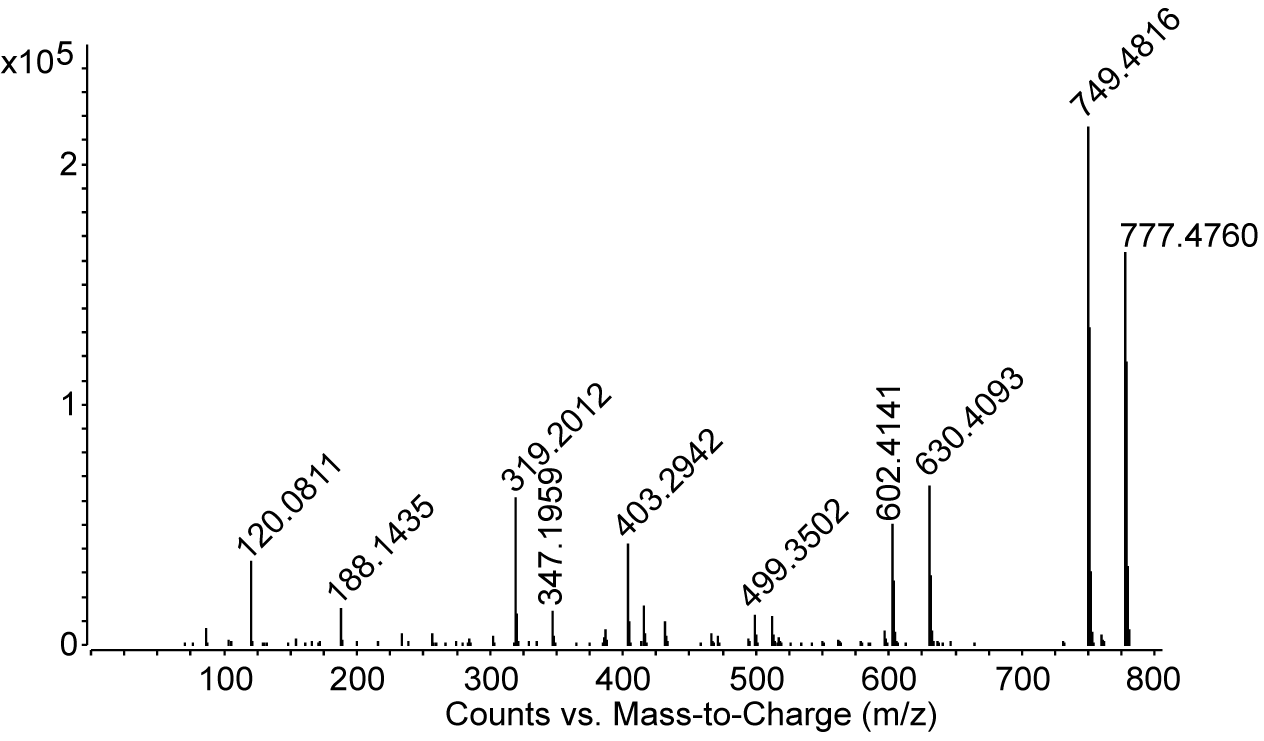

Supplement: S20 Fig — MS feature 2198. (TIF) [file pone.0303273.s021.tif]

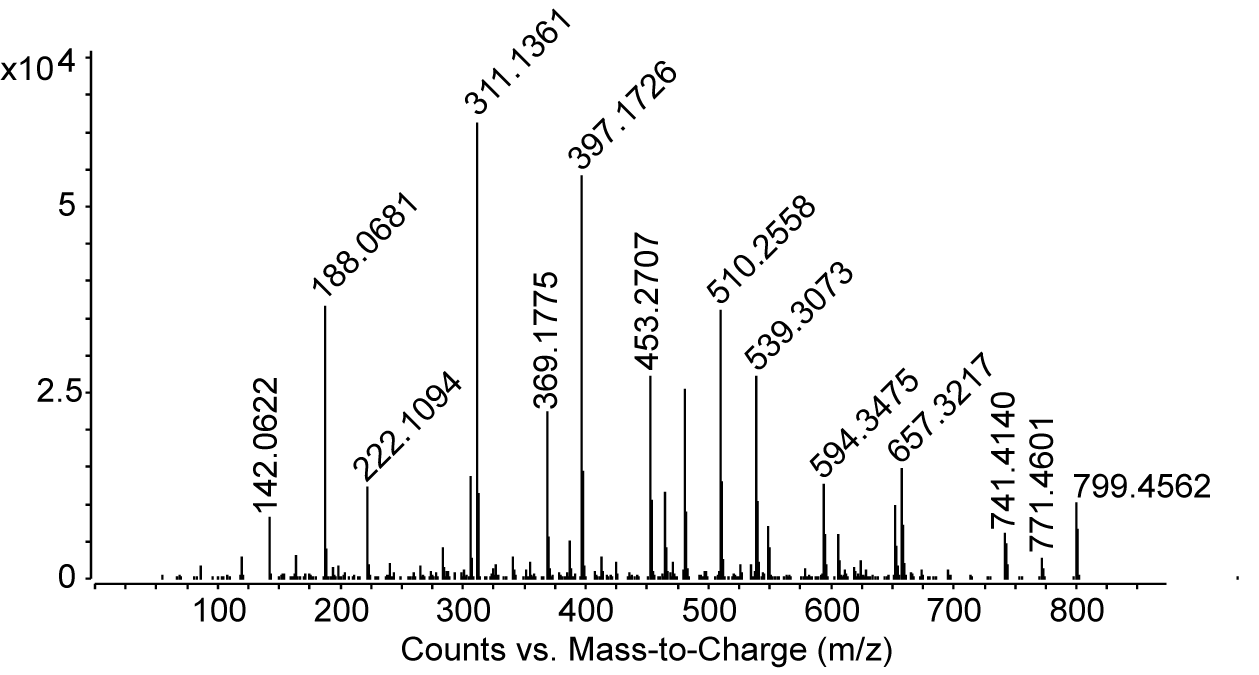

Supplement: S21 Fig — MS feature 880. (TIF) [file pone.0303273.s022.tif]

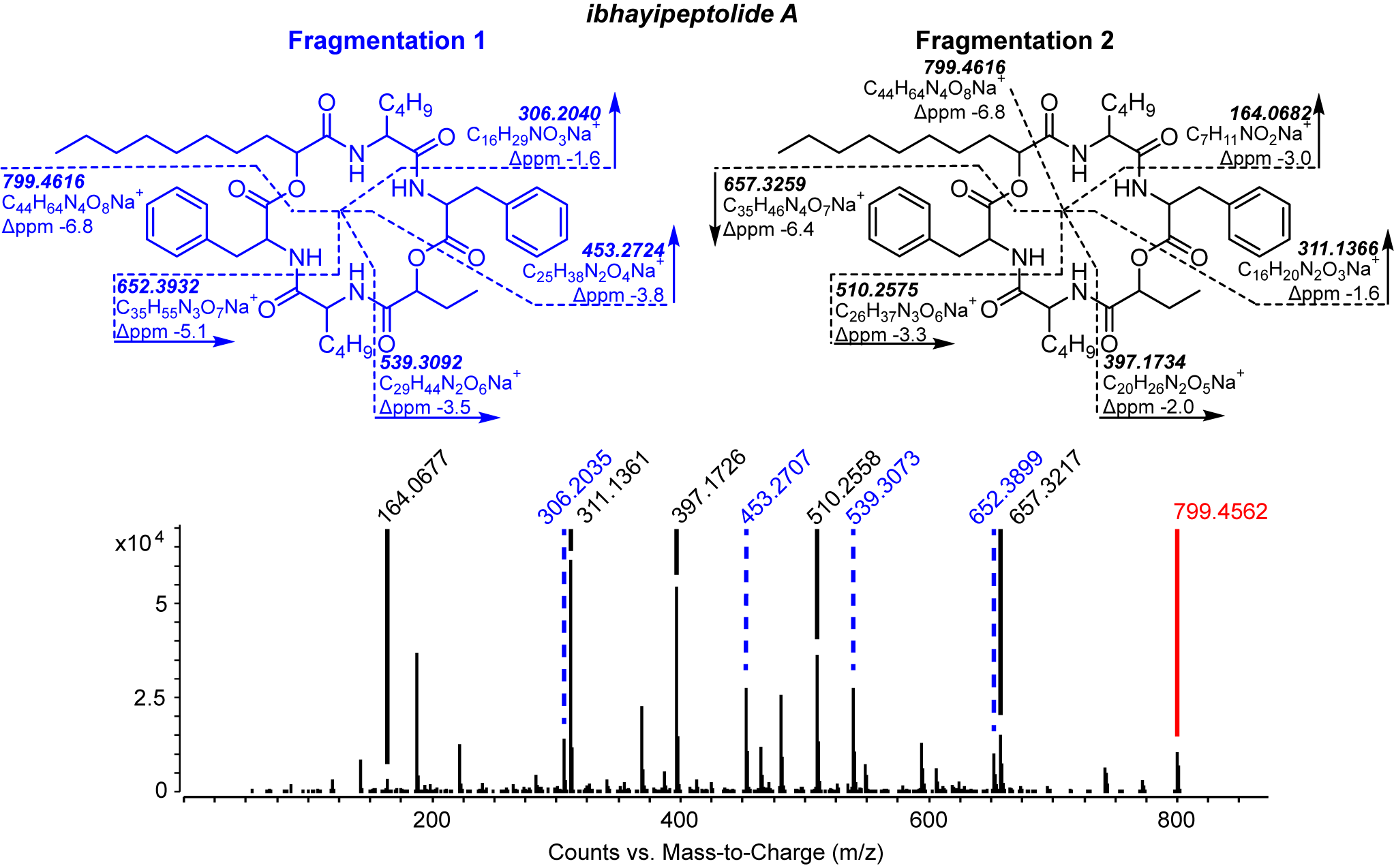

Supplement: S22 Fig — Top) Predicted fragmentation with theoretical mass and ppm error of fragment ions. Bottom) MS2 spectra ([M+Na]+, 60eV) labeled with HR mass (fragmentation 1: blue dotted lines, fragmentation 2: black solid lines). (TIF) [file pone.0303273.s023.tif]

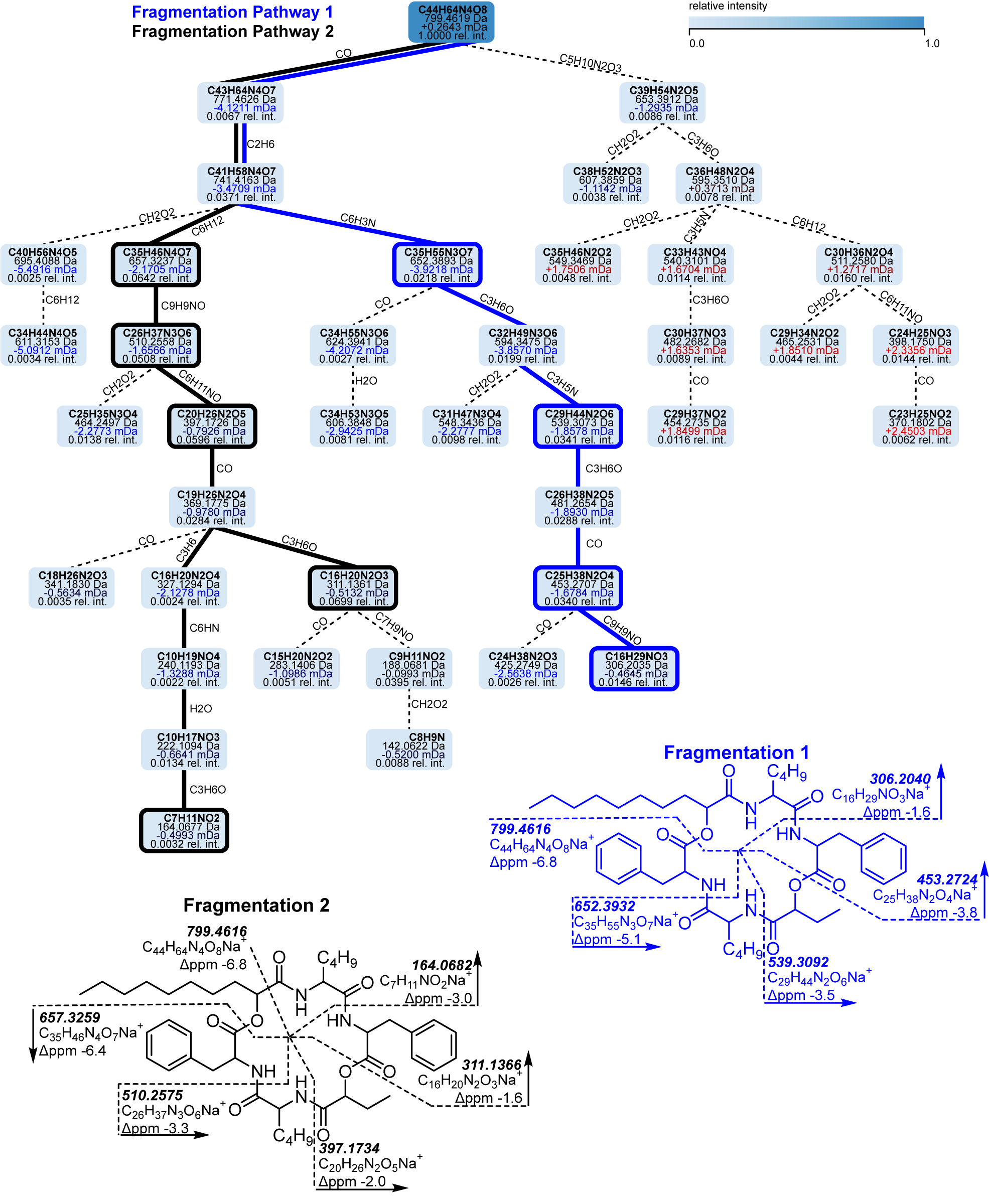

Supplement: S23 Fig — (TIF) [file pone.0303273.s024.tif]

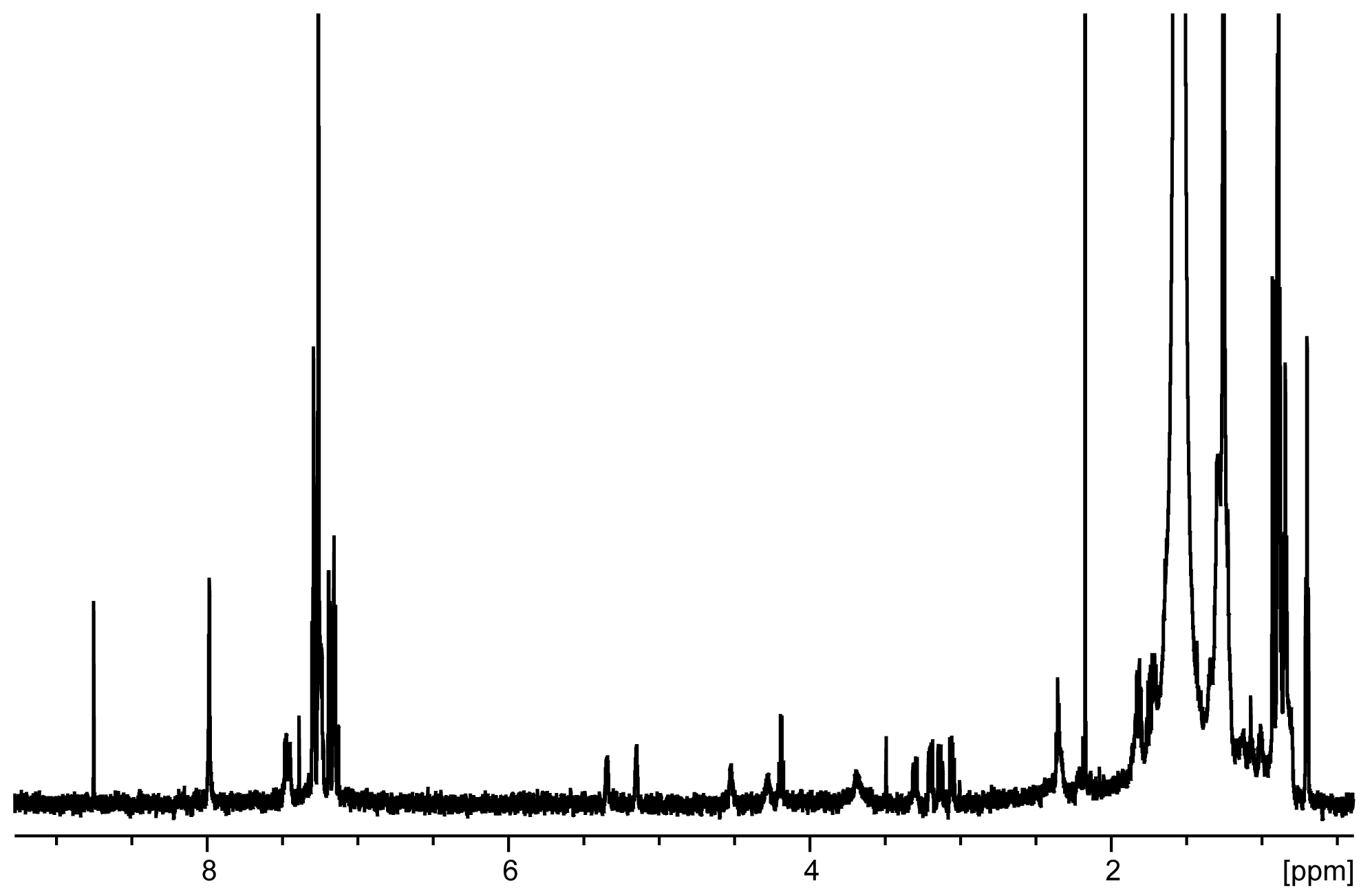

Supplement: S24 Fig — CDCl3, 800 MHz, TCI, 3 mm tube. (TIF) [file pone.0303273.s025.tif]

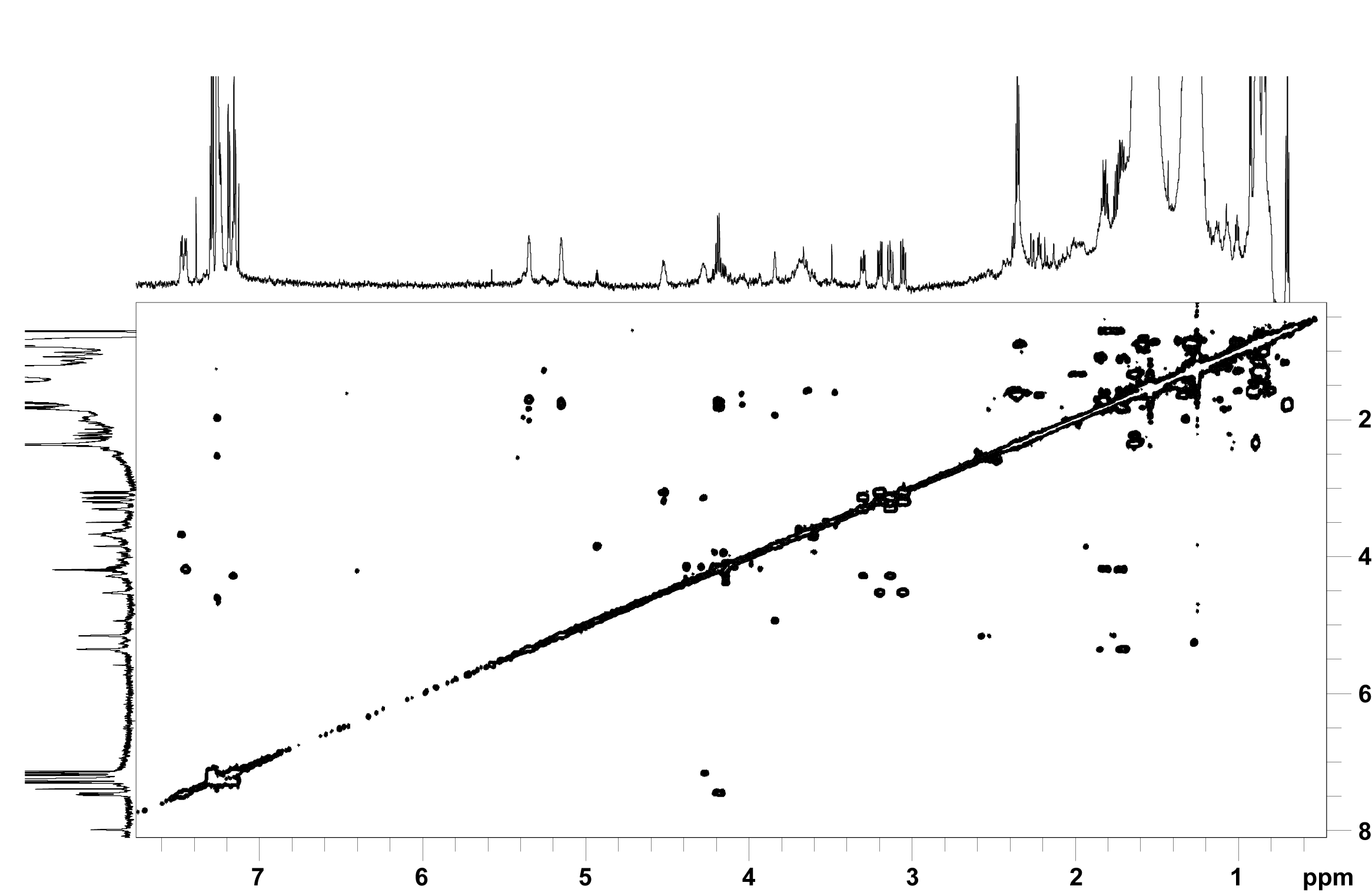

Supplement: S25 Fig — CDCl3, 800 MHz, TCI, 3 mm tube. (TIF) [file pone.0303273.s026.tif]

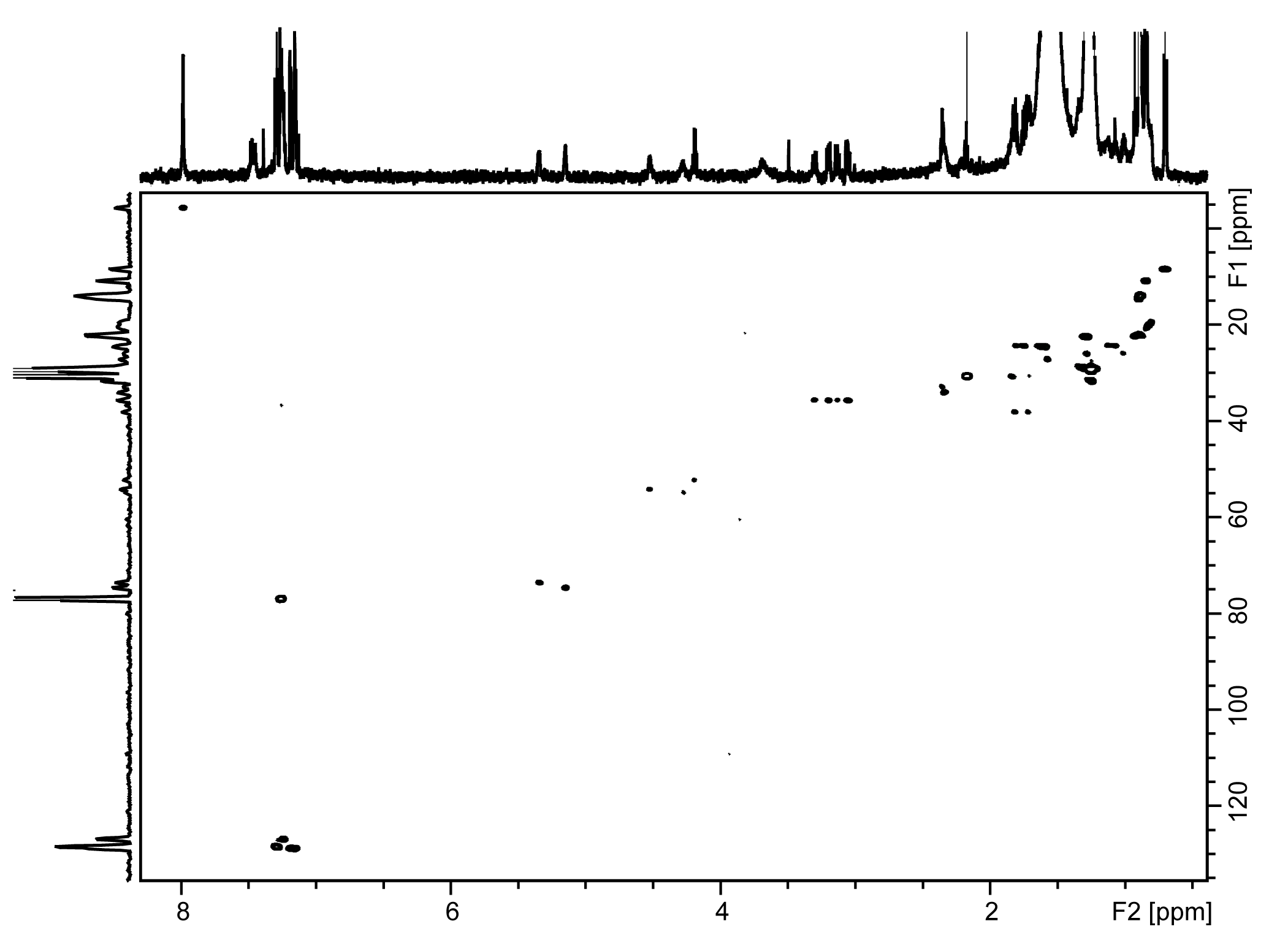

Supplement: S26 Fig — CDCl3, 800 MHz, TCI, 3 mm tube. (TIF) [file pone.0303273.s027.tif]

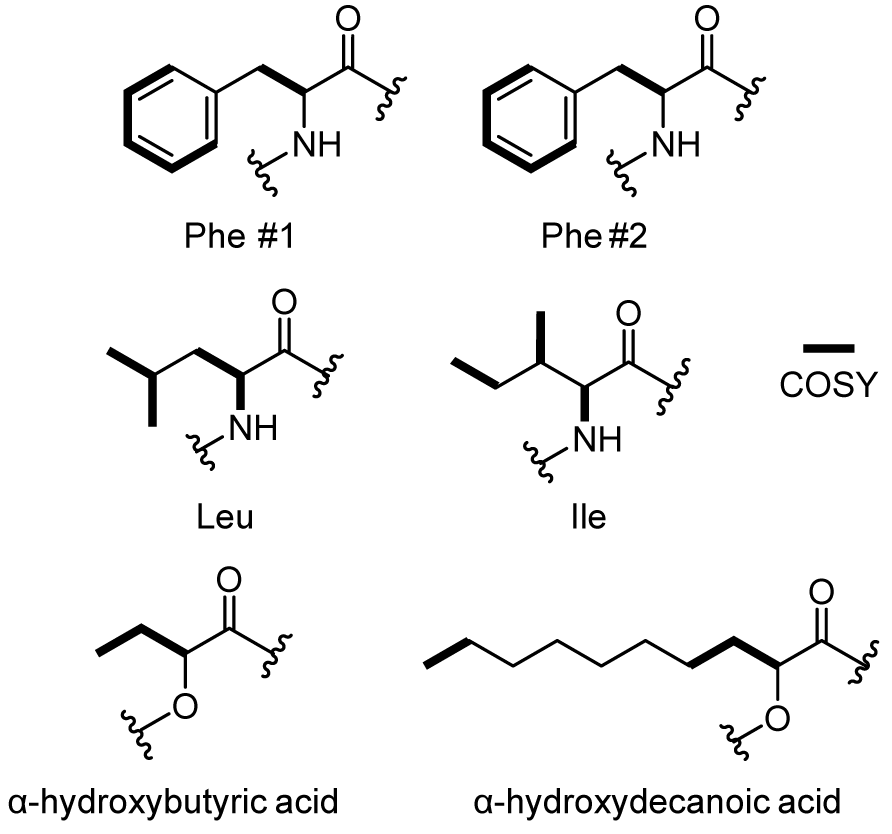

Supplement: S27 Fig — (TIF) [file pone.0303273.s028.tif]

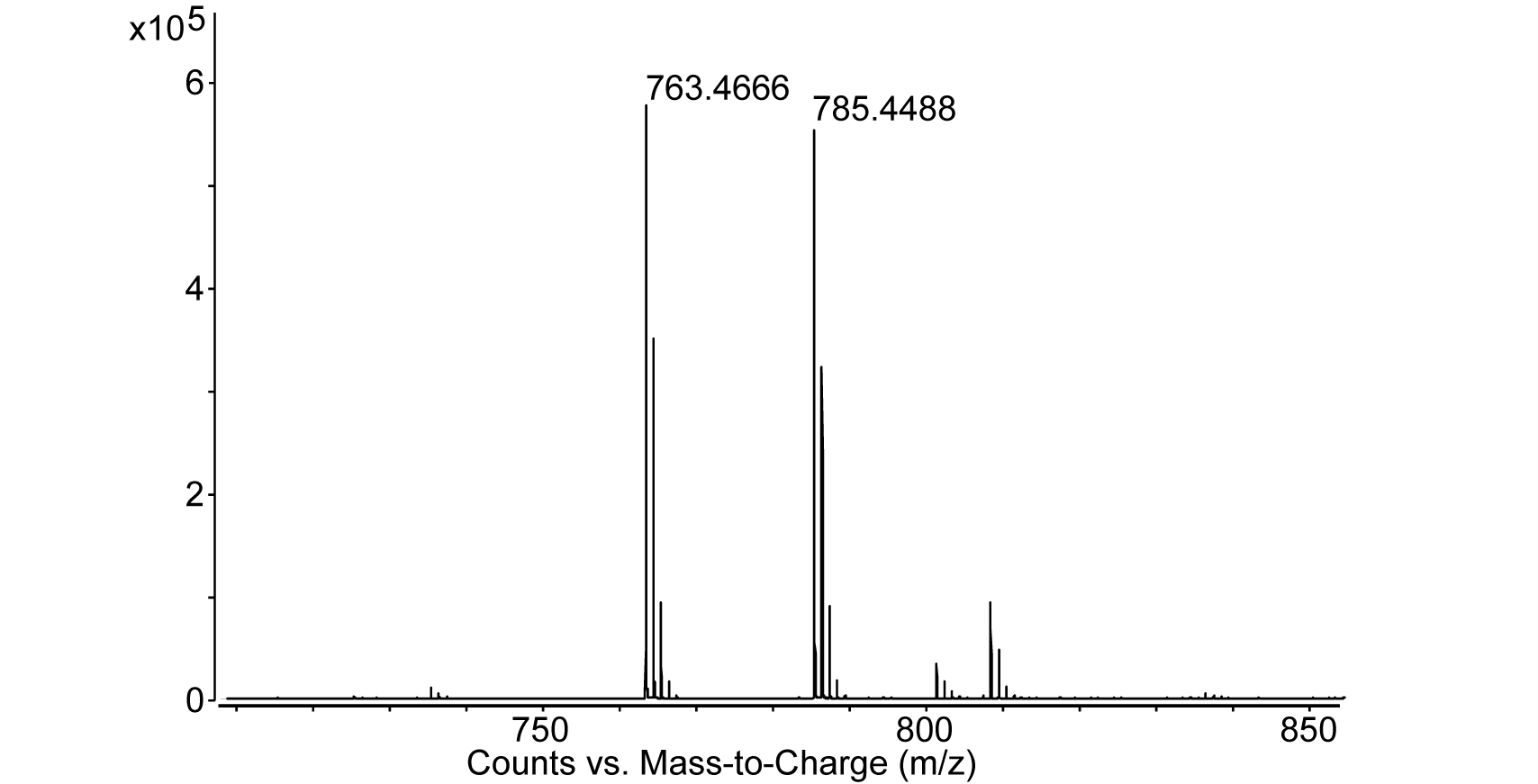

Supplement: S28 Fig — m/z 763.4666, MS feature 8186, [M+H]+; m/z 785.4488, MS feature 2363, [M+Na]+. (TIF) [file pone.0303273.s029.tif]

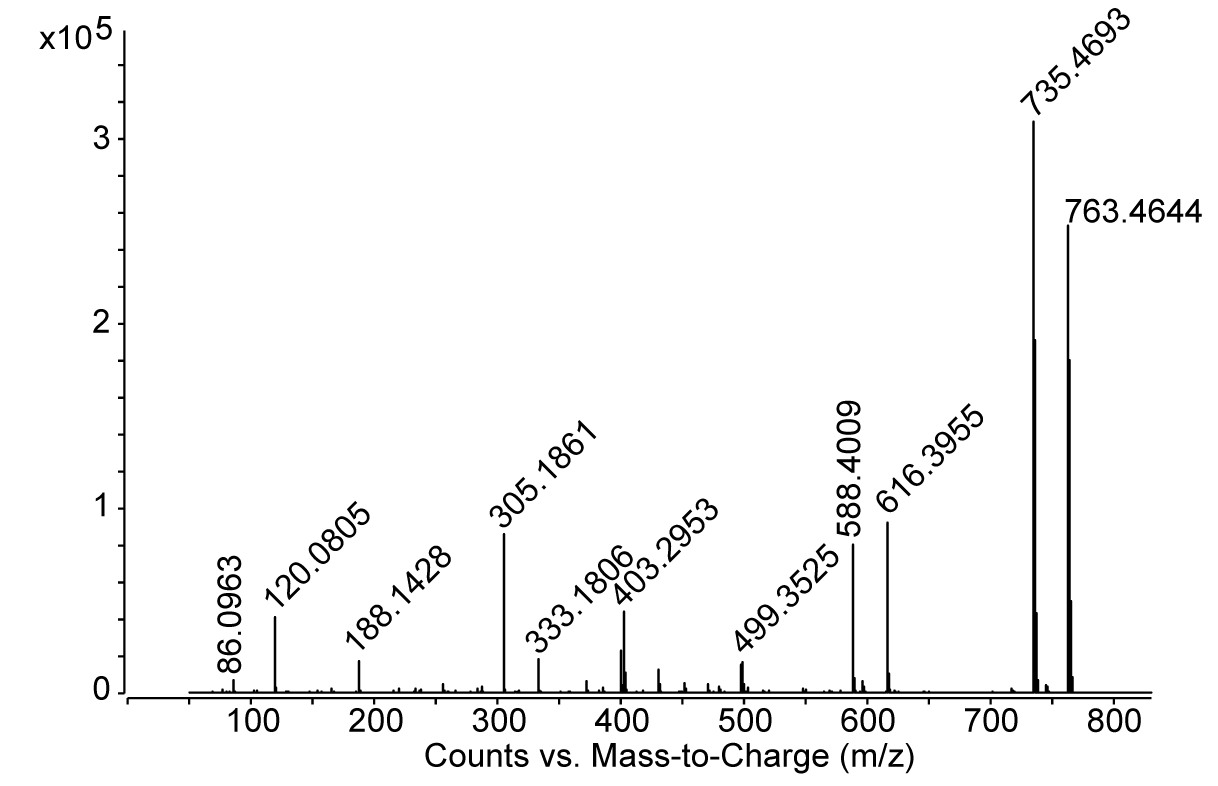

Supplement: S29 Fig — (TIF) [file pone.0303273.s030.tif]

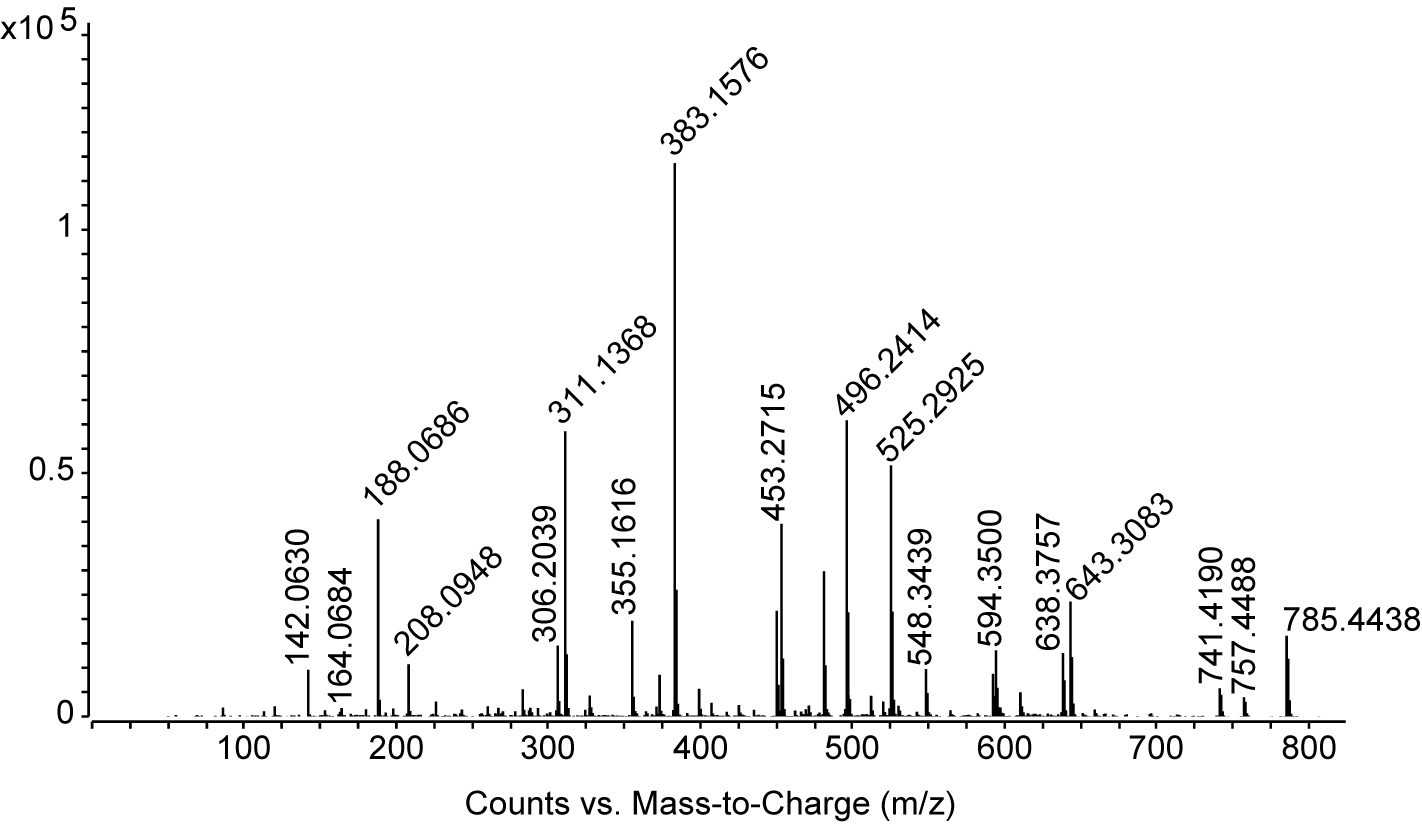

Supplement: S30 Fig — (TIF) [file pone.0303273.s031.tif]

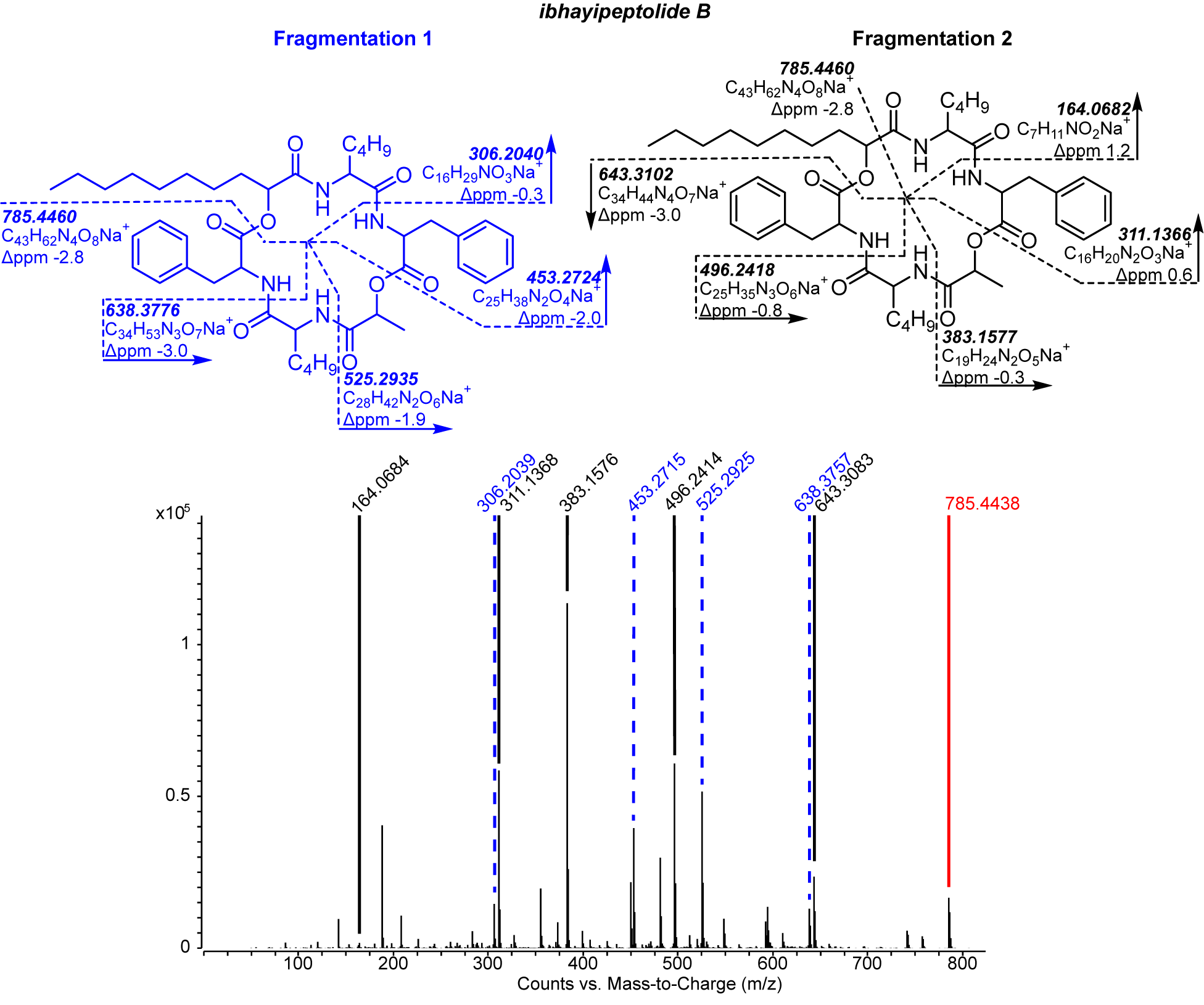

Supplement: S31 Fig — Predicted fragmentation with theoretical mass and ppm error of fragment ions (Top). MS2 spectra ([M+Na]+, 60eV) labelled with HR mass (Bottom; fragmentation 1: blue dotted lines, fragmentation 2: black solid lines). (TIF) [file pone.0303273.s032.tif]

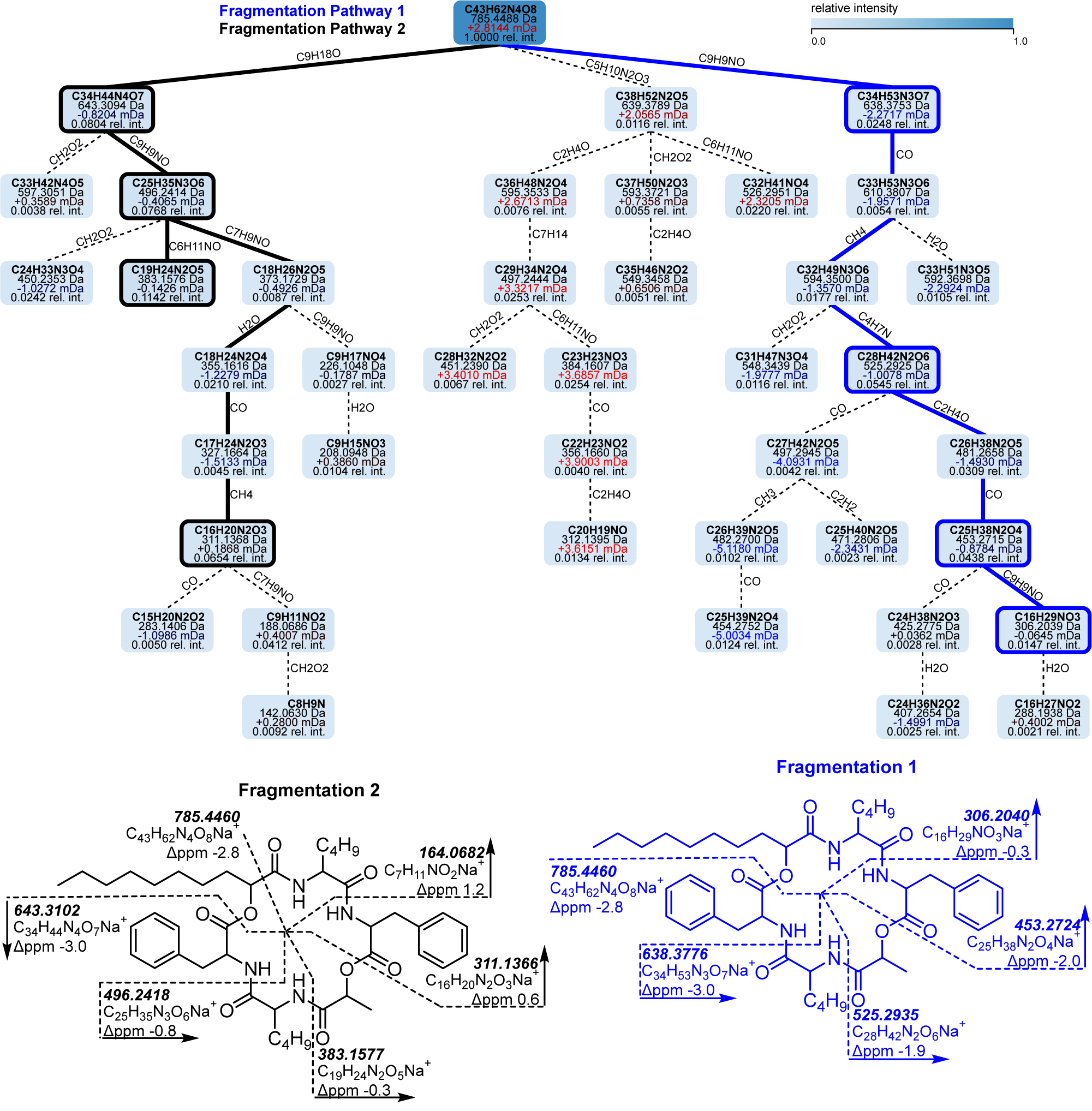

Supplement: S32 Fig — (TIF) [file pone.0303273.s033.tif]

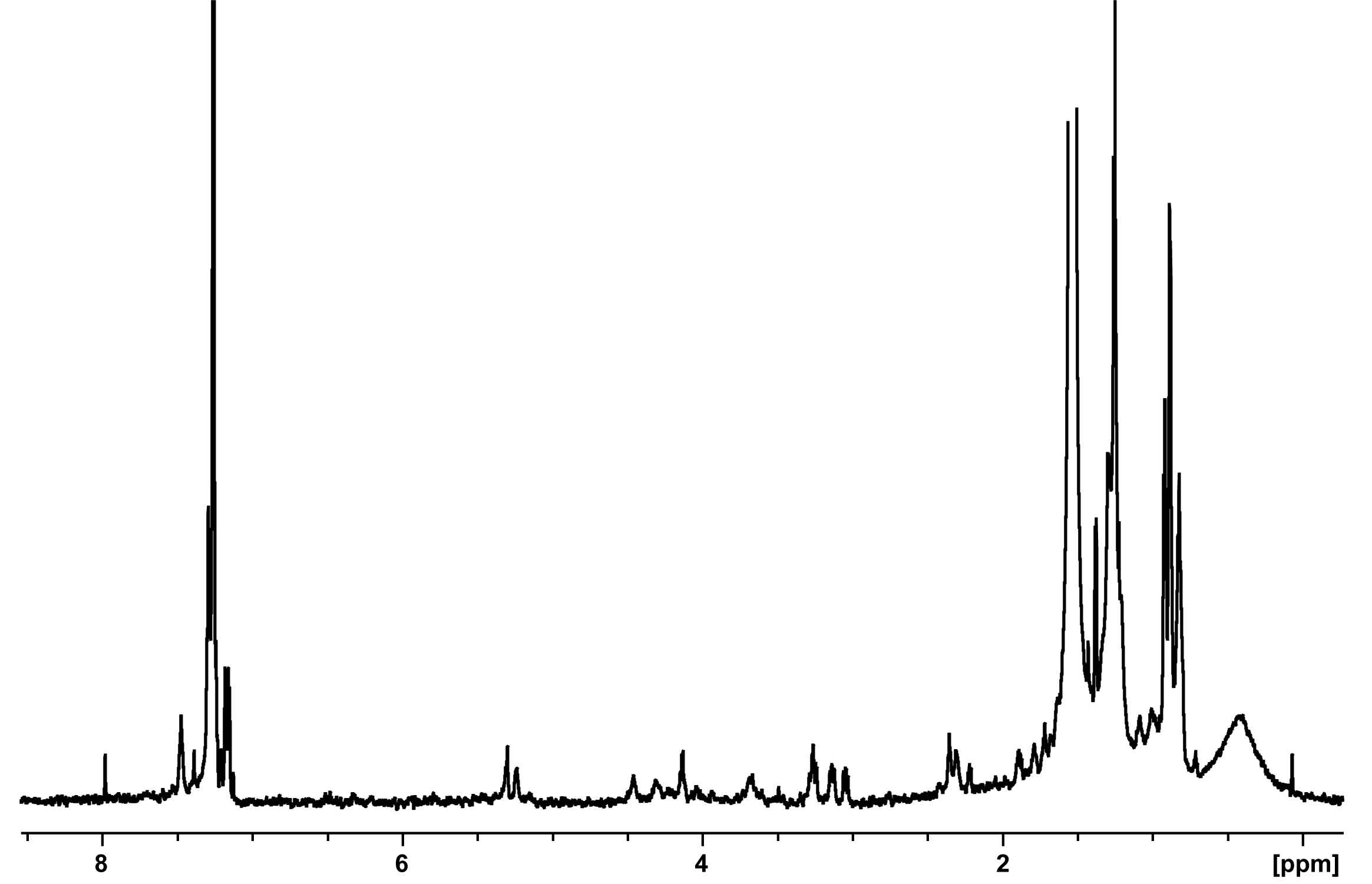

Supplement: S33 Fig — CDCl3, 800 MHz, TCI, 3 mm tube. (TIF) [file pone.0303273.s034.tif]

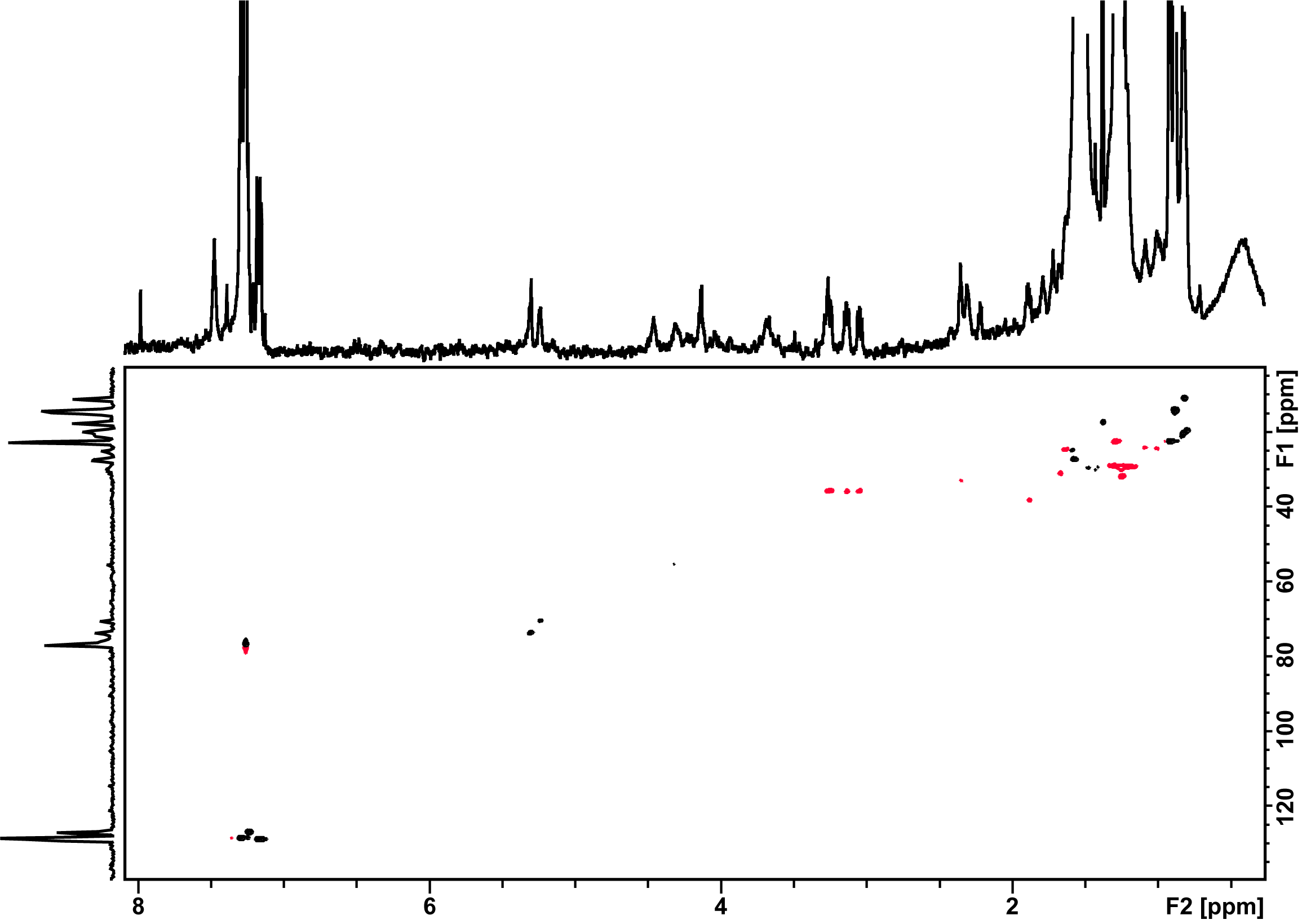

Supplement: S34 Fig — CDCl3, 800 MHz, TCI, 3 mm tube. (TIF) [file pone.0303273.s035.tif]

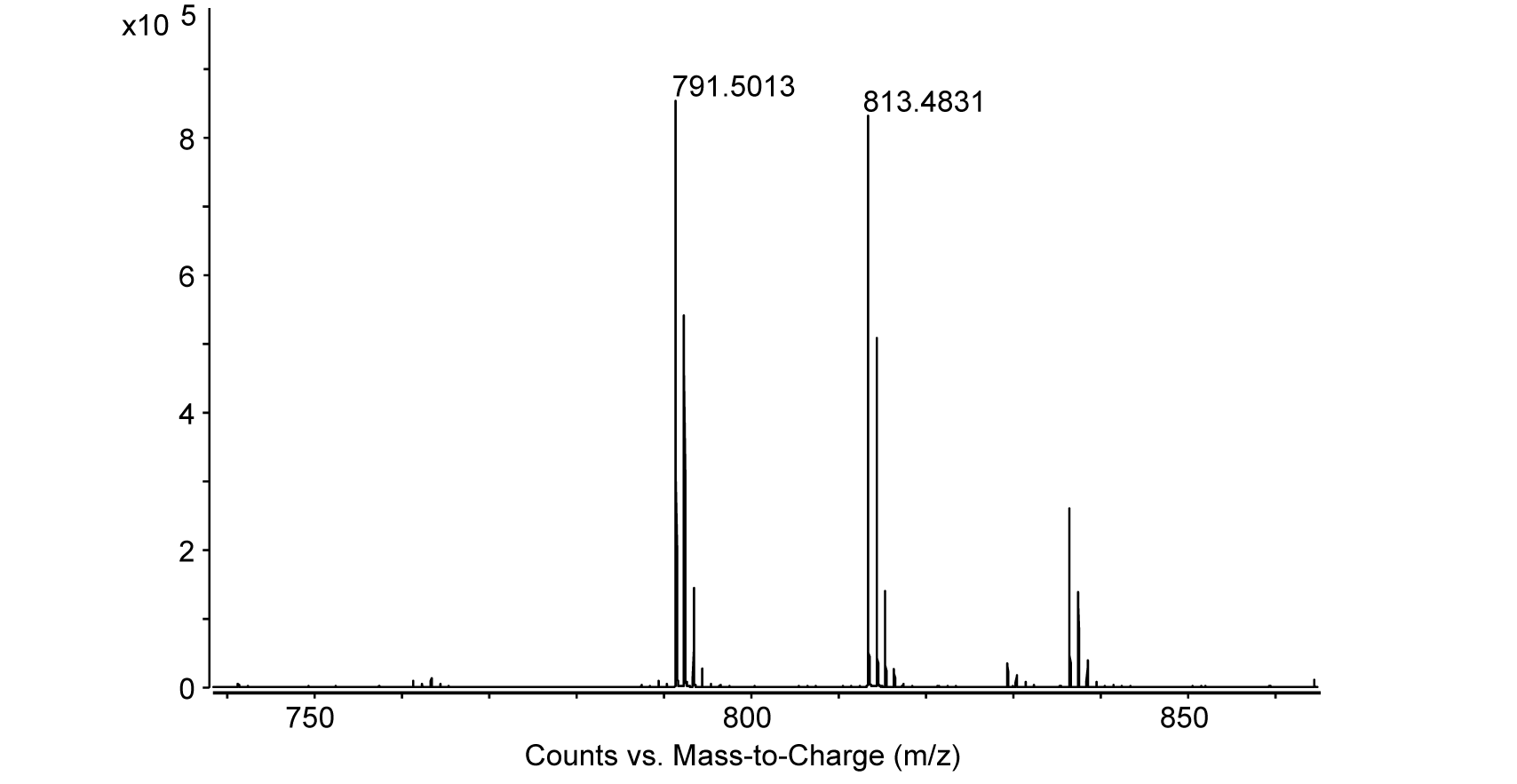

Supplement: S35 Fig — m/z 791.5013, MS feature 8190, [M+H]+; m/z 813.4831, MS feature 8184, [M+Na]+. (TIF) [file pone.0303273.s036.tif]

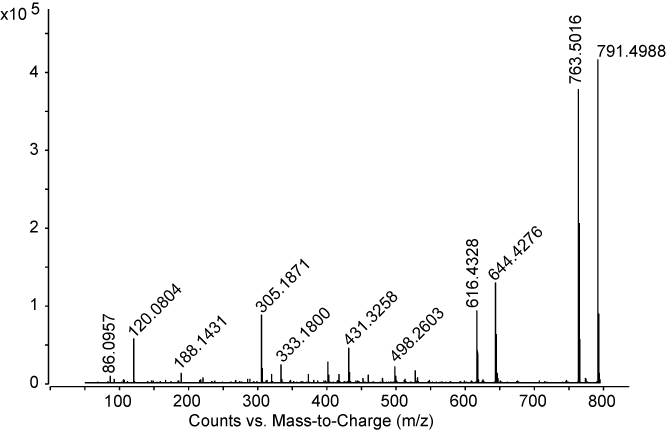

Supplement: S36 Fig — (TIF) [file pone.0303273.s037.tif]

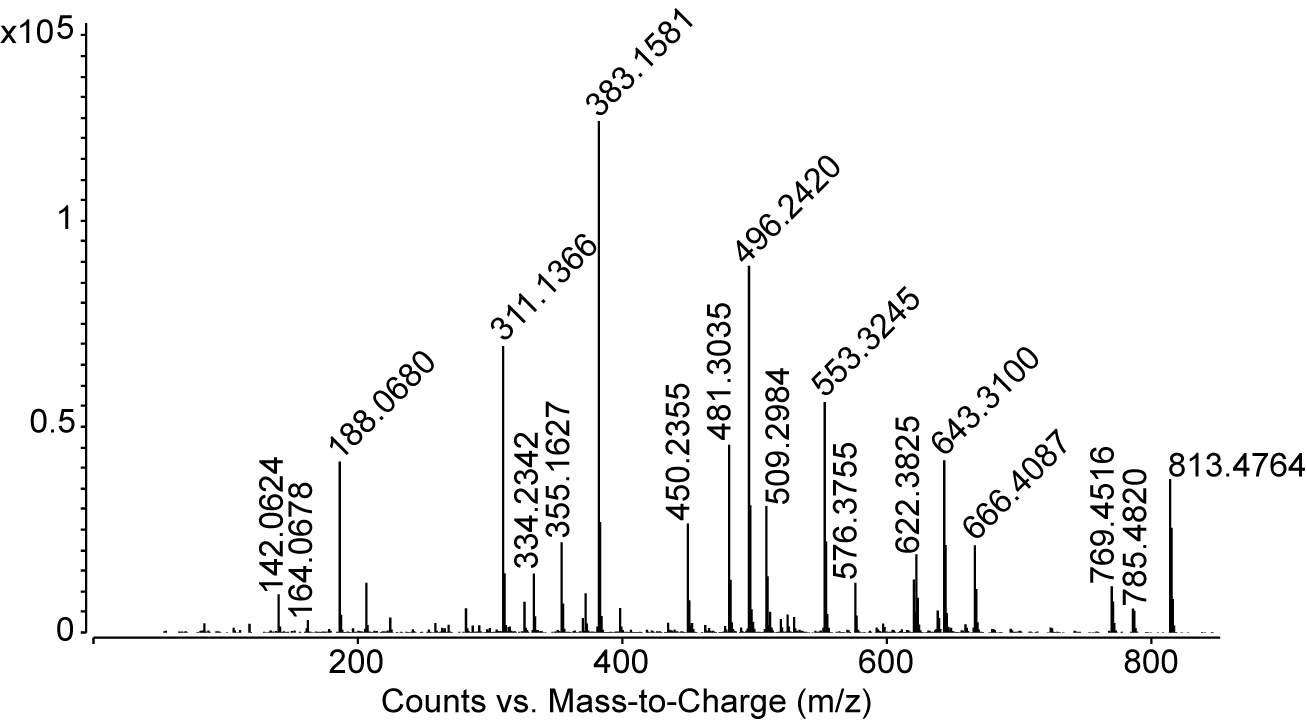

Supplement: S37 Fig — (TIF) [file pone.0303273.s038.tif]

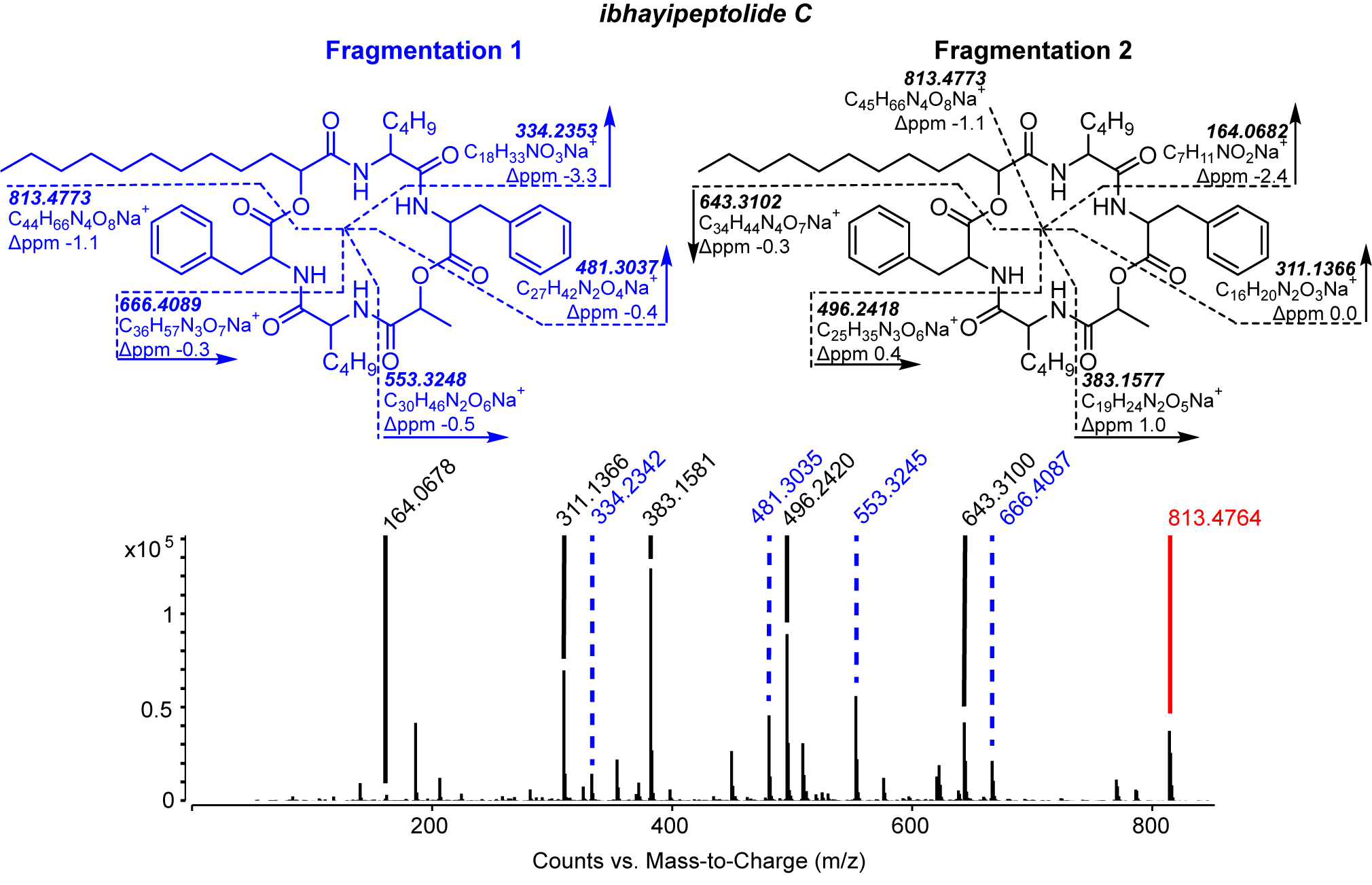

Supplement: S38 Fig — Top) Predicted fragmentation with theoretical mass and ppm error of fragment ions. Bottom) MS2 spectra ([M+Na]+, 60eV) labelled with HR mass (fragmentation 1: blue dotted lines, fragmentation 2: black solid lines). (TIF) [file pone.0303273.s039.tif]

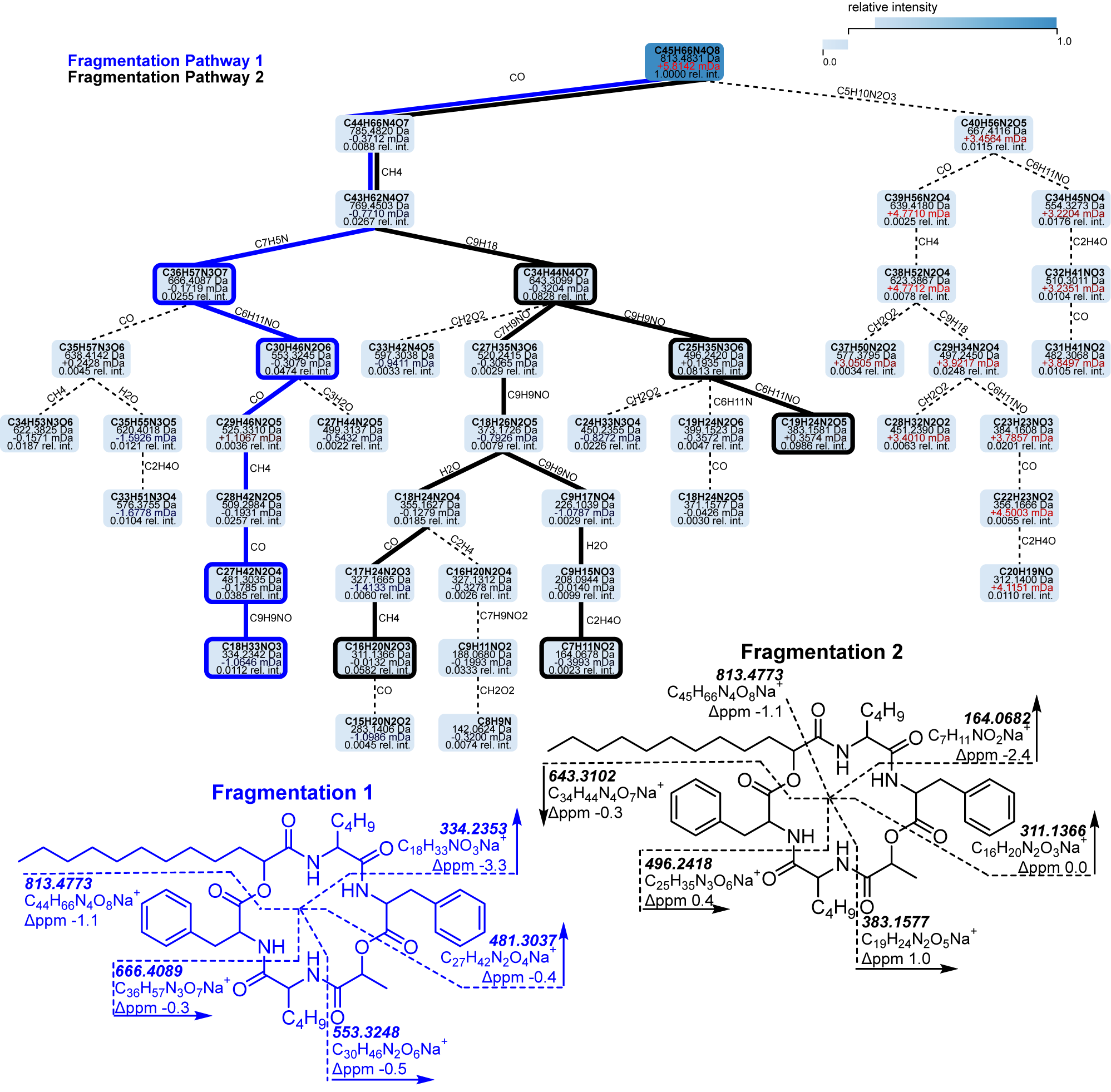

Supplement: S39 Fig — (TIF) [file pone.0303273.s040.tif]

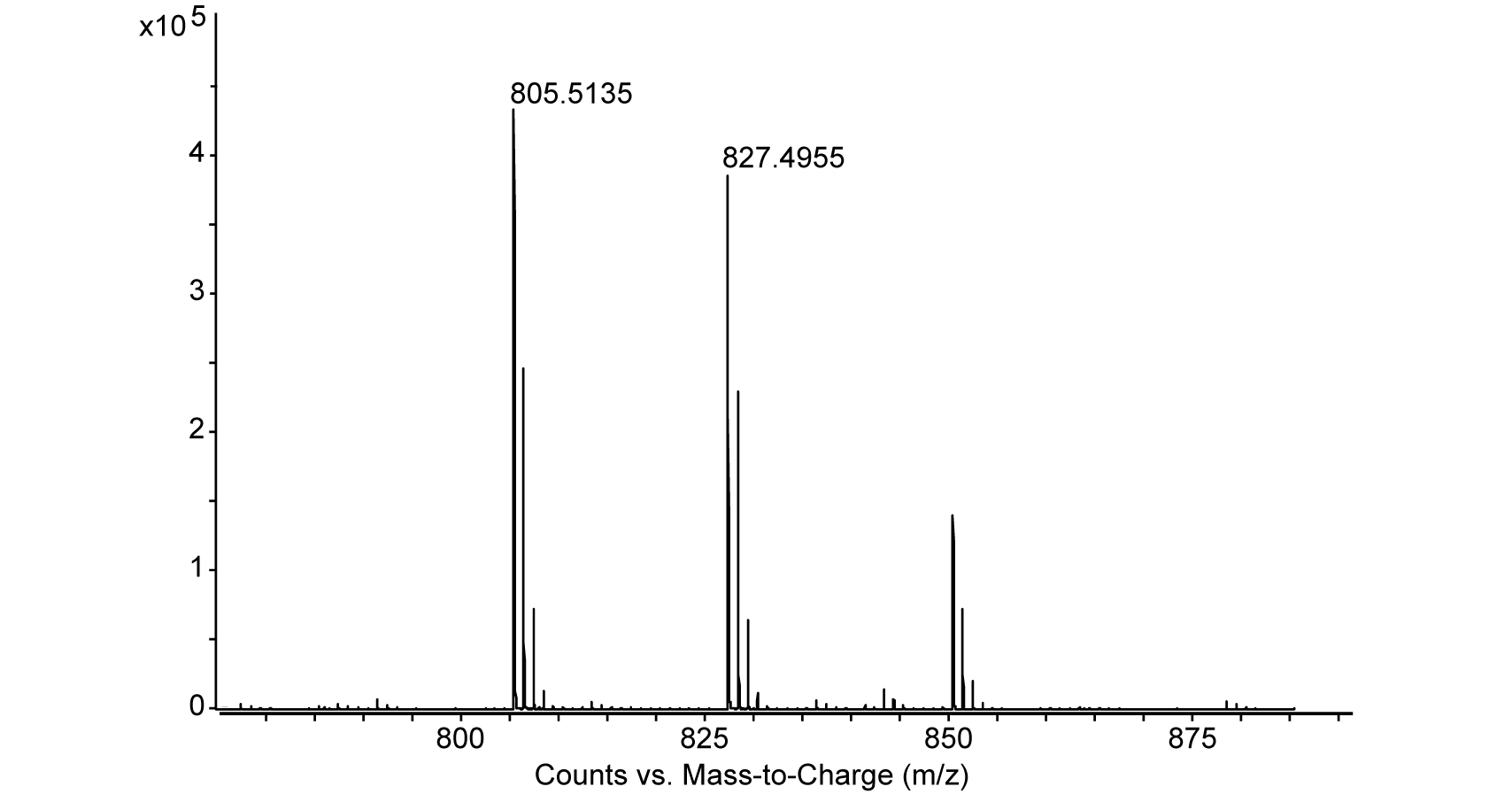

Supplement: S40 Fig — m/z 805.5135, MS feature 8185, [M+H]+; m/z 827.4955, MS feature 2473, [M+Na]+. (TIF) [file pone.0303273.s041.tif]

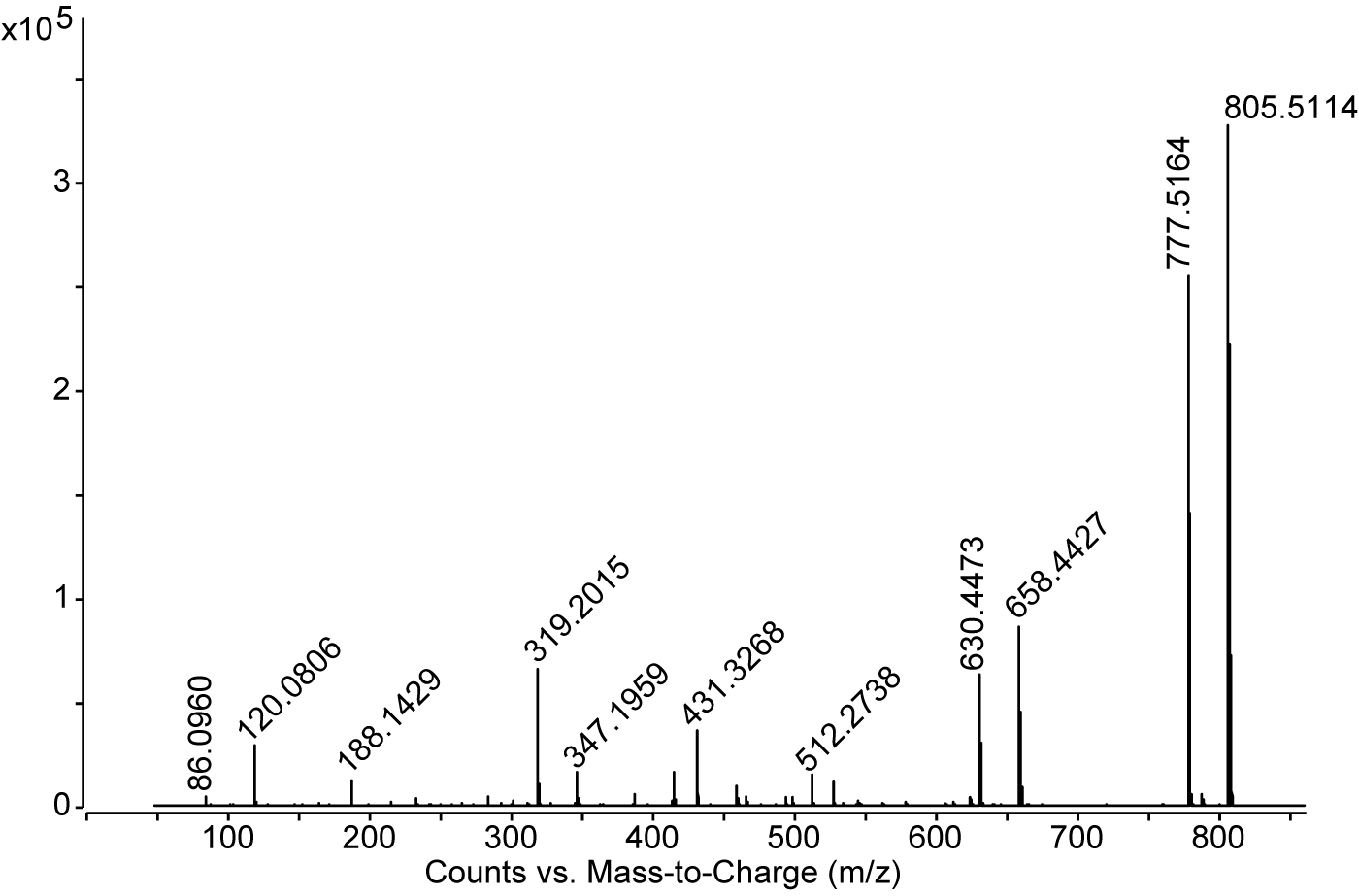

Supplement: S41 Fig — (TIF) [file pone.0303273.s042.tif]

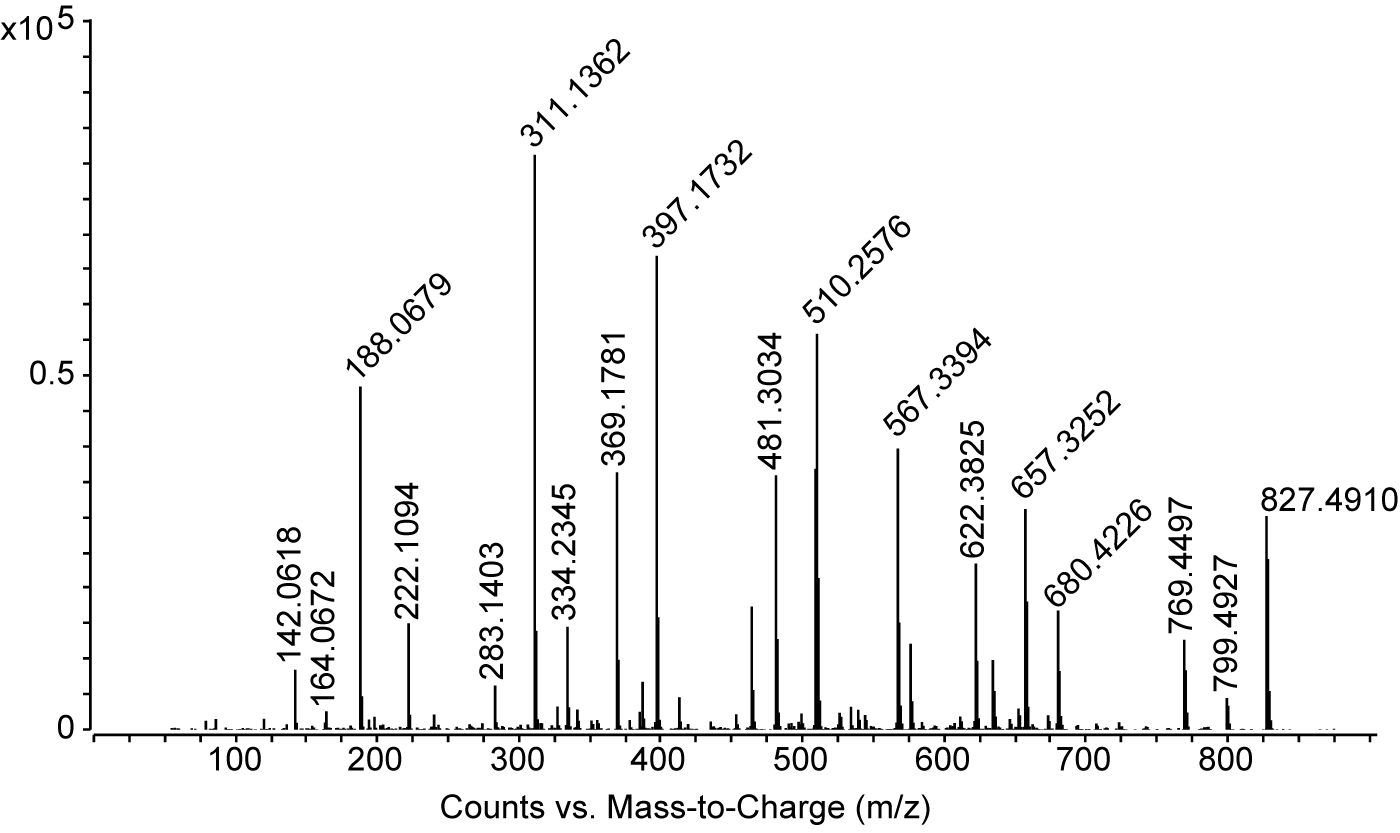

Supplement: S42 Fig — (TIF) [file pone.0303273.s043.tif]

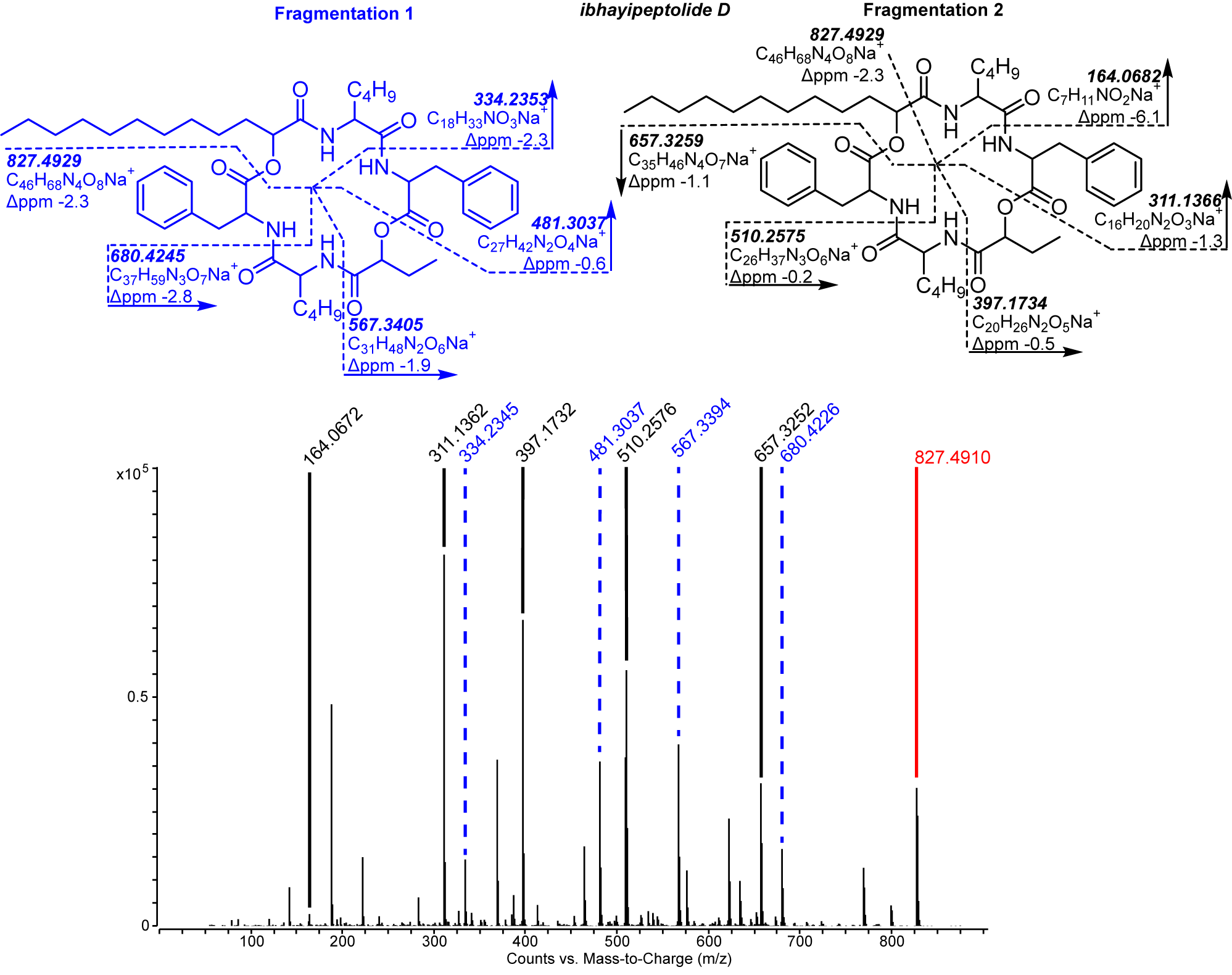

Supplement: S43 Fig — (TIF) [file pone.0303273.s044.tif]

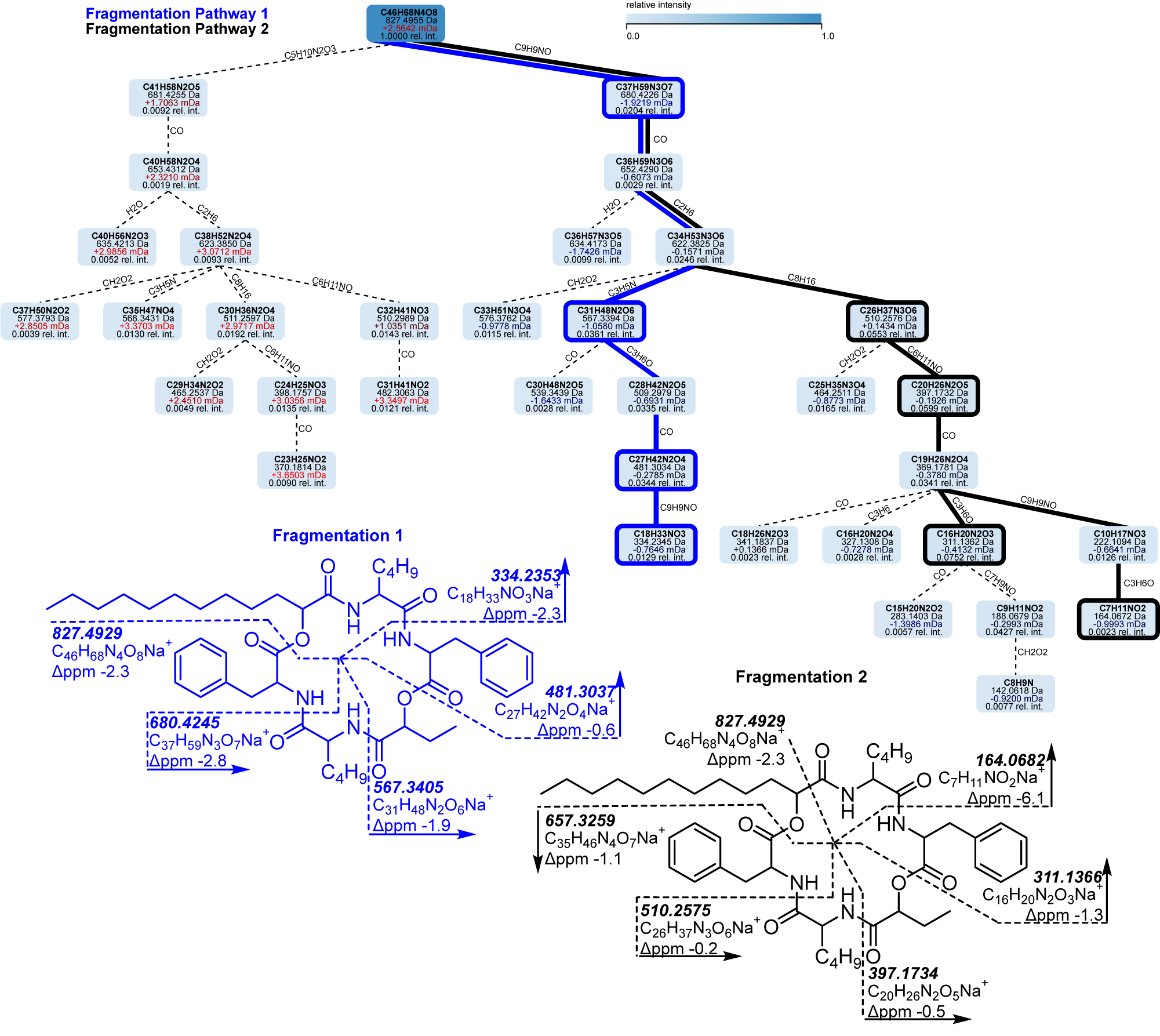

Supplement: S44 Fig — (TIF) [file pone.0303273.s045.tif]
