# Supplementary material for: Environmental metabolomics characterization of modern stromatolites and annotation of ibhayipeptolides
Source: PLoS One. 2024 May 23;19(5):e0303273. doi: 10.1371/journal.pone.0303273 (PMC11115249; doi:10.1371/journal.pone.0303273)
Supplement: S2 Table — CDCl3, 800 MHz, TCI probe, 3 mm tube. (DOCX) [file pone.0303273.s047.docx]

S2 Table. Proposed NMR chemical shift assignments for ibhayipeptolide A. CDCl_3_, 800 MHz, TCI probe, 3 mm tube.

| Residue  hydroxy acid | Atom | δ_C_, type | δ_H_ (*J* in Hz) |
| --- | --- | --- | --- |
| Phe #1 | carbonyl | - | - |
|  | α | 54.9, CH | 4.27, br s |
|  | β | 35.7, CH_2_ | 3.30, dd (14.0, 6.6) |
|  |  |  | 3.13, dd (14.0, 9.6) |
|  | γ | - | - |
|  | δ, δ^1^ | 128.8, CH | 7.15, d (7.5) |
|  | ε, ε^1^ | 128.5, CH | 7.29, t (7.5) |
|  | ζ | 127.0, CH | 7.24, m |
|  | NH | - | - |
| Leu | carbonyl | - | - |
|  | α | 52.3, CH | 4.19, q (7.5) |
|  | β | 38.2, CH_2_ | 1.82, m |
|  |  |  | 1.73, m |
|  | γ | 24.6, CH | 1.58, m |
|  | δ | 22.2, CH_3_ | 0.89, m |
|  | δ^1^ | 22.4, CH_3_ | 0.93, d (6.6) |
|  | NH | - | 7.45 |
| 2-Hba | carbonyl | - | - |
|  | α | 74.7, CH | 5.15, br s |
|  | β | 24.4, CH_2_ | 1.81, m |
|  |  |  | 1.75, m |
|  | γ | 8.5, CH_3_ | 0.70, t (7.4) |
| Phe #2 | carbonyl | - | - |
|  | α | 54.3, CH | 4.52, br s |
|  | β | 35.8, CH_2_ | 3.20, dd (14.3, 7.2) |
|  |  |  | 3.05, dd (14.3, 9.3) |
|  | γ | - | - |
|  | δ, δ^1^ | 128.8, CH | 7.19, d (7.5) |
|  | ε, ε^1^ | 128.5, CH | 7.29, t (7.5) |
|  | ζ | 127.0, CH | 7.24, m |
|  | NH | - | - |
| Ile | carbonyl | - | - |
|  | α | - | 3.67, m |
|  | β | 34.2, CH | 2.36, m |
|  | γ | 26.1, CH_2_ | 1.29, m |
|  |  |  | 1.01, m |
|  | γ^1^ | 14.5, CH_3_ | 0.89, obs |
|  | δ | 10.9, CH_3_ | 0.84, t (7.5) |
|  | NH | - | 7.48, d (7.3) |
| 2-Hda | carbonyl | - | - |
|  | α | 73.7, CH | 5.34, br s |
|  | β | 31.0, CH_2_ | 1.84, m |
|  |  |  | 1.72, m |
|  | γ | 24.3, CH_2_ | 1.12, m |
|  |  |  | 1.07, m |
|  | δ*^c^* | - | - |
|  | ε*^c^* | - | - |
|  | ζ*^c^* | - | - |
|  | η | 31.6, CH_2_ | 1.24, m |
|  | θ | 22.5, CH_2_ | 1.29, m |
|  | ι | 14.0, CH_3_ | 0.87, obs |
