# Supplementary material for: Environmental metabolomics characterization of modern stromatolites and annotation of ibhayipeptolides
Source: PLoS One. 2024 May 23;19(5):e0303273. doi: 10.1371/journal.pone.0303273 (PMC11115249; doi:10.1371/journal.pone.0303273)
Supplement: S3 Table — (DOCX) [file pone.0303273.s048.docx]

S3 Table. Pre-processing settings for MS feature detection using MZmine2.

| Mass detection | MS1 | 1.00E+05 |
| --- | --- | --- |
|  | MS2 | 1.00E+02 |
| Chromatogram building (ADAP) | min group size in # scans | 5 |
|  | group intensity threshold | 1.00E+05 |
|  | Min highest intensity | 3.00E+05 |
|  | m/z tolerance | 0.001 or 10 ppm |
| Deconvolution (Local minimum search algorithm) | Chromatographic Threshold | 1.00% |
|  | Search minimum in RT range (min) | 0.2 |
|  | Minimum relative height | 1.00% |
|  | Minimum absolute height | 3.00E+05 |
|  | Min ratio of peak top/edge | 1 |
|  | Peak Duration range (min) | 0.01-1.5 |
|  | m/z center calculation | median |
|  | m/z range for MS2 scan pairing | 0.01 Da |
|  | RT range for MS2 scan pairing | 0.15 min |
| Isotopic peak grouper | m/z tolerance | 0.001 or 10 ppm |
|  | RT tolerance | 0.2 min |
|  | Max charge | 3 |
|  | Most representative | most intense |
| Alignment (Join Aligner) | m/z tolerance | 0.001 or 10 ppm |
|  | Weight for m/z | 75 |
|  | Weight for RT | 25 |
|  | RT tolerance | 0.1 min |
| Gap filling (Multithreaded) | Intensity tolerance | 10% |
|  | m/z tolerance | 5 ppm |
|  | RT tolerance | 0.1 min |
| metaCorrelate | RT tolerance | 0.1 min |
|  | Min height | 0 |
|  | Noise level | 0 |
|  | Min data points in group | 5 |
|  | Min data points on edge | 2 |
|  | Measure | Pearson |
|  | Min MS feature shape correlation | 85% |
|  | Feature height correlation | checked |
|  | Min data points | 3 |
|  | Measure | Pearson |
|  | Min correlation | 60% |
| Ion Identity networking | m/z tolerance | 0.001 or 10 ppm |
|  | Min height | 100000 |
|  | MS mode | positive |
|  | Maximum charge | 2 |
|  | Maximum molecule/cluster | 2 |
|  | adducts | M+H |
|  |  | M+Na |
|  |  | M+K |
|  |  | M+NH4+ |
|  |  | M+Fe3-2H |
