# Supplementary material for: Environmental metabolomics characterization of modern stromatolites and annotation of ibhayipeptolides
Source: PLoS One. 2024 May 23;19(5):e0303273. doi: 10.1371/journal.pone.0303273 (PMC11115249; doi:10.1371/journal.pone.0303273)
Supplement: S5 Table — (DOCX) [file pone.0303273.s050.docx]

S5 Table. SIRIUS4 parameters for Thermo QExactive Orbitrap.

| SIRIUS parameters | |
| --- | --- |
| Instrument | Orbitrap |
| Filter by isotope pattern | yes |
| MS/MS isotope scorer | ingnore |
| MS/MS mass accuracy | 10 ppm |
| Candidates Stored | 10 |
| Min candidates per ion | 1 |
| Use heuristic above m/z | 300 |
| Use heuristic only above m/z | 650 |
